# Nonzero spontaneous electric polarization in metals: novel predictive methods and applications

**DOI:** 10.1038/s41598-023-49463-w

**Published:** 2024-01-05

**Authors:** Shahrbano Rahimi, S. Jalali-Asadabadi, Peter Blaha, Farhad Jalali-Asadabadi

**Affiliations:** 1 Department of Physics, Faculty of Physics, University of Isfahan (UI), Hezar Jerib Avenue, Isfahan, 81746-73441 Iran; 2 Institute of Materials Chemistry, Vienna University of Technology, Getreidemarkt 9/165-TC, 1060 Vienna, Austria

**Keywords:** Ferroelectrics and multiferroics, Electronic properties and materials

## Abstract

Ferroelectricity in metals has advanced since the initial discovery of nonmagnetic ferroelectric-like metal LiOsO$_3$, anchored in the Anderson and Blount prediction. However, evaluating the spontaneous electric polarization (SEP) of this metal has been hindered by experimental and theoretical obstacles. The experimental challenge arises from difficulties in switching polarization using an external electric field, while the theoretical limitation lies in existing methods applicable only to nonmetals. Zabalo and Stengel (Phys Rev Lett 126:127601, 2021, 10.1103/PhysRevLett.126.127601) addressed the experimental obstacle by proposing flexoelectricity as an alternative for practical polarization switching in LiOsO$_3$, which requires a critical bending radius similar to BaTiO$_3$. In this study, we focus on resolving the theoretical obstacle by modifying the Berry phase and Wannier functions approaches within density functional theory plus modern theory of polarization. By employing these modifications, we calculate the SEP of LiOsO$_3$, comparable to the polarization of BaTiO$_3$. We validate our predictions using various ways. This study confirms the coexistence of ferroelectricity and metallicity in this new class of ferroelectric-like metals. Moreover, by addressing the theoretical limitation and providing new insights into polarization properties, our study complements the experimental flexoelectricity proposal and opens avenues for further exploration and manipulation of polarization characteristics. The developed approaches, incorporating modified Berry phase and Wannier function techniques, offer promising opportunities for studying and designing novel materials, including bio- and nano-ferroelectric-like metals. This study contributes to the advancement of ferroelectricity in metals and provides a foundation for future research in this exciting field.

## Introduction

The spontaneous electric polarization (SEP) in metals was unexpected prior to 1965, challenging the understanding of long-range dipole order in metals and their equipotential nature^1,2^. However, in 1965, Anderson and Blount (A &B) predicted the possibility of ferroelectricity in metals based on Landau’s theory of second-order phase transitions^3,4^. Since then, the field of ferroelectric-like metals (FE-LMs) has witnessed significant growth, with contributions from researchers in diverse areas investigating a variety of FE-LMs^5–14^.

The A&B prediction was successfully validated by Shi et al.^15^, who discovered that lithium osmate (LiOsO$_3$) exhibits a continuous shift in the mean position of Li$^+$ ions below 140 K, confirming its classification as a new class of ferroelectric (FE) materials. This groundbreaking discovery was also acknowledged by Veerle Keppens in a news article, marking the first observation of a ‘ferroelectric metal’^16^. Since then, the continuous centrosymmetric (CS) R$\bar{\text {3}}$c to noncentrosymmetric (NCS) R3c phase transition in the ferroelectric-like (FE-L) LiOsO$_3$ metal has attracted significant theoretical and experimental attention^17–29^. This compound has exhibited rare properties consistent with the predictions of A &B, contributing to its significance in the field.

Notably, SEP, a fundamental metric for ferroelectric materials, has been theoretically assessed in hypothetical nonmetallic states of LiOsO$_3$^22,27^. However, its evaluation in the actual metallic state remains pending, hindered by two obstacles. The first obstacle pertains to experimental difficulties in switching polarization using an external electric field. The second obstacle is the theoretical limitations of existing methodologies applicable only to nonmetals.

Concerning the first obstacle, Zabalo and Stengel^30^ very recently have investigated the potential of using flexoelectricity to switch the polarization of LiOsO$_3$. Flexoelectricity refers to the phenomenon where electric polarization can be induced by strain gradients in a material. By applying a specific type of strain gradient to LiOsO$_3$, the polarization direction can be switched without the need for an external electric field. Their groundbreaking study, estimating that the critical bending radius for polarization switching in LiOsO$_3$ is of the same order of magnitude as that of BaTiO$_3$, serves as an experimental alternative to address the first obstacle, opening new possibilities for practical polarization switching in LiOsO$_3$. Liu et al.^21^ found that LiOsO$_3$ exhibits metallic ferroelectricity characterized by highly anisotropic screening, unscreened local dipole interactions, order-disorder type transition, and a triggering mechanism related to hybridization effects. Xiao et al.^31^ observed the ferroelectric transition in LiOsO$_3$ through the nonlinear Hall effect, which revealed a strong dependence on polar displacement and proposed it as a method for detecting polar order. Sharma et al.^13^ successfully switched the polarization of a ferroelectric metal by applying an electric field and suppressing current flow using a dielectric layer. Wing Chi et al.^32^ demonstrated that LiOsO$_3$ can serve as a platform for exploring topological phases and their interplay with ferroelectric ordering. In contrast to normal metals, the ferroelectric-like phase in LiOsO$_3$ exhibits dipole moment due to the confinement of charge carriers in Weyl nodes. Ronghan Li et al.^33^ showed that HgPbO$_3$ exhibits a ferroelectric phase transition despite being a semimetal, highlighting the possibility of Weyl ferroelectric metals with cooperative atomic displacements. The unique characteristics of Weyl semimetals, such as lower carrier density and weaker electrostatic screening, enable the formation of electric dipoles and the potential for Weyl ferroelectric metals.

Due to the above pieces of evidence on the first obstacle, here, we concentrate only on the second obstacle by investigating that whether SEP and metallicity can also coexist in the FE-L lithium osmate metal. To this end, since LiOsO$_3$ is a metal, it is enough to make sure that it is also a FE. To ensure that the LiOsO$_3$ system can be considered as an acceptable FE material, besides the properties reported earlier^15–29^ it is also crucial to know its SEP as a vital-character of ferroelectricity^34–37^. However, the following literature review shows that the SEP has been calculated only for a hypothetical insulating form of this material due to the numerical limitations of the conventional polarization schemes. Chao He et al.^22^, calculated the SEP for a hypothetical nonmetallic (NM) G-type-antiferromagnetic (G-AFM) state of LiOsO$_3$ after opening its bandgap by LDA+U^38–41^ in the framework of the Berry phase (Bp) scheme^42–45^ as implemented in the pseudopotential-based VASP code^46–50^. Yu Zhang et al.^27^, recently, calculated the effects of strain on the SEP of the hypothetical nonmetallic state of LiOsO$_3$ after opening the bandgap of this metal by imposing tensile biaxial strains and G-AFM ordering employing LDA+U and LSDA approaches using the BP scheme^42–45^ as implemented in the VASP code^46–50^. However, the SEP, as a fundamental and main physical quantity of a FE material, has not been reported for this interesting FE-LM in its factual metallic state yet. This motivated us to calculate the SEP for this new class of FE-L material. However, the existing polarization methods are inapplicable to calculate the SEP of metals, as discussed below.

In the framework of the modern theory of polarization^51–59^, there are two standard approaches to calculate the SEP for a material. The first approach is the Bp method^42–45^. The second approach is the Wannier functions (Wf) method^60–65^. In these methods, it is traditionally assumed that all the valence bands contributed in the electronic part of the polarization are completely occupied. This makes them inapplicable for metals containing valence bands with fractional occupation numbers in the vicinity of their Fermi levels. Therefore, in practice, neither Bp nor Wf method in their standard forms is applicable to calculate the electric polarization of a metal. To find a practical solution for the above problem, we, first, modify the conventional Bp and Wf methods of polarization, called mBp and mWf, and enable them to calculate the SEP of FE-LMs, see Sect. 1 of Supplementary Materials (SMs). We then calculate the SEP for the LiOsO$_3$ FE-LM by our proposed mBp and mWf methods. Second, we uniquify the SEP by considering $\pi$-wrapping problem^57,66^. To this end, we recalculate the SEPs using 9 intermediate superstructures, in addition to the initial CS (non-polar) and final NCS (polar) structures of the metallic state of LiOsO$_3$. Consequently, the best branch is chosen and the SEP is determined uniquely. The computed SEP we have determined exhibits a magnitude on par with that observed in BaTiO$_3$, as documented in studies by Abrahams et al.^67^ and Merz^68^. This finding closely aligns with the data concerning the critical bending radius for polarization switching, as reported by Zabalo and Stengel^30^, thereby positioning our results within the same domain of these established benchmarks in the field.

Furthermore, we systematically validate our predicted SEP (1) numerically by showing the constancy between the SEPs predicted by the two different approaches mBp and mWf, (2) empirically by successfully fitting the results to the available empirical equations proposed by Abrahams et al.^67^ and our empirical equations emerged from various available experimental transition temperatures and SEPs of the normal ferromagnetics, (3) phenomenologically by fitting to the phenomenological equation proposed by Landau-Ginzburg, (4) hypothetically by opening the bandgap of the metal in question using GGA+U. After opening the bandgap, the SEP is calculated by the conventional Bp method without any modifications and found it consistent with our prediction made by the mBp proposed in this work. Using another different approach, the bandgap is opened by imposing distortion instead of GGA+U. Here, by the neural network the SEP is predicted at zero strain using the available SEPs at nonzero biaxial strains imposed on the system. The prediction made by the neural network is found in agreement with our SEP predicted by mBp method of polarization. All these show that the different but consistent mBp and mWf methods provide two novel approaches of polarization to satisfactorily calculate the electric polarization of the FE-LM within almost the same accuracy.

In essence, recent studies have made significant progress in addressing the first obstacle hindering the investigation of ferroelectric-like metals (FE-LMs), which is the experimental difficulty in polarization switching using an external electric field. Researchers have explored alternative methods, such as flexoelectricity, to overcome this challenge. However, the second obstacle, related to the theoretical limitations of existing methodologies applicable only to nonmetals, still remains. Our study contributes to the advancement of ferroelectricity in metals by addressing this second obstacle and providing new insights and approaches. This opens up avenues for further exploration and understanding of polarization characteristics in FE-LMs, offering new opportunities for advancements in this field.

## Band classification and introduction to polarization methods for LiOsO$_{{\textbf {3}}}$

**Figure 1:**
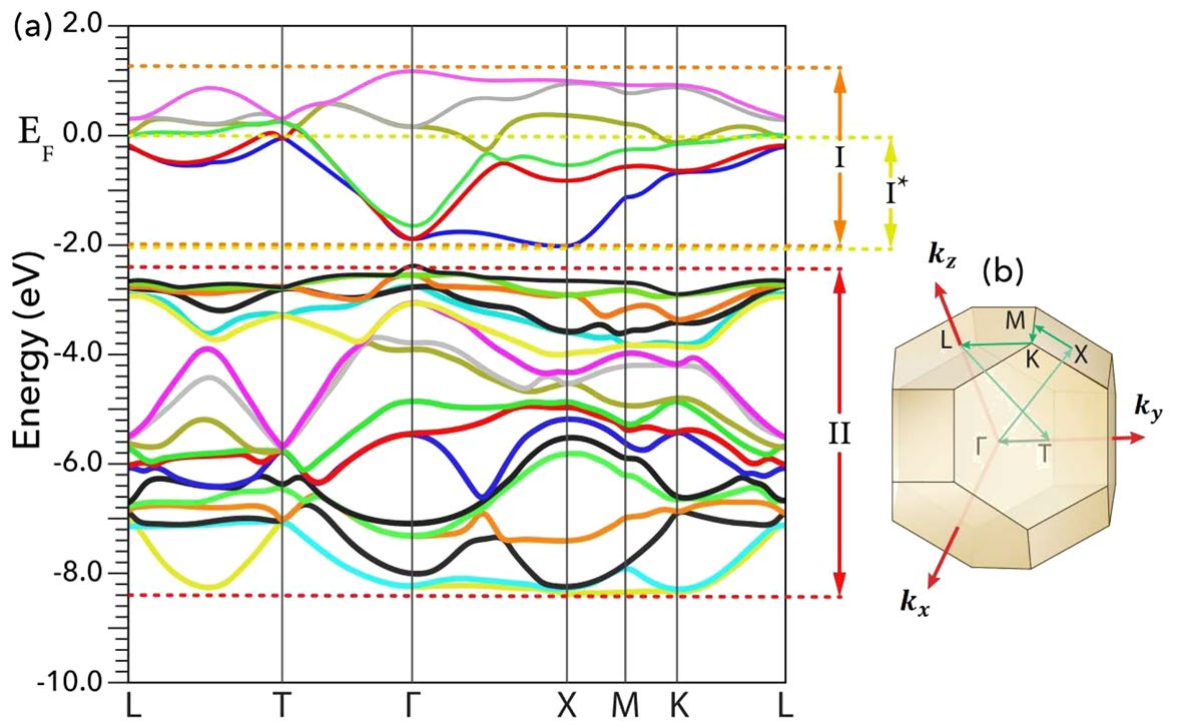
(**a**) Band structure of the polar NCS R3c phase of the LiOsO$_3$ compound constructed by the rhombohedral (trigonal) crystal system calculated using the PBE-GGA^69^. (**b**) The corresponding first Brillouin zone including the symmetrical points and selected paths, as plotted by XCrySDen package^70^.

Both modified polarization methods, mBp and mWf, begin with band classification as their initial step. We will first cover this common step before individually discussing the subsequent steps for each method. To initiate our methodology, we will classify the bands of LiOsO$_3$ as calculated using the PBE-GGA functional for its rhombohedral structure, illustrated in Fig. 1.

### Classification of bands

**Figure 2:**
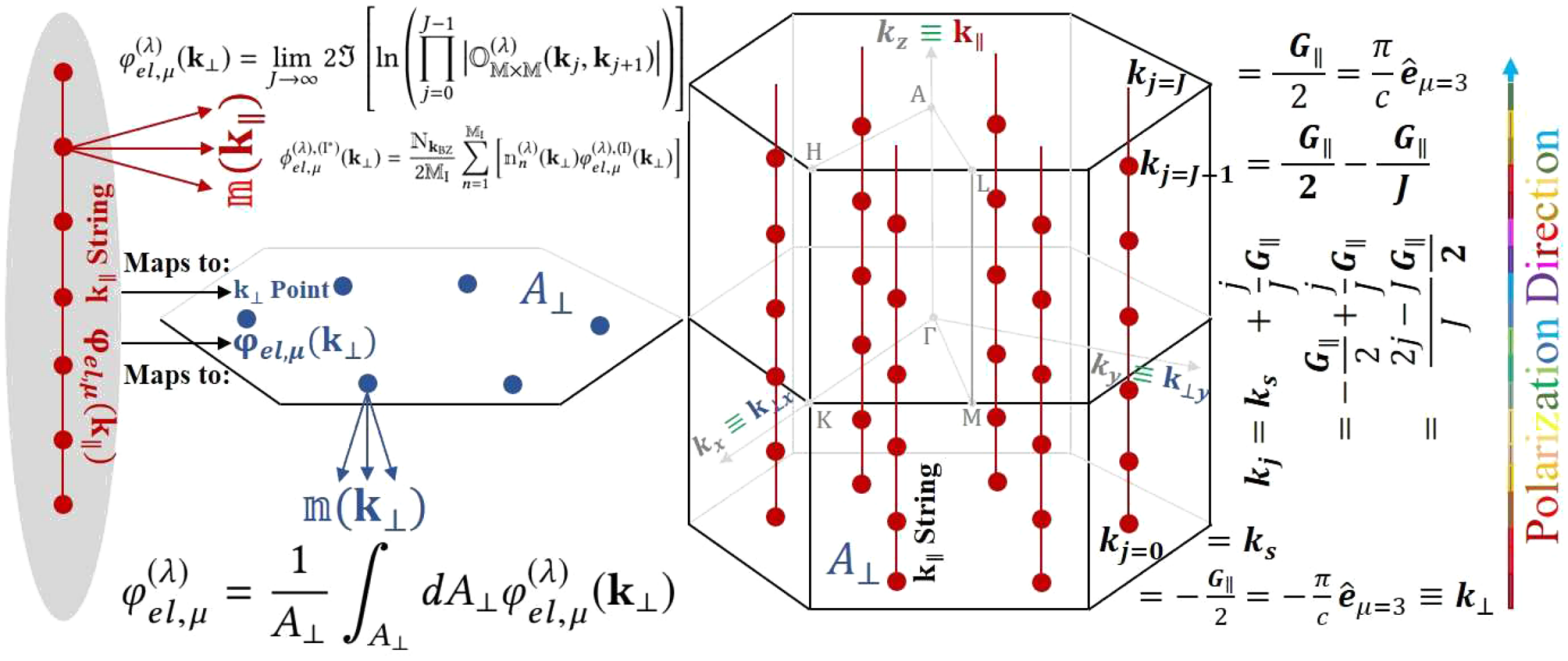
The first Brillouin zone used for the calculations of the electronic part of the Berry phase in the reciprocal lattice, decomposing ${\textbf {k}}$-points into ${\textbf {k}}_\parallel$ and ${{\textbf {k}}_\bot }$ samples. Here, ${\textbf {k}}_\parallel$ is parallel to and ${{\textbf {k}}_ \bot }$ is perpendicular on the polarization direction ${\textbf {G}}_\mu$, as shown on the right side of the figure. Several 2D-planes are formed by the ${{\textbf {k}}_\bot }$ with the normal vectors ${\textbf {k}}_\parallel$. The points ${\textbf {k}}_\parallel$ are distributed over the parallel strings. Occupation numbers of the bands at ${\textbf {k}}_\parallel$ are indicated by $\mathbbm {n}({\textbf {k}}_\parallel )$. The contribution of the electronic Berry phase for the structure $\lambda$ at ${\textbf {k}}_\parallel$ are shown by $\upvarphi _{el, \upmu }^{(\lambda )}({\textbf {k}}_\parallel )$. By the formula, as indicated in the top-left side of the figure, and considering $\upvarphi _{el, \upmu }^{(\lambda )}({\textbf {k}}_\parallel )$, included in the overlap integral $\mathbb {O}_{\mathbb {M}\times \mathbb {M}}^{(\lambda )}({\textbf {k}}_j, {\textbf {k}}_{j+1})$, the Berry phases are calculated along each ${\textbf {k}}_\parallel$ strings individually, and the results for the structure $\lambda$ are mapped as $\upvarphi _{el, \upmu }^{(\lambda )} ({\textbf {k}}_\perp )$ into the central 2D-sheet with the area $A_\perp$. Occupation numbers of the bands at ${\textbf {k}}_\perp$ are indicated by $\mathbbm {n}({\textbf {k}}_\perp )$ By the formula, as indicated in the bottom-left side of the figure, the average of the mapped Berry phases are calculated over the area $A_\perp$. For more detail see Sect. 1.1 of SMs.

Classification of the valence and conduction bands constitutes the zeroth step of our modified methods of polarization.

In the zeroth step, we classify the bands into three classes, as shown in Fig. 1. These classes are labeled by I, I$^*$ and II in Fig. 1. These labels are dual-purpose and, in addition to their roles in classifying the bands, they can be also used to distinguish three different energy intervals indicated in Fig. 1. The class I includes valence bands which are crossing the Fermi level and conduction bands. The highest energy limit of class I$^*$ is the Fermi energy, while its lowest energy limit possesses in common with that of class I. Therefore, the class I$^*$ is a subclass of class I. Only some of the valence bands in classes I and I$^*$ are full while their other remaining valence bands are partially filled and hence they can only partially contribute into the electronic part of the Berry phase, as expressed in Eq. (12) of the SMs. The conduction bands in class I are empty and hence their contributions into the electronic part of the EP are zero. The class II includes valence bands which are all fully occupied. The valence bands included in class II are well separated by an energy interval of $\approx 0.4$ eV from the bands included in class I, see Fig. 1.

After the classification, we introduce our polarization methodologies: the mBp and mWf methods. We begin with the six-step mBp polarization method. For a more detailed view, refer to Sect. 3 of the SMs.

### Mean-field-like mBp method of polarization

To overcome the obstructs, as discussed in Sect. 2 of the SMs, and calculate the SEP of a FE-L metal, we modify the conventional Berry phase method, beginning after the zeroth step. The critical points of the proposed methods is concisely provided in the next six steps. For a comprehensive discussion, refer to Sect. 3.1 of the SMs.

In the first step, we obtain the ionic part of the Berry phase, $\varphi _{ion,\mu }^{\left( \lambda \right) }$, for the structure $\lambda$ along polarization direction $\mu$ by applying the standard Berry phase approach over the density functional theory (DFT) results. Here, $\lambda$, which can be 0 or 1, denotes the initial non-polar CS R$\bar{3}$c structure and the final polar NCS R3c phase, which has a lower symmetry than R$\bar{3}$c; i.e., $(\lambda =0) \equiv \text {R}\bar{\text {3}}\text {c}$ and $(\lambda =1) \equiv \text {R3c}$.

In the second step, using the standard Berry phase approach, we obtain the electronic part of the Berry phase in the $\mu$ direction for all bands of class II at every perpendicular wave vector ${\textbf {k}}_\perp$. This is illustrated in Fig. 2 for the structure $\lambda$, represented as $\varphi _{el,\mu }^{\left( \lambda \right) , \text {(II)}}({\textbf {k}}_\bot )$. We then adjust the electronic Berry phase of class II to be independent of ${\textbf {k}}_\perp$. To achieve this, we draw an analogy to the standard Berry phase method. As depicted in Fig. 2, we take an average either over the perpendicular area $A_\perp$ or over the discrete points $\mathbb {N}_{{\textbf {k}}_\bot }$ of the 2D ${\textbf {k}}_\bot$-point samples. This allows us to determine $\varphi {el,\mu }^{\left( \lambda \right) , \text {(II)}}$ as follows:

1 $$\begin{aligned} \varphi _{el,\mu }^{\left( \lambda \right) , (\text {II})}= & {} \frac{1}{A_\perp }\int _{A_\perp }dA_\perp \varphi _{el,\mu }^{\left( \lambda \right) , (\text {II})}({\textbf {k}}_\perp ) \nonumber \\\approx & {} \frac{1}{{{\mathbb {N}_{{k_\bot }}}}}\mathop \sum \limits _{\;{{{\textbf {k}}_\bot }}} \varphi _{el,\mu }^{\left( \lambda \right) , (\text {II})}({\textbf {k}}_\perp ), \end{aligned}$$

In the third step, we determine the electronic part of the Berry phase for all bands of class I, represented as $\varphi _{el,\mu }^{\left( \lambda \right) , \text {(I)}}({\textbf {k}}_\bot )$. We make a temporary assumption that all bands of this class are fully occupied. This assumption will be modified in the fourth step. At this stage, we delay the averaging process for $\varphi _{el,\mu }^{\left( \lambda \right) , (\text {I})}({\textbf {k}}_\perp )$ until the end of the fourth step. This is when the modification occurs. Hence, for now, it remains dependent on ${\textbf {k}}_\perp$.

In the fourth step, we adjust $\varphi _{el,\mu }^{\left( \lambda \right) , (\text {I})}({\textbf {k}}_\perp )$ by taking into account the correct occupation numbers for the class I bands, denoted as $\mathbbm {n}_n^{(\lambda )}({\textbf {k}}_\perp )$, as follows:

2 $$\begin{aligned} \phi _{el,\mu }^{\left( \lambda \right) , (\text {I}^*)}({\textbf {k}}_\perp )=\frac{\mathbbm {N}_{{\textbf {k}}_{\text {BZ}}}}{2\mathbb {M}_{\text {I}}} \sum _{n=1}^{\mathbb {M}_{\text {I}}}\left[ \mathbbm {n}_n^{(\lambda )}({\textbf {k}}_ \perp )\varphi _{el, \mu }^{\left( \lambda \right) , \text {(I)}}({\textbf {k}}_\perp )\right] , \end{aligned}$$

where $\phi$ stands for the modified $\varphi$, and $\mathbb {M}_{\text {I}}$ is the number of bands of class I, as well as $\mathbbm {N}_{{\textbf {k}}_{\text {BZ}}}^{(\lambda )}$ is the total number of ${\textbf {k}}$-points generated in the full mesh of the first Brillouin zone for structure $\lambda$, see Fig. 2. Now, it is time to make $\phi _{el,\mu }^{\left( \lambda \right) , (\text {I}^*)}({\textbf {k}}_\perp )$ independent of ${\textbf {k}}_\perp$ using the same averaging procedure outlined in Eq. (2) to derive $\phi _{el,\mu }^{\left( \lambda \right) , (\text {I}^*)}$.

In the fifth step, we combine the electronic Berry phases calculated in steps 2 and 4 to obtain the modified total electronic Berry phase for structure $\lambda$ in direction $\mu$. This is given by: $\phi _{el,\mu }^{\left( \lambda \right) } = \varphi _{el,\mu }^{\left( \lambda \right) , \text {(II)}} + \phi _{el,\mu }^{\left( \lambda \right) , (\text {I}^*)}$. To find the total Berry phase for structure $\lambda$ in direction $\mu$, denoted as $\phi _\mu ^{\left( \lambda \right) }$, we add the electronic Berry phase $\phi _{el,\mu }^{\left( \lambda \right) }$ to the ionic Berry phase for structure $\lambda$ in direction $\mu$ that was calculated and stored in step 1, represented as $\varphi _{ion,\mu }^{\left( \lambda \right) }$. That is, $\phi _\mu ^{\left( \lambda \right) } = 2\phi _{el,\mu }^{\left( \lambda \right) } + \varphi _{ion,\mu }^{\left( \lambda \right) }$, where the factor of 2 accounts for the spin degeneracy in non-spin-polarized systems. For spin-polarized systems, the expression is modified as: $\phi _\mu ^{\left( \lambda \right) } = \varphi _{ion}^{\left( \lambda \right) } + \varphi _{el}^{(\uparrow ), \left( \lambda \right) } + \varphi _{el}^{(\downarrow ), \left( \lambda \right) }$, where $(\uparrow )$ and $(\downarrow )$ denote spins up and down, respectively.

In the sixth step, we substitute $\varphi _\mu ^{\left( \lambda \right) }$ into the following equation:

3 $$\begin{aligned} P_\mu ^{\left( \lambda \right) } = \frac{e}{{2\pi }}\frac{{\;\varphi _\mu ^{\left( \lambda \right) }}}{{{{{\Omega }}^{\left( \lambda \right) }}}}\;R_\mu ^{\left( \lambda \right) }, \end{aligned}$$

where $e$ is the electron charge and ${\Omega }^{(\lambda )}$ is the unit cell volume of the structure $\lambda$. In Eq. (3), $R_\mu ^{\left( \lambda \right) }$ is the length of the lattice vector in real space for the structure $\lambda$, viz. ${\textbf {R}}^{\left( \lambda \right) } = \mathop \sum _{\mu = 1}^3 R_{\mu }^{\left( \lambda \right) } \hat{{\textbf {e}}}_\mu ^{\left( \lambda \right) }$, where $R_{\mu }^{\left( \lambda \right) }\hat{{\textbf {e}}}_\mu ^{\left( \lambda \right) }$($R_{\mu }^{\left( \lambda \right) })$ is the primitive vector (lattice constant) of structure $\lambda$ along $\mu$ in the direction of the unit vector $\hat{{\textbf {e}}}_\mu ^{\left( \lambda \right) }$.

Then, after multiplying both sides of Eq. (3) by $\hat{{\textbf {e}}}_\mu$ and taking the summation over $\mu$ on both sides of the resultant equation, we obtain the polarization vector for structure $\lambda$ as:

4 $$\begin{aligned} \sum _{\mu =1}^3P_{\mu }^{\left( \lambda \right) }\hat{{\textbf {e}}}_\mu =\textbf{P}^{(\lambda )}= \frac{e}{{2\pi \Omega ^{(\lambda )}}}\sum _{\mu =1}^3\varphi _\mu ^{\left( \lambda \right) }R_\mu ^{\left( \lambda \right) }\hat{{\textbf {e}}}_\mu . \end{aligned}$$

The procedure discussed above, from step 1 to this stage of step 6, is performed for both structures: one for structure $\lambda =0$ and the other for structure $\lambda =1$. In this manner, we obtain the electric polarization vectors $\textbf{P}^{(\lambda =0)}$ for the structure $\lambda =0$ and $\textbf{P}^{(\lambda =1)}$ for the structure $\lambda =1$.

Finally, the spontaneous polarization $\Delta {\textbf {P}}$ is determined as: $\Delta {\textbf {P}} = \textbf{P}^{(\lambda =1)} - \textbf{P}^{(\lambda =0)}$, employing the modern theory of polarization^51–59^.

### mWf method of polarization

The conventional Wannier functions scheme is not apt for FE-LMs as it presumes all bands to be fully filled, a condition not met by FE-LMs. Consequently, we employ the partly occupied maximally localized Wannier functions methodology introduced by Thygesen et al.^71,72^, further adapting the occupation numbers beginning after the zeroth step.

This method has shown that the degree of localization of Wannier functions can be optimized with a specific number of unoccupied orbitals^71,73^. Andrinopoulos et al.^74^ enhanced DFT’s van der Waals energy contributions using partly occupied MLWFs, incorporating anti-bonding states. For metals, considering only occupied states can result in poorly localized Wannier functions. However, adding unoccupied conduction states can significantly improve localization^65,71,73,74^. Although the maximally localized Wannier functions have been extended for partly occupied states^71,72,74^, their application for predicting electric polarization in FE-LMs is unexplored. Occupancy in polarization formulas is often assumed to be 2 for each Wannier center, but metals with partial occupation can have values less than 2. We find it pertinent to provide a succinct overview of this method for SEP calculations in FE-LMs, incorporating modifications to tackle these challenges. For an exhaustive discussion, readers are directed to Sect. 3.2 of the SMs.

In our research, we used the approach delineated by Thygesen et al.^71^ from 2005. This methodology is wave vector-independent, paving the way for its straightforward implementation in both isolated and periodic systems. On the other hand, an earlier method presented by Souza et al.^61^ in 2001 uses a ‘disentangling procedure’. This technique, while older, zeroes in on specific bands, aiming to reduce variations in the character of Bloch states across the Brillouin zone, with an emphasis on revealing pertinent unoccupied states. Although these methods vary, their shared objective revolves around the creation of more localized Wannier functions by examining the conduction and valence bands proximate to the Fermi level. In contexts where band groupings are not evident or when specific computational attributes of bands near the Fermi level are required (as in the calculation of the anomalous Hall conductivity via Wannier interpolation^75^), the method by Souza et al.^61^ might be the go-to choice. A testament to its utility is the work of Wang et al.^75^, where they applied a post-processing step to Bloch states near the Fermi level. Leveraging the Souza et al. technique^61^, they mapped these states onto localized Wannier functions, enabling the computation of the anomalous Hall conductivity. This approach facilitated precise Berry curvature interpolation for any selected k-point, striking a balance between computational efficacy and precision. However, in scenarios echoing our situation, where distinct band groups are discernible (as depicted in Fig. 1), the partly occupied approach by Thygesen et al.^71^ is potentially more beneficial. Hence, for the purposes of our study, we deem the newer partly occupied method^71^ to be aptly suited.

The total polarization vector $\textbf{P}^{(\lambda )}$ for structure $\lambda$ can be expressed in terms of its electronic $\textbf{P}_{el}^{(\lambda )}$ and ionic $\textbf{P}_{ion}^{(\lambda )}$ contributions within the Wannier functions framework as:

5 $$\begin{aligned} \textbf{P}^{(\lambda )}&= \textbf{P}_{ion}^{(\lambda )} + \textbf{P}_{el}^{(\lambda )} \nonumber \\&= \frac{e}{{\Omega }^{(\lambda )}} \left[ \sum _{s=1}^{\mathbb {N}} Z_s^{(\lambda )} \textbf{r} _s^{(\lambda )} + \sum _{n=1}^{\mathbb {J}} \mathbbm {n}_{W_{n, \varvec{R}}^{(\lambda )}} \langle \textbf{r} \rangle _{W_{n, \varvec{R}}^{(\lambda )}} \right] , \end{aligned}$$

where the first (second) term can be interpreted as ionic (electronic) polarization per unit volume ${\Omega }^{(\lambda )}$ of structure $\lambda$ originated from $\mathbb {N}$ ions ($\mathbb {J}$ Wannier centers) each with positive (negative) charges of $+eZ_s^{(\lambda )}$ ($-e\mathbbm {n}_{W_{n, {\textbf {R}}}^{(\lambda )}}$) positioned at $\textbf{r} _s^{(\lambda )}$ $(\langle \textbf{r} \rangle {W_{n, {\textbf {R}}}^{(\lambda )}})$. In this equation, $\mathbbm {n}_{W_{n, {\textbf {R}}}^{(\lambda )}}$ is the occupancy of Wannier center *n* and $\mathbb {J}$ is the number of Wannier centers in structure $\lambda$.

In the ionic polarization, $\textbf{r}_s^{(\lambda )}$ represents the classical position of ion *s* in structure $\lambda$. On the other hand, in the electronic polarization, $\langle \textbf{r} \rangle {W_{n, {\textbf {R}}}^{(\lambda )}}$ signifies the expectation value of the position of the Wannier center *n* in structure $\lambda$. It’s essential to understand that $\langle \textbf{r} \rangle {W{n, {\textbf {R}}}^{(\lambda )}}$ does not correspond to the position of a classical particle and should be interpreted within a quantum mechanical framework.

In order to calculate $\textbf{P}^{(\lambda )}$, we first decompose the electronic polarization based on the number of composite bands. For LiOsO$_3$, there are two distinct composite bands:

- The isolated class of bands II, which includes only deep-lying, fully occupied valence bands.
- The isolated class of bands I, which comprises shallow-lying, fully and partially occupied valence bands, as well as low-lying empty conduction bands.

Thus, we decompose $\textbf{P}_{el}^{(\lambda )}$ into two parts:

6 $$\begin{aligned} \textbf{P}_{el}^{(\lambda )}&= \textbf{P}_{el}^{(\lambda ), (\text {II})} + \textbf{P}_{el}^{(\lambda ), (\text {I}^*)} \nonumber \\&= \frac{e}{{\Omega }^{(\lambda )}} \left[ \sum _{n=1}^{\mathbb {J}^{(\text {II})}} 2 \langle \textbf{r} \rangle _{W_{n, \varvec{R}}^{(\lambda )}} + \sum _{n=1}^{\mathbb {J}^{(\text {I})}} \mathbbm {n}_{W_{n, \varvec{R}}^{(\lambda )}} \langle \textbf{r} \rangle _{W_{n, \varvec{R}}^{(\lambda )}} \right] , \end{aligned}$$

where $\textbf{P}_{el}^{(\lambda ), (\text {II})}$ ($\textbf{P}_{el}^{(\lambda ), (\text {I}^*)}$) is the partial electronic contribution of Wannier centers of class II (I$^*$) into the electronic part of polarization $\textbf{P}_{el}^{(\lambda )}$ at lattice vector $\varvec{R}$ in structure $\lambda$. The factor 2 inside the first summation accounts for fully occupied Wannier centers, whereas $\mathbbm {n}_{W_{n, \varvec{R}}}^{(\lambda )}$ inside the second summation accounts for both fully and partially occupied Wannier centers. We have $\mathbb {J} = \mathbb {J}^{(\text {II})} + \mathbb {J}^{(\text {I})}$. The term $\mathbb {J}^{(\text {I})}$, as the upper limit of the second sum in Eq. (6), refers to the Wannier center of class I. However, the occupation numbers $\mathbbm {n}_{W_{n, \varvec{R}}^{(\lambda )}}$ used in the second term of Eq. (6) are so determined subsequently in step three that the second term itself refers to the polarization of class I$^*$ and yields $\textbf{P}_{el}^{(\lambda ), (\text {I}^*)}$. To complete the calculation of $\textbf{P}^{(\lambda )}$ from Eqs. (5) and (6), we perform the following 5 steps. For a more detailed discussion on each step, readers are referred to Sect. 3.2 of the SMs.

**Figure 3:**
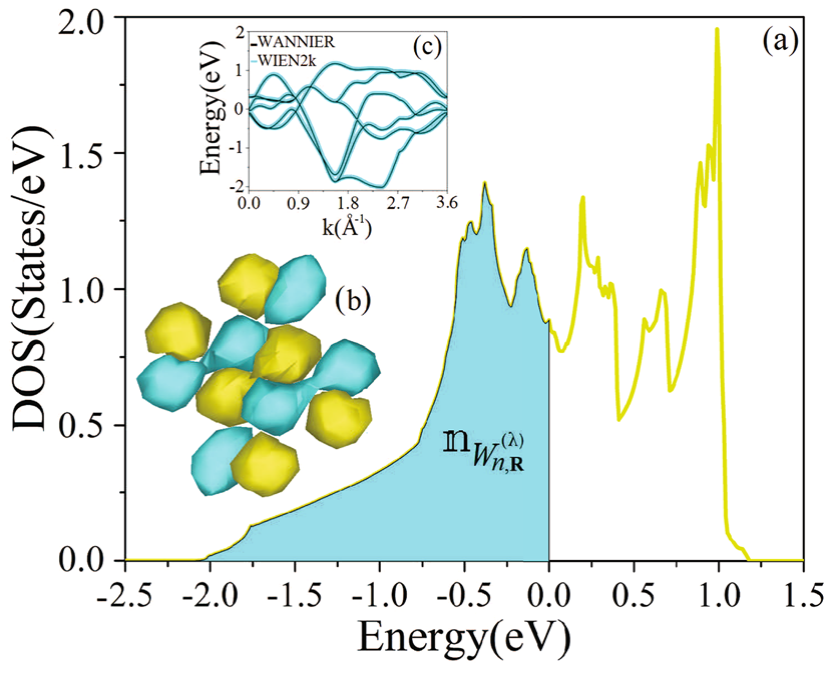
(**a**) DOS projected on one of the 6 Wannier centers related to the energy interval I, as shown in Fig. 1a. The initial DFT calculations were performed by the PBE-GGA DFT for the LiOsO$_3$ in its polar NCS rhombohedral structure. The colored area under the projected DOS up to the Fermi level shows the occupancy of the Wannier center *n* at ${\textbf {R}}$ for the polar NCS rhombohedral structure of the compound, i.e. $\mathbbm {n}_{W_{n, {\textbf {R}}}^{(\lambda )}}$. The area is calculated by taking integration of the projected DOS up to the Fermi level. (**b**) The real-space plot of maximally localized functions, $W_{n, {\textbf {R}}}^{(\lambda )}$, constructed from the Bloch states. (**c**) Original bands were generated directly from the PBE-GGA DFT calculations for the polar NCS phase of the rhombohedral LiOsO$_3$, see thin black bands. Wannier-interpolated bands obtained from the subspace selected by an initially unconstrained projection onto atomic Os:d$_{\text {z}^2}$, Os:d$_{\text {x}^2-\text {y}^2}$, Os:d$_{\text {xy}}$ orbitals for the isolated class of bands I, see thick blue bands. The Fermi level is set to zero in both the DOS and band structure figures.

In the first step, we perform a regular self-consistent DFT calculation for the structure $\lambda$. Then, we restrict the energies to the energy interval II, as shown in Fig. 1. Now, we apply self-consistently the standard maximally localized Wannier functions procedure on the fully occupied valence composite bands of class II for structure $\lambda$, see Fig. 1. This procedure is performed over the Bloch states calculated by WIEN2k package^76,77^ to obtain maximally localized Wannier functions and their centers of charges using Wannier90 code^60–63,65^ and WIEN2WANNIER interface^64^. At this stage, we use the first term of Eq. (5) to calculate $\textbf{P}_{ion}^{(\lambda )}$ entirely while we use the first term of Eq. (6) to calculate partial electronic polarization $\textbf{P}_{el}^{(\lambda ), (\text {II})}$. The latter electronic polarization is partial because it needs to be completed by including contributions of the Wannier centers of class I, i.e. $\textbf{P}_{el}^{(\lambda ), (\text {I})}$ as the second term of Eq. (6).

In the second step, we restrict the energies to the energy interval I, see Fig. 1. Then, we apply self-consistently the generalized maximally localized Wannier functions procedure constructing partly occupied Wannier functions on the composite bands of class I including valence and conduction bands for structure $\lambda$, see Fig. 1. By this way, we calculate the positions of the Wannier centers of the structure $\lambda$ as

7 $$\begin{aligned} \int \textbf{r} |W_{n, {\textbf {R}}}^{(\lambda )}(\textbf{r})|^2 d\textbf{r} = \langle \textbf{r} \rangle {W_{n, {\textbf {R}}}^{(\lambda )}} = (\langle x \rangle {W_{n, {\textbf {R}}}^{(\lambda )}}, \langle y \rangle {W_{n, {\textbf {R}}}^{(\lambda )}}, \langle z \rangle {W_{n, {\textbf {R}}}^{(\lambda )}}), \end{aligned}$$

for Wannier centers $n = 1$ to $\mathbb {J}^{(\text {I})}$. At this stage, we cannot calculate the remaining electronic polarization by the following conventional equation:

8 $$\begin{aligned} \textbf{P}_{el}^{(\lambda )} = \frac{e}{{\Omega }^{(\lambda )}} \sum _{n=1}^{\mathbb {J}} 2 \langle \textbf{r} \rangle {W_{n, {\textbf {R}}}^{(\lambda )}}, \end{aligned}$$

because in this equation the occupation numbers of all the Wannier centers are assumed to be 2, while the class I contains partially occupied Wannier centers. We cannot also use our generalized formula expressed as the second term of Eq. (6), because $\mathbbm {n}_{W_{n, {\textbf {R}}}^{(\lambda )}}$ still are unknown. Therefore, the main task of the next step is to determine the unknown occupation numbers $\mathbbm {n}_{W_{n, {\textbf {R}}}^{(\lambda )}}$ for the Wannier centers of class I.

In the third step, we determine $\mathbbm {n}_{W_{n, {\textbf {R}}}^{(\lambda )}}$ so that the polarization calculated in the following fourth step gives the polarization $\textbf{P}_{el}^{(\lambda ), (\text {I}^*)}$ which is related to the desired class I$^*$. To this end, we first individually project the density of states (DOS) on each of the maximally localized Wannier centers for $n = 1$ to $\mathbb {J}^{(\text {I})}$, see Fig. 3a. For instance, the projected DOS, as shown in Fig. 3a, is obtained by projecting the calculated DOS on one of the maximally localized Wannier centers which is shown in Fig. 3b. Then, we integrate each of the projected DOSs up to the Fermi level. By this, we obtain individually the areas under each of the DOSs projected on the maximally localized Wannier centers up to the Fermi level, e.g. see the filled area under the DOS shown in Fig. 3a which gives the occupancy of the corresponding center of charge. The values of these areas are the desired occupation numbers $\mathbbm {n}_{W_{n, {\textbf {R}}}^{(\lambda )}}$.

In the fourth step, we multiply each of the Wannier centers $\langle \textbf{r} \rangle_{W_{n, {\textbf {R}}}^{(\lambda )}}$ obtained in the second step by their corresponding occupation numbers $\mathbbm {n}_{W_{n, {\textbf {R}}}^{(\lambda )}}$ obtained in the third step. By this way, $\mathbbm {n}_{W_{n, {\textbf {R}}}^{(\lambda )}}\langle \textbf{r} \rangle {W_{n, {\textbf {R}}}^{(\lambda )}}$ are obtained. By substituting $\mathbbm {n}_{W_{n, {\textbf {R}}}^{(\lambda )}}\langle \textbf{r} \rangle {W_{n, {\textbf {R}}}^{(\lambda )}}$ into the second term of Eq. (6), $\textbf{P}_{el}^{(\lambda ), (\text {I}^*)}$ are calculated. Now, by adding $\textbf{P}_{el}^{(\lambda ), (\text {I}^*)}$ calculated in this step to $\textbf{P}_{el}^{(\lambda ), (\text {II})}$ calculated in the first step, we obtain the electronic polarization $\textbf{P}_{el}^{(\lambda )}$ for structure $\lambda$ using the generalized Eq. (6). Let us close this step by indicating a practical note. To this end, let us consider a system whose its polarization direction is pointed along only a single direction. Such a system resemblances the case under study whose polarization direction is oriented along the *c* axis of the hexagonal supercell. In this case, it is enough to consider only $\mathbbm {n}_{W_{n, {\textbf {R}}}^{(\lambda )}}\langle z \rangle {W_{n, {\textbf {R}}}^{(\lambda )}}$ rather than $(\mathbbm {n}_{W_{n, {\textbf {R}}}^{(\lambda )}}\langle x \rangle {W_{n, {\textbf {R}}}^{(\lambda )}}, \mathbbm {n}_{W_{n, {\textbf {R}}}^{(\lambda )}}\langle y \rangle {W_{n, {\textbf {R}}}^{(\lambda )}}, \mathbbm {n}_{W_{n, {\textbf {R}}}^{(\lambda )}}\langle z \rangle {W_{n, {\textbf {R}}}^{(\lambda )}})$.

In the fifth step, based on Eq. (5), we add the ionic part of polarization $\textbf{P}_{ion}^{(\lambda )}$ for structure $\lambda$, as obtained in the first step, to the electronic part of polarization $\textbf{P}_{el}^{(\lambda )}$ for structure $\lambda$, as obtained in the fourth step. By this, we obtain the total electric polarization $\textbf{P}^{(\lambda )}$ for structure $\lambda$.

In analogy to the mBp approach of polarization discussed in “Mean-field-like mBp method of polarization”, all the steps discussed above are similarly performed for structures “$\lambda =0$” and “$\lambda =1$” individually. This leads to the electric polarization vectors $\textbf{P}^{(\lambda =0)}$ for the structure “$\lambda =0$” and $\textbf{P}^{(\lambda =1)}$ for the structure “$\lambda =1$”. Hence, the spontaneous polarization $\Delta \textbf{P}$ can be ultimately calculated as $\Delta \textbf{P}=\textbf{P}^{(\lambda =1)}-\textbf{P}^{(\lambda =0)}$, using the modern theory of polarization^51–59^.

### Merits, limits, and management of mBp and mWf methods of polarization

Here, let us assess the strengths and limitations of our mBp and mWf methods of polarization. While most of computational methods inherently have distinct advantages due to their foundational principles and algorithms, they can also face certain challenges or constraints. Rooted in specific theoretical frameworks, our mBp and mWf methods have been developed to offer particular strengths designed for certain applications. Nevertheless, they are not without limitations. In the subsequent sections, we detail and address these constraints. Our goal is to provide a thorough understanding of these methods’ scope and to highlight situations where they demonstrate optimal effectiveness.

#### mBp method

Our mBp methodology represents a detailed evolution in the filed of polarization calculations. At its core, the mBp approach utilizes mean field-like calculations, with a focused attention on determining the occupation weight, predominantly for ${k}_\perp$. The merit of this approach is most evident when applied to materials like LiOsO$_3$, which exhibit distinct isolated band structures.

One of the notable features of our method is its adaptability to the material’s intrinsic electronic configuration, making it versatile for various materials, including metals and insulators. We observe that the SEPs calculated by mBp (PBE-GGA) and Bp (PBE-GGA+U) for LiOsO$_3$ are close to each other for the material in question, showing the limited impact of bands crossing the Fermi level on the resultant polarization in this specific instance. This observation validates the applicability and reliability of our methodology for systems that exhibit the electronic behavior similar to LiOsO$_3$.

However, it is essential to understand the boundaries of any method’s universality. In scenarios devoid of such isolated band structures, our mBp might need further refinements. One promising direction involves integrating the Wannier bases approach to compute the integral tied to the Berry phase, drawing inspiration from Wang et al.^75^. For cases influenced by d-orbitals around the Fermi level, the Hubbard model, as deduced from our prior research^78^ and validated in this study, emerges as a powerful tool, enabling us to simulate a gap and further analyze the SEP using the conventional mB without modifications.

To address the inherent challenges associated with the mBp method, particularly regarding the use of mean field-like calculations for occupation weight focused on ${k}_\perp$, we incorporated a dense k-mesh in our post-processing calculations. Additionally, by comparing the occupation numbers for class $I^*$ bands—obtained by aggregating weights of ${k}_\perp$—with those from self-consistent DFT calculations, we found noteworthy alignment. This compatibility reaffirms the reliability and soundness of our methodology.

Moreover, by emphasizing the change in polarization between CS and NCS phases, and consistent application of mean field-like calculations to both, we benefit from an inherent error compensation mechanism.

It is worth noting that in the conventional Bp method, the adiabatic condition is crucial for deriving Eq. (10) of the SMs. As our modification builds upon this equation, clarifying the physical rationale behind our adjustments becomes paramount.

Our mBp computational approach is designed to emulate conditions typical of insulating systems. As detailed in step 3 of “Mean-field-like mBp method of polarization” and further expanded in Sect. 3.1 of the SMs, our calculations, particularly when using Eq. (10) (SM), align with the behavior of insulators. This choice ensures adherence to the adiabatic condition inherent in insulating systems.

To enhance the robustness of our method, we have incorporated refinements, such as considering weights at each k-point to adjust the previously calculated phase. These methodological tweaks aim to preserve an adiabatic-like behavior in our calculations, even if the system does not strictly abide by the adiabatic condition.

The underlying physical foundation of these adjustments can be understood as follows: in the adiabatic framework, external parameters like applied electric fields or strain, as the latter used in our case, vary slowly. This variation induces a change in the electronic polarization within a crystal due to adjustments in the self-consistent Kohn-Sham potential. To capture this dynamic, we introduce a $\lambda$ parameterization for the potential, which spans from 0 (initial potential) to 1 (final potential), covering 9 intermediate potential stages, as illustrated in Fig. SM6 of the SMs.

In summation, our mBp methodology, underpinned by rigorous scientific principles and augmented by integrated techniques, is a robust tool in polarization calculations. Its application across a spectrum of materials, judiciously taking into account its foundational strengths and suitable augmentations, promises reliable outcomes.

#### mWf method

Our mWf methodology, designed to address electronic structures like the one observed in our primary case, optimizes the post-processing approach, especially when there are distinct, isolated groups of bands, as visualized in Fig. 1. For such systems, our adaptation of the partially occupied approach by Thygesen et al.^71^ is highly effective, achieving a consistent, reliable representation of electronic behaviors.

However, we acknowledge that in more intricate electronic landscapes, where there is no conspicuous energy window around the Fermi level, challenges can arise. In these instances, our method’s intrinsic flexibility allows the incorporation of refined techniques, like the ’disentangling procedure’ advocated by Souza et al. This procedure ensures that the smallest spread for subsequent Wannier functions is secured, bolstering the accuracy of our calculations.

Moreover, the electronic characteristics of our study material, prominently featuring d-orbitals around the Fermi level (elaborated in Figure SM2 and Sect. 3.2 of the SMs), allow our mWf method to achieve optimal results with minimal iterations. It is paramount to note that while this property aids our specific case, there could be materials where orbital hybridizations are more pronounced. For such cases, our approach remains versatile: by carefully selecting various hybrid orbitals as initial estimates, we ensure the generation of highly localized Wannier functions, even in the face of significant hybridization challenges.

In summary, we have designed our mWf method to be both adaptive and resilient, able to cater to a diverse range of electronic structures while maintaining a high degree of precision. By recognizing potential challenges and proactively integrating solutions into our methodology, we remain confident in the method’s applicability and accuracy across varied electronic landscapes.

## Results and discussions

### SEP direction in LiOsO$_{{3}}$ FE-LM

First, it would be sensible to differentiate the non-polar phase of LiOsO$_3$ FE-LM from its polar phase and subsequently determine qualitatively the direction of the spontaneous electric polarization of the system under consideration. For the sake of conciseness, discussions on the CS R$\bar{3}$c and NCS R3c crystal structures of LiOsO$_3$ are detailed in Sect. 5 of the SMs, as illustrated in Fig. SM5. Notably, the R3c structure can be characterized as the polar phase, denoted by “$\lambda =1$” representing the final structure of the ferroelectric-like phase transition, while the R$\bar{3}$c structure serves as the non-polar phase, indicated by “$\lambda =0$”, representing the initial structure of the transition. Based on the displacement directions identified between the polar and non-polar phases, the spontaneous polarization vector is anticipated to align along the c-axis. An in-depth exploration of this topic is presented in Sect. 3.3 of the SMs, as shown in Fig. SM4. The spontaneous polarization orientation deduced in this section is further corroborated quantitatively in “[Sec Sec11]” and “[Sec Sec12]”, where both the non-polar CS R$\bar{3}$c and polar CS R3c phases are assessed using two distinct methodologies.

### SEP of LiOsO$_{{3}}$: mBp approach of electric polarization

**Table 1:** Calculated partial Berry phases and corresponding polarizations for the non-polar CS R$\bar{3}$c and polar NCS R3c hexagonal supercells of LiOsO$_3$, using the PBE-GGA with mBp and mWf schemes.

	mBp		mWf	
	NCS R3c	CS R$\bar{3}$c	NCS R3c	CS R$\bar{3}$c
$\varphi _{\textit{ion}}$	2.7396	6.2832	–	–
$\varphi _{\textit{el}}^{{\text { (II)}}}$	− 2.2402	0.0000	–	–
$\phi _{\textit{el}}^{(\text {I}^*)}$	0.3267	− 1.5403	–	–
$\phi _{\textit{el}}=\varphi _{\textit{el}}^{{\text { (II)}}} +\phi _{\textit{el}}^{(\text {I}^*)}$	− 1.9135	− 1.5403	–	–
$\phi =\varphi _{\textit{ion}}+2\phi _{\textit{el}}$	− 1.0784	3.2026	–	–
$\textbf{P}_{\textit{ion}}$	0.3130	0.0000	0.3130	0.0000
$\textbf{P}_{\textit{el}}^{\text {(II)}}$	0.2060	0.0000	0.2076	0.0000
$\textbf{P}_{\textit{el}}^{(\text {I}^*)}$	0.0746	− 0.3519	0.0706	− 0.3584
$\textbf{P}_{\textit{el}}=\textbf{P}_{\textit{el}}^{\text {(II)}} +\textbf{P}_{\textit{el}}^{(\text {I}^*)}$	0.2806	− 0.3519	0.2782	− 0.3584
$\textbf{P}=\textbf{P}_{\textit{ion}}+\textbf{P}_{\textit{el}}$	0.5936	− 0.3519	0.5912	− 0.3584
$\Delta {\textbf{P}}=\textbf{P}\text {(R3c)}-\textbf{P}(\text {R}\bar{3}\text {c})$	0.9455		0.9496	

The values for the electronic parts of the Berry phases ($\phi _{\textit{el}}$) and polarizations ($\textbf{P}_{\textit{el}}$) were obtained by adding the contributions from class II ($\varphi _{\textit{el}}^{{\text { (II)}}}$ and $\textbf{P}_{\textit{el}}^{\text {(II)}}$) and class I$^*$ ($\phi _{\textit{el}}^{(\text {I}^*)}$ and $\textbf{P}_{\textit{el}}^{(\text {I}^*)}$). Similarly, total Berry phases ($\phi$) and polarizations ($\textbf{P}$) were calculated by adding ionic and electronic contributions. Spontaneous polarizations ($\Delta {\textbf{P}}$) were derived by subtracting the polarizations of R3c and R$\bar{3}$c structures as $\Delta {\textbf{P}}=\textbf{P}\text {(R3c)}-\textbf{P}(\text {R}\bar{3}\text {c})$. The results are given modulo $e\textbf{R}/\Omega$ and will be unwrapped in “[Sec Sec13]” following the procedure proposed by Resta and Vanderbilt in Ref.^57^. Refer to Sect. 3 of the SMs for more detailed information.

In the modern theory of polarization^51–59^, the spontaneous polarization $\Delta {\textbf{P}}$ is defined as the integrated current flow along the distortion direction during an adiabatic transition from a non-polar CS ($``\lambda =0$”) structure to a polar NCS ($``\lambda =1$”) structure, or in other words, $\Delta {\textbf{P}}:=\textbf{P}^{(\lambda =1)}-\textbf{P}^{(\lambda =0)}$ modulo $e\textbf{R}/\Omega$^52,55–57^, see Sect. 1.1 of SMs.

For the system under consideration, $``\lambda =0$” refers to the R$\bar{3}$c structure, while “$\lambda =1$” corresponds to the R3c. The spontaneous polarization $\Delta {\textbf{P}}=\textbf{P}^{\text {(R3c)}}-\textbf{P}^{\text {(R}\bar{3}\text {c)}}$, which represents the change in polarization during the phase transition from CS to NCS, is a more significant physical quantity than the absolute polarizations of the CS and NCS structures. Despite this, to demonstrate the consistency between the results calculated by our mBp and mWf approaches of polarization, we will present the individual partial polarizations for both phases in addition to $\Delta {\textbf{P}}$.”

Following the first step of the mBp approach, as discussed in “Mean-field-like mBp method of polarization” and Sect. 3.1 of SMs, we have calculated the ionic parts of the polarizations, $\varphi _{ion,\mu }^{\left( \lambda \right) }$, for both the non-polar CS R$\bar{3}$c and polar NCS R3c phases taking the hexagonal supercells containing 30 atoms into account. The results are presented in Table 1. Our numerical results, in agreement with the prediction discussed in “[Sec Sec10]”, confirm that the directions of the polarizations are oriented along the c-axes of the hexagonal supercells. This means that $\varphi _{ion,\mu }^{\left( \lambda \right) }$ for $\mu =1 \& 2$ are almost zeros and thereby negligible compared to $\varphi _{ion,\mu }^{\left( \lambda \right) }$ for $\mu =3$. Hence, the $\mu$ index is known to be 3. Therefore, in Table 1, we omitted the known index $\mu$, simplifying $\varphi _{ion,\mu }^{\left( \lambda \right) }$ to $\varphi _{ion}^{\left( \lambda \right) }$. The non-polar CS R$\bar{3}$c (polar NCS R3c) is the initial (final) phase. The index $\lambda$ for the initial (final) phase known to be 0 (1) refers to R$\bar{3}$c (R3c), viz $\lambda =0\equiv \text {R}\bar{3}\text {c}$ and $\lambda =1\equiv \text {R3c}$. Thus, for simplicity, the $\lambda$ index is also omitted in Table 1, simplifying $\varphi _{ion}^{\left( \lambda \right) }$ to $\varphi _{ion}$.

In the second step of the mBp approach, as discussed in “Mean-field-like mBp method of polarization” and Sect. 3.1 of SMs, we have calculated the electronic part of the Berry phase for all the bands of class II, $\varphi _{el,\mu }^{\left( \lambda \right) , \text {(II)}}$, in both the non-polar CS R$\bar{3}$c and polar NCS R3c phases. In analogy to the simplification made for the ionic part of Berry phase, $\varphi _{el,\mu }^{\left( \lambda \right) , \text {(II)}}$ is also similarly simplified to $\varphi _{el}^{\text {(II)}}$, see Table 1.

Applying the third and fourth steps with Eqs. (38) to (47), as discussed in “Mean-field-like mBp method of polarization” and Sect. 3.1 of SMs, we have calculated the electronic part of the Berry phase for all bands of class I$^*$, $\phi _{el,\mu }^{\left( \lambda \right) , (\text {I}^*)}$, for both the non-polar CS R$\bar{3}$c and polar NCS R3c phases. After omitting $\mu$ and $\lambda$ indexes, as in the previous steps, the results are tabulated as $\phi _{el}^{(\text {I}^*)}$ in Table 1. Please, notice that $\phi$ differs from $\varphi$, see Eq. (38) and the notes after Eq. (39) of SMs, where $\phi$ is defined to be distinguished from $\varphi$.

Following the fifth step, we have first found the total electronic Berry phase, $\phi _{el}$, for both the phases individually by adding $\varphi _{el}^{\text {(II)}}$ to $\phi _{el}^{ (\text {I}^*)}$ as $\phi _{el}=\varphi _{el}^{\text {(II)}}+\phi _{el}^{(\text {I}^*)}$, see Table 1. We have then summed the total electronic Berry phase $\phi _{el}$ and the ionic Berry phase $\varphi _{ion}$ to find the total Berry phase $\phi =2\phi _{el}+\varphi _{ion}$ for both the phases individually, as presented in Table 1, where 2 shows the spin degeneracy.

In the sixth step, the total polarization $\textbf{P}$ could be obtained by substituting the total Berry phase $\phi =2\phi _{el}+\varphi _{ion}$ into Eq. (2) of SMs. However, we here preferred to use the second way indicated in the sixth step to obtain not only the total polarization but also all the partial components of polarization. Therefore, following the second way of the sixth step, we have obtained the ionic polarization $\textbf{P}_{\textit{ion}}$ by substitute the ionic Berry phase $\varphi _{\textit{ion}}$ into Eq. (2) in Sect. 1.1 of SMs for both the phases individually, as tabulated in Table 1. Then, we have obtained the partial electronic polarizations $\textbf{P}_{\textit{el}}^{\text {(II)}}$ and $\textbf{P}_{\textit{el}}^{(\text {I}^*)}$, as well as the total electronic polarization $\textbf{P}_{\textit{el}}$ by substitute the partial electronic Berry phases $\varphi _{\textit{el}}^{{\text { (II)}}}$ and $\phi _{\textit{el}}^{(\text {I}^*)}$, as well as the total electronic Berry phase $\phi _{\textit{el}}$ into Eq. (9) of SMs, receptively, for both the phases individually, as tabulated in Table 1. Consequently, we have obtained the total polarization as $\textbf{P}=\textbf{P}_{\textit{ion}}+\textbf{P}_{\textit{el}}$.

Eventually, using our mBp approach of polarization, we have calculated the spontaneous electric polarization $\Delta {\textbf{P}}(=\textbf{P}\text {(R3c)}-\textbf{P}(\text {R}\bar{3}\text {c}))$ for the FE-LM LiOsO$_3$, as reported in Table 1.

The results show that the ionic Berry phase for the CS (R$\bar{3}$c) structure is $6.2832~\text {rad}$, very close to an integer multiple of $2\pi ~\text {rad}$ (Table 1). Consequently, the ionic part negligibly contributes to the total polarization for the CS phase, as demonstrated by $\textbf{P}_{\textit{ion}}=0.0000$ C/m$^2$ calculated for the R$\bar{3}$c phase.

For the NCS phase, $\varphi _{\textit{ion}}$ is $2.7396~\text {rad}$, a value not equal to an integer multiple of $2\pi ~\text {rad}$. Hence, according to Eq. (2) of SMs, this ionic Berry phase significantly affects the total polarization, leading to $\textbf{P}_{\textit{ion}}=0.3130~\text {C/m}^2$ for the R3c phase.

According to Eqs. (2) and  (9) of SMs, the ionic and electronic polarizations are obtained by multiplying $\frac{\varphi _{\textit{ion}}}{2\pi }$ and $\frac{\phi _{\textit{el}}}{\pi }$ by the quantum of polarization $e\textbf{R}/\Omega$, respectively.

$\varphi _{\textit{el}}^{{\text { (II)}}}$ is − 2.2402 rad for the polar R3c phase and 0.0000 rad for the non-polar R$\bar{3}$c phase (Table 1). It indicates that the partial electronic Berry phase originating from the fully occupied deep-lying bands (class II) does not contribute to the polarization of the R$\bar{3}$c phase, while it significantly contributes to the R3c phase.

Surprisingly, even in the non-polar CS phase, non-zero polarizations exist. For example, the $\phi _{\textit{el}}^{(\text {I}^*)}$ and $\phi _{\textit{el}}(=\phi _{\textit{el}}^{(\text {I}^*)}+\phi _{\textit{el}}^{(\text {II})}=\phi _{\textit{el}}^{(\text {I}^*)}+0.0000=\phi _{\textit{el}}^{(\text {I}^*)})$ are −1.5403 rad for the CS R$\bar{3}$c phase (Table 1), resulting in $\textbf{P}_{\textit{el}}=-0.3519~\text {C/m}^2$.

In *[Sec Sec13]”, we examine the electronic Berry phases of LiNbO$_3$, LiTaO$_3$, BiFeO$_3$, and LiOsO$_3$ and show that our calculated spontaneous polarizations are in agreement with existing experimental data and theoretical results.

However, despite the precise values obtained, we still have to account for the uncertainty rooted in the Berry phase theory of polarization, which defines polarization only modulo a quantum of polarization^57^. This suggests that polarization is a multivalued quantity.

In “[Sec Sec13]”, we address this uncertainty by calculating the polarization at several intermediate points along the transition path following the procedure by Resta and Vanderbilt^57^. This process allows us to select the best branch and to provide unwrapped results of the spontaneous polarization.

### SEP of LiOsO$_{{3}}$: mWf approach of electric polarization

**Table 2:** The *z* components of the Wannier centers, $\langle z \rangle {W_{n, {\textbf {R}}}}$, and occupation numbers, $\mathbbm {n}_{W_{n, {\textbf {R}}}}$, as well as summation of $\mathbbm {n}_{W_{n, {\textbf {R}}}}$ over the Wannier centers, $\sum _{n=1}^{\mathbb {J}^{(\text {I})}=18}{\mathbbm {n}_{W_{n, {\textbf {R}}}}}$, taking the eighteen Wannier centers from $n=1~\text {to}~\mathbb {J}^{(\text {I})}=18$ of class I into account for the NCS R3c (CS R$\bar{3}$c) hexagonal structure of LiOsO$_3$.

n	$\langle z \rangle {W_{n, {\textbf {R}}}}$	$\mathbbm {n}_{W_{n, {\textbf {R}}}}$	n	$\langle z \rangle {W_{n, {\textbf {R}}}}$	$\mathbbm {n}_{W_{n, {\textbf {R}}}}$
1	6.25 (6.63)	1.09 (1.09)	10	12.96 (0.00)	1.09 (1.09)
2	6.25 (6.63)	1.09 (1.09)	11	12.96 (0.00)	1.09 (1.09)
3	6.26 (6.63)	0.82 (0.82)	12	12.96 (0.00)	0.82 (0.82)
4	1.78 (2.21)	1.09 (1.09)	13	8.49 (8.84)	1.09 (1.09)
5	1.78 (2.21)	1.09 (1.09)	14	8.49 (8.84)	1.09 (1.09)
6	1.79 (2.21)	0.82 (0.82)	15	8.49 (8.84)	0.82 (0.82)
7	10.72 (11.05)	1.09 (1.09)	16	4.02 (4.42)	1.09 (1.09)
8	10.72 (11.05)	1.09 (1.09)	17	4.02 (4.42)	1.09 (1.09)
9	10.73 (11.05)	0.82 (0.82)	18	4.02 (4.42)	0.82 (0.82)
$\vdots$	$\vdots$	$\vdots$	$\sum _{n=1}^{18}{\mathbbm {n}_{W_{n, {\textbf {R}}}}}$		18.00(18.00)

As a part of the initial stage of the mWf approach, outlined in “mWf method of polarization” and Sect. 3.2 of the SMs, we focus on the energy interval II. Within this context, we calculate the ionic component of polarization, represented as $\textbf{P}_{ion}^{(\lambda )}$, and the partial electronic polarization, denoted as $\textbf{P}_{el}^{(\lambda ), (\text {II})}$. We carry out these calculations with a focus on two distinct phases: the non-polar centrosymmetric (CS) R$\bar{3}$c phase, and the polar non-centrosymmetric (NCS) R3c phase, taking into account the hexagonal supercells. The results are presented as $\textbf{P}_{ion}$ and $\textbf{P}_{el}^{(\text {II})}$ in Table 1, where the known indexes $\lambda$ are removed for simplicity for both the CS R$\bar{3}$c and NCS R3c phases. Similarly, the indexes $\mu$ have been eliminated because they consistently yield a value of 3 for both the CS R$\bar{3}$c and NCS R3c phases. Our computational findings from the mWf method reveal that the *x* and *y* components of both $\textbf{P}_{ion}^{(\lambda )}$ and $\textbf{P}_{el}^{(\lambda ), (\text {II})}$ are essentially zero, meaning they are remarkably close to integer multiples of $e\textbf{R}/\Omega$. Our results from the mWf method indicate that the *x* and *y* components of $\textbf{P}_{ion}^{(\lambda )}$ and $\textbf{P}_{el}^{(\lambda ), (\text {II})}$ are negligible compared to their respective *z* components in both the centrosymmetric (CS) R$\bar{3}$c and non-centrosymmetric (NCS) R3c hexagonal supercells. This observation aligns with the numerical predictions of the mBp scheme (as discussed in “Mean-field-like mBp method of polarization” and Sect. 3.1 of the SMs) and the theoretical predictions discussed in “[Sec Sec10]”. This evidence substantiates that the polarization vectors $\textbf{P}_{ion}^{(\lambda )}$ and $\textbf{P}_{el}^{(\lambda ), (\text {II})}$ align with the c-axes of the hexagonal supercells in both the CS R$\bar{3}$c and NCS R3c phases. Besides the polarization directions, the computed values of $\textbf{P}_{ion}$ for both the centrosymmetric (CS) R$\bar{3}$c and non-centrosymmetric (NCS) R3c phases, as well as $\textbf{P}_{el}^{(\text {II})}$ for the CS R$\bar{3}$c phase using the mWf method, align perfectly with their respective values calculated by the mBp scheme, as shown in Table 1. Moreover, the mWf-calculated value of the partial electronic polarization $\textbf{P}_{el}^{(\lambda ), (\text {II})}$ for the CS R$\bar{3}$c phase, measured at 0.2076 C/m$^2$, is in close agreement with the mBp-calculated value of 0.2060 C/m$^2$ for the same phase (see Table 1).

Utilizing the second step of the mWf method, as detailed in “mWf method of polarization” and Sect. 3.2 of the SMs, we focus on energy interval I to calculate the positions of the Wannier centers. These positions are determined by evaluating the integral $\int \textbf{r} |W_{n, {\textbf {R}}}^{(\lambda )}(\textbf{r})|^2 d\textbf{r}$, which results in the position vector $\langle \textbf{r} \rangle W{n, {\textbf {R}}}^{(\lambda )} =(\langle x\rangle W{n, {\textbf {R}}}^{(\lambda )} , \langle y \rangle W{n, {\textbf {R}}}^{(\lambda )} , \langle z \rangle W{n, {\textbf {R}}}^{(\lambda )} )$. These calculations are performed for each Wannier center, numbered from $n=1~\text {to}~\mathbb {J}^{(\text {I})}$, while considering both the CS R$\bar{3}$c and NCS R3c phases individually. The number of bands of class I, $\mathbb {M}^{(\text {I})}$, is 18 which equals the number of Wannier centers of class I, i.e. $\mathbb {J}^{(\text {I})}=18$, for the hexagonal supercells of both the CS R$\bar{3}$c and NCS R3c phases. In the FP-LAPW DFT calculations, we have set the separation energy of the valence electrons from core electrons to $-9.0~\text {Ry}$, leading to 306 valence electrons. The number of fully occupied bands of class II is 144. These bands contain $288=144\times 2$ electrons. Thus, the bands of class I contain $18=306-288$ valence electrons. These 18 electrons mostly come form the Os atoms, see Fig.  SM2 of SMs. Although the DOSs shown in Fig.  SM2 of SMs are calculated for the rhombohedral unit cells, the DOSs calculated for the hexagonal supercells, not presented here, show approximately similar behaviors. In the hexagonal supercell, there are six Os$^{5+}$ ions. Each Os$^{5+}$ ion has a nonmagnetic $5\text {d}^3$ ground state, leading to almost 3 valence d-electrons per Os$^{5+}$ ion^17,20,26^. Our results, in agreement with Refs.^17,20,26^, show that the metallic state of LiOsO$_3$ mainly originates from the d-orbital of the Os$^{5+}$ ions, see Fig.  SM2 of SMs. By considering these 3 valence d-electrons, it can be also verified that the total number of valence electrons of class I are approximately $3\times 6=18$. These 18 valence electrons are distributed over the bands of class I, including valence and low-lying conduction bands, so that 6 bands (containing $\approx 12$ electrons) is almost fully occupied, 6 bands (containing $\approx 6$ electrons) is partially occupied, and 6 bands (containing $\approx 0$ electrons) remain almost empty. In fact, these 18 valence electrons are distributed over the bands of class I$^*$, including valence bands only. The polarizations $\textbf{P}_{el}^{(\lambda ), (\text {I}^*)}$ calculated below confirm that the *x* and *y* components of the Wannier centers corresponding to the region I do not contribute to the polarizations in both the CS R$\bar{3}$c and NCS R3c hexagonal supercells. Thus, only the *z* components of the Wannier centers $\langle z \rangle_ {W_{n, {\textbf {R}}}}$ for $n=1~\text {to}~\mathbb {J}^{(\text {I})}=18$ are tabulated here in Table 2 for both the CS R$\bar{3}$c and NCS R3c phases.

Following the third step, we have determined $\mathbbm {n}_{W_{n, {\textbf {R}}}^{(\lambda )}}$ for both the CS R$\bar{3}$c and NCS R3c phases. To do this, we have first projected the total Wannier DOS on each of the 18 maximally localized Wannier centers for $n=1$ to $\mathbb {J}^{(\text {I})}$ individually. Then, we have integrated each of the projected Wannier DOSs up to the Fermi level one by one. By integrating up to the Fermi level, we have changed the working class from the undesired I to the desired I$^*$. The areas under the projected Wannier DOSs calculated up to the Fermi are tabulated in Table 2 as the occupation numbers $\mathbbm {n}_{W_{n, {\textbf {R}}}}$, after removing the known $\lambda$ indexes, for $n=1~\text {to}~\mathbb {J}^{(\text {I})}=18$. We have examined the correctness of the occupation numbers by summing on $\mathbbm {n}_{W_{n, {\textbf {R}}}}$ over all the Wannier centers for both the CS R$\bar{3}$c and NCS R3c phases individually. The examination, as also presented in Table 2, validates that $\sum _{n=1}^{\mathbb {J}^{(\text {I})}=18}{\mathbbm {n}_{W_{n, {\textbf {R}}}}}$ leads to 18.00 for both the CS R$\bar{3}$c and NCS R3c phases individually. It is worth noting that the number of Wannier centers of class I, $\mathbb {J}^{(\text {I})}$, in $\sum _{n=1}^{\mathbb {J}^{(\text {I})}=18}{\mathbbm {n}_{W_{n, {\textbf {R}}}}}=18.00$ equals the number of bands of class I, $\mathbb {M}^{(\text {I})}$, while the resultant value of the summation yields 18.00 which equals the number of valence electrons of class I$^*$. This verifies that the occupation numbers are correctly calculated and the mWf procedure works well so far up to this step. The results show that the occupation numbers of the Wannier centers $\mathbbm {n}_{W_{n, {\textbf {R}}}^{(\lambda )}}$ are almost either $1.09~(:=A)$ or $0.82~(:=B)$ which are close to unity, viz. $A=1.09 \approx B=0.82 \approx 1.00$. This shows that the occupation numbers can be approximately halved by including low-lying empty conduction states besides the fully occupied valence states for constructing the maximally localized Wannier centers. This shows that the 18 electrons are almost uniformly distributed over the 18 centers of the maximally localized Wannier functions constructed from both valence and conduction states. More precisely, by taking the differences between the values of $A=1.09$ and $B=0.82$ into account, a sequence $\underbrace{AAB}_1 \underbrace{AAB}_2 \underbrace{AAB}_3 \underbrace{AAB}_4 \underbrace{AAB}_5 \underbrace{AAB}_6$ with a repeating pattern *AAB* involving 3 elements can be observed which is periodically repeated 6 times for both of the phases. If we multiply the number of elements of the repeating pattern, 3, by the number of repetitions of the pattern, 6, we obtain the number of the 18 centers associated with the maximally localized Wannier functions constructed from both valence and conduction states, viz. $3\times 6=18$. Approximately half of these states, $\approx 9$, belong to the valence region and the other half belong to the conduction states. The element A is repeated twice while the element B is repeated once in the AAB pattern. The amount of the occupation numbers can depend on the number of bands that crosses the Fermi level and the ratio of the number of conduction states added to the total number of valence and conduction states. For the class I, there are 6 bands that cross the Fermi level, and 6 valence bands, as well as 6 conduction bands. The 12 Wannier centers related to the 6 valence bands and the 6 conduction bands are closer to the positions of the Os$^{5+}$ ions while the remaining 6 Wannier centers related to the 6 bands that cross the Fermi level are farther from the positions of the Os$^{5+}$ ions. The occupation number of the 12 Wannier centers which are closer to the ionic positions is larger than that of the remaining 6 Wannier centers which are farther from the ionic positions. The larger (smaller) occupation number is $A=1.09$ ($B=0.82$). This results in the *AAB* pattern.

Following the fourth step, we have determined the partial electronic polarizations $\textbf{P}_{el, \mu }^{(\lambda ), (\text {I}^*)}$ for $\lambda =0~\text {and}~1$ by substituting the multiplications of $\mathbbm {n}_{W_{n, {\textbf {R}}}^{(\lambda )}}\langle \textbf{r} \rangle _{W_{n, {\textbf {R}}}^{(\lambda )}}$ into the second term of Eq. (52) of SMs using the results $\langle \textbf{r} \rangle _{W_{n, {\textbf {R}}}^{(\lambda )}}$ and $\mathbbm {n}_{W_{n, {\textbf {R}}}^{(\lambda )}}$ tabulated in Table 2. The resultant partial electronic polarizations are given for both of the phases in Table 1 as $\textbf{P}_{el}^{(\text {I}^*)}$, where the known indexes $\lambda$ and $\mu$ are removed. We have checked that the *x* and *y* components of this partial electronic polarization are very close to integer multiples of $e\textbf{R}/\Omega$ leading to vanished polarizations along *x* and *y* directions for both the phases individually. The partial polarizations $\textbf{P}_{el}^{(\text {I}^*)}$ are calculated by the mWf method to be 0.0706 C/m$^2$ for the NCS R3c and − 0.3584 C/m$^2$ for the CS R$\bar{3}$c which are close to the corresponding partial polarizations $\textbf{P}_{el}^{(\text {I}^*)}$ calculated by the mBp method, i.e. 0.0746 C/m$^2$ for the NCS R3c and -0.3519 C/m$^2$ for the CS R$\bar{3}$c. This shows that both the mBp and mWf approaches yielding consistent results can be considered as two different reliable methods to predict polarization corresponding to the entangled bands of class I$^*$. Then, we have obtained the electronic polarizations $\textbf{P}_{el}$ for both of the phases using the mWf method expressed in the generalized Eq. (52) of SMs by adding $\textbf{P}_{el}^{(\text {I}^*)}$ to $\textbf{P}_{el}^{ (\text {II})}$, as tabulated in Table 1. The results show that the electronic polarizations $\textbf{P}_{el}$ calculated by the mWf method for both of the phases are in agreement with the corresponding polarizations calculated by mBp method, see Table 1.

Utilizing the fifth step of the mWf method, we have obtained the total electric polarizations $\textbf{P}^{(\lambda )}$ by substituting $\textbf{P}_{ion}^{(\lambda )}$ and $\textbf{P}_{el}^{(\lambda )}$, as tabulated in Table 1, into Eq. (51) of SMs for $\lambda =0~\text {and}~1$. The total electric polarizations calculated by the mWf method are presented for both of the phases in Table 1 as $\textbf{P}$, where the known index $\lambda$ has been removed. The results show that the total electric polarizations calculated by the mWf and mBf methods are consistent with each other, see Table 1. Eventually, we have obtained the spontaneous polarization $\Delta {\textbf {P}}$ by $\Delta \textbf{P}=\textbf{P}^{(\lambda =1)}-\textbf{P}^{(\lambda =0)}$ according to the modern theory of polarization^51–59^. The spontaneous polarization $\Delta {\textbf {P}}$, as calculated by the mWf method, is 0.9496 C/m$^2$ which agrees with the value of 0.9455 C/m$^2$ calculated by the mBp method in “[Sec Sec11]”, see Table 1. This agreement authenticates that mWf and mBp are able to predict consistently spontaneous polarizations of FE-LMs.

Analogous to the spontaneous polarization of 0.9455 C/m$^2$ calculated by the mBp method in “[Sec Sec11]”, the value of 0.9496 C/m$^2$ obtained through the mWf method in this section is not considered the final result due to the quantum uncertainty problem. The phase freedom in the choice of the $u_{n\textbf{k}}$, was shown to leave $\textbf{P}_{el}$, invariant modulo $e\textbf{R}/\Omega$^55^. The quantum uncertainty found in $e\textbf{R}/\Omega$ is reflected by the fact that the Wannier center position is defined only up to a lattice vector^79^. Therefore, the polarization can be considered as a multivalued quantity due to this uncertainty^79^. To overcome the quantum uncertainty problem of the mBp and mWf methods, the main task of the next section is devoted to counting the integer number of quanta involved in the polarizations calculated in “[Sec Sec11]” and/or “[Sec Sec12]”.

In summary, the above discussion covers a multi-step computational method (the mWf approach) that deals with the calculation of ionic and partial electronic polarizations of the non-polar CS R$\bar{3}$c and polar NCS R3c phases in certain hexagonal supercells.

In the first step, our calculations show that the *x* and *y* components of the polarizations are almost zero and therefore negligible in comparison to the *z* components. This implies that the polarization vectors are primarily aligned along the c-axis of the hexagonal supercells. Furthermore, these calculated values agree with prior calculations from the mBp scheme.

The second step involves calculating the positions of the Wannier centers, considering that there are 18 valence electrons predominantly originating from the Os atoms. It confirms that these 18 electrons are evenly distributed over the valence and low-lying conduction bands. Therefore, only the *z* components of the Wannier centers are considered significant and are tabulated.

The third step involves determining the occupation numbers of the Wannier centers by projecting the total Wannier DOS onto each center and then integrating up to the Fermi level. The occupation numbers are nearly equal to one, indicating that the 18 electrons are uniformly distributed over the 18 centers of the maximally localized Wannier functions.

The final step mentioned involves determining the partial electronic polarizations using the calculated occupation numbers and the positions of the Wannier centers from the previous steps.

Consequently, the mWf method accurately calculates the polarizations and verifies the orientation of these polarizations along the c-axis of the hexagonal supercells. It also calculates the positions and occupation numbers of Wannier centers. For more detailed information see Sect. 3.2 of SMs.

### Uniquifying of spontaneous polarization of LiOsO$_{{3}}$ by finding the best branch

In both the Wannier functions and Berry phase approaches of polarization, the spontaneous polarization $\Delta {\textbf{P}}$ along an adiabatic path is a multivalued quantity that can be only well defined modulo a quantum of polarization $e\textbf{R}/\Omega$^57^, where $\textbf{R}$ is the lattice vector in the real space. In principle, there is such an uncertainty in polarization in both the Berry phase approach, as indicated in “Mean-field-like mBp method of polarization” and Sect. 3.1 of SMs, and the Wannier approach, as indicated in “mWf method of polarization” and Sect. 3.2 of SMs. In the Berry phase (Wannier) approach of polarization, a phase (Wannier center position) can be only well-defined modulo $2\pi$ ($\textbf{R}$). This implies that $\Delta {\textbf{P}}$ can be defined uncertainly as $\textbf{P}^{(\lambda =1)}-\textbf{P}^{(\lambda =0)}$ modulo $e\textbf{R}/\Omega$^52,55–57^, which is a consequence of transnational symmetry^80^. The definition “$\Delta {\textbf{P}}:=\textbf{P}^{(\lambda =1)}-\textbf{P}^{(\lambda =0)}~(\text {mod}~e\textbf{R}/\Omega )$” reads “$\Delta {\textbf{P}}$ and $\textbf{P}^{(\lambda =1)}-\textbf{P}^{(\lambda =0)}$ are congruent modulo $e\textbf{R}/\Omega$”. This means that $\Delta {\textbf{P}}$ and $\textbf{P}^{(\lambda =1)}-\textbf{P}^{(\lambda =0)}$ can be different but equivalent in mod $e\textbf{R}/\Omega$ as they have the same remainder when divided by $e\textbf{R}/\Omega$. In this definition, $\Delta {\textbf{P}}$ is a factual quantity that can be observed and measured experimentally while $\textbf{P}^{(\lambda =1)}-\textbf{P}^{(\lambda =0)}$ is a successor quantity proposed by the modern theory of polarization^51–59^ that may not be necessarily equal to the factual quantity. In other words, computing $\textbf{P}^{(\lambda =1)}-\textbf{P}^{(\lambda =0)}$ by the endpoints of the path only, may not always lead to the factual $\Delta {\textbf{P}}$. This is the case because there is no guarantee that the successor spontaneous polarization $\textbf{P}^{(\lambda =1)}-\textbf{P}^{(\lambda =0)}$ is computed using the correct branch. If we only consider the endpoints of the path without verifying the branch’s correctness, we might not obtain the accurate result^57^. Therefore, we have considered the uncertainty problem to uniquely obtain the spontaneous polarization of LiOsO$_3$, as to be discussed subsequently.

Let us first more specifically clarify the problem. For the case under study, both of the polarizations $\textbf{P}^{(\lambda =0)}$ and $\textbf{P}^{(\lambda =1)}$ and consequently the spontaneous polarization $\Delta {\textbf{P}}$ are oriented along the c axes of the hexagonal CS and NCS supercells, see “[Sec Sec10]”, “[Sec Sec11]”, and “[Sec Sec12]”. Therefore, for this case, $\textbf{R}$ employed in $e\textbf{R}/\Omega$ can be simplified as $\textbf{R}=n\text {c} \hat{\textbf{k}}$ so that $|\textbf{R}|=\text {R}=n\text {c}$, where *n* is an integer number and c ($\hat{\textbf{k}}$) is the lattice constant (unit vector) along the Cartesian *z* axis. Hence, the above definition can be represented as $\Delta {\textbf{P}} :=\textbf{P}^{(\lambda =1)}-\textbf{P}^{(\lambda =0)}+ en\text {c}\hat{\textbf{k}}/{\Omega }$ or equivalently as $\Delta {\text {P}}\hat{\textbf{k}} :=\text {P}^{(\lambda =1)}\hat{\textbf{k}}-\text {P}^{(\lambda =0)}\hat{\textbf{k}}+ en\text {c}\hat{\textbf{k}}/{\Omega }$, where $\Delta {\text {P}}=|\Delta {\textbf{P}}|$, $\text {P}^{(\lambda =1)}=|\textbf{P}^{(\lambda =1)}|$, and $\text {P}^{(\lambda =0)}=|\textbf{P}^{(\lambda =0)}|$. By taking a dot product of the latter vector identity with the unit vector $\hat{\textbf{k}}$, it can be simplified to its scalar form $\Delta {\text {P}} :=\text {P}^{(\lambda =1)}-\text {P}^{(\lambda =0)}+ en\text {c}/{\Omega }$. Therefore, the basic task to identify $\Delta {\text {P}}$ uniquely is reduced to determine the integer number *n* for this case with polarization oriented along one-dimension only. We do it below by the procedure proposed in Ref.^57^. To this end, in addition to the starting structure $\lambda =0$” and end structure $\lambda =1$”, as the two endpoints of the adiabatic transition, we have constructed 9 intermediate structures $\lambda =0.1, 0.2, ...,0.9$, as shown in Fig. C2 and discussed in details in Sect. 5.4 of SMs. These intermediate structures are constructed using the freedoms of the structure $\lambda =1$”. The freedoms originate from the 5 internal parameters *z*1, and *z*2, as well as *x*3, *y*3, *z*3 existed in the potions of Li$^{+}$, and Os$^+$, as well as O$^{2-}$ ions in the polar NCS R3c structure^81^, respectively, see Secs. 5.1, 5.2, 5.3 and 5.4 of SMs. It is well-known that If $|\Delta {\textbf{P}}| \ll |e\textbf{R}/\Omega |$, the uncertainty may not be a serious problem^82,83^. This condition, however, is not generally satisfied by all the compounds such as LiOsO$_3$. Therefore, in Sect. 5.4 of SMs, we have forced the transition to occur slowly from the starting structure $\lambda =0$” to the end structure $\lambda =1$” through the intermediate structures $\lambda =0.1, 0.2, ...,0.9$. To this end, we have constructed the first intermediate structure $\lambda =0.1$” to be very close to the starting structure “$\lambda =0$”, as discussed in detail in Sect. 5.4 of SMs. By comparing structures “$\lambda =0$” and $``\lambda =0.1$”, we have introduced some atomic vector steps $\varvec{\lambda _{0.1}}$ and distorted the structures one by one to gradually and slowly arrive at the endpoint $``\lambda =1$” step by step, see Sect. 5.4 of SMs. In this way, we find a chance to identify a sudden change (jump), if any, in the calculated polarization at an intermediate distorted structure compared to its previous and next structures. If a jump (ascent or descent) occurs, we modify it to make smooth the path by shifting the jumped polarization, i.e. pulling downward the ascent polarization or pushing upwards the descent polarization, using a negative or positive integer multiple of the quantum of polarization, as practically discussed below. In fact, by this way, we unwrap the polarizations (Berry phases) of the constructed structures step by step which are by default traditionally wrapped into the interval $[-e\textbf{R}/2\Omega , e\textbf{R}/2\Omega ]\equiv [-en\text {c}/{2\Omega }, en\text {c}/{2\Omega }]$ ($[-\pi , \pi ]$). Unwrapping refers to adjusting the phases of a signal to allow for smooth transitions. When phase jumps between successive signals are greater than or equal to the difference of $\pi$, unwrapping the phase helps in achieving continuous signals.

**Figure 4:**
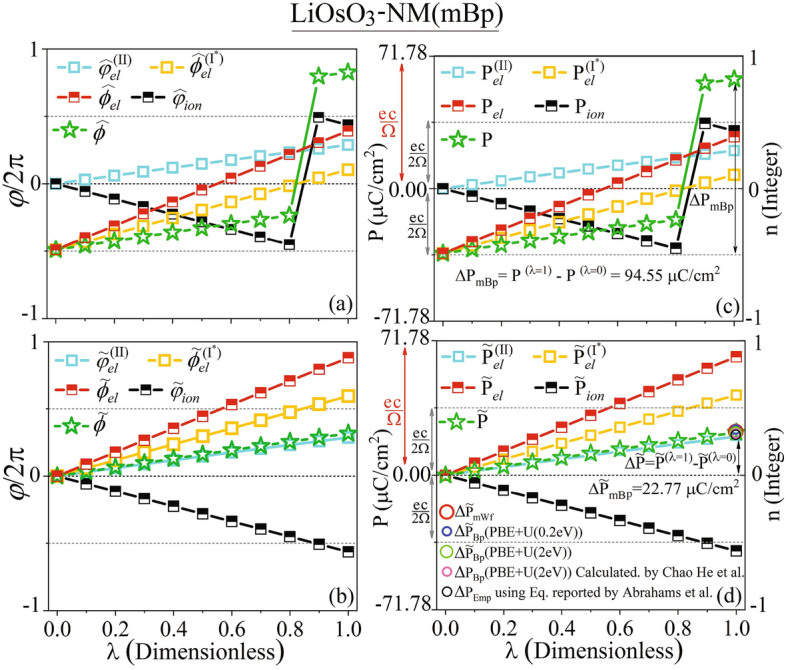
(**a**) Total, and wrapped partial Berry phases versus $\lambda$. (**b**) Unwrapped total, and partial Berry phases versus $\lambda$. All the Berry phases are scaled by $2\pi$ in (**a**,**b**) so that the interval of wrapping is simplified from $[-\pi , \pi ]$ to [− 0.5, 0.5] in (**a**). (**c**) Total, wrapped partial, and corresponding spontaneous polarizations versus $\lambda$. The partial polarizations are wrapped into [$-e\text {c}/{2\Omega }, e\text {c}/{2\Omega }$], where $e\text {c}/\Omega =71.78\,\upmu {\text {C}}/\text {cm}^2$ is the quantum of polarization. (**d**) Unwrapped total, partial, and corresponding spontaneous polarizations versus $\lambda$. The unit of polarizations is $\upmu {\text {C}}/\text {cm}^2$ in (**c**,**d**). The auxiliary symbols $\wedge$ and $\sim$ indicate that when wrapping and/or shifting are/is performed, if necessary, compared to the results presented in Table 1, see “[Sec Sec13]” where the symbols are defined. All the Berry phases and as a result polarizations are calculated by the mBp scheme including non-spin-polarized PBE-GGA along the distortion path as functions of structure $\lambda$ from $``\lambda =0$” to $``\lambda =1$” by step 0.1. The quantum of polarization and its number *n* are shown in (**c**,**d**). Our SEPs calculated by mWf, PBE-GGA+U with $\text {U}=0.2$ and 2 eV are presented for comparison. The mBp, mWf, and empirical results presented in this figure are obtained for the metallic state of the NM LiOsO$_3$. Our GGA+U results and the GGA+U result taken from Ref.^22^ are calculated for the nonmetallic state of the G-AFM LiOsO$_3$ using the standard Berry phase method. The P$_\text {Emp}$ is extracted from Ref.^67^, generated using an empirical equation. The theoretical datum is taken from Ref.^22^.

In addition to the components of the Berry phases of the structures $``\lambda =0$” and $``\lambda =1$”, tabulated in Table 1, here, we have also recalculated all these components for the 9 intermediate $``\lambda = 0.1, 0.2, ..., 0.9$” structures of the non-magnetic (NM) metal LiOsO$_3$, shown in Fig. C2 of SMs. To this end, a set of non-spin-polarized PBE-GGA calculations are performed self-consistently by the mBp method, discussed in “Mean-field-like mBp method of polarization” and Secs. 1.1 and 3.1 of SMs. The Berry phases of these 9 intermediate structures together with those of the starting structure $``\lambda =0$” and the end structure $``\lambda =1$” are all represented as $\widehat{\varphi } _{\textit{ion}}$, $\widehat{\varphi } _{\textit{el}}^{{\text { (II)}}}$, $\widehat{\phi } _{\textit{el}}^{(\text {I}^*)}$, $\widehat{\phi } _{\textit{el}}$, $\widehat{\phi }$ in Fig. 4a. Here, the hat symbol over the ionic components indicates that if the ionic Berry phases are out of the range $[-\pi , \pi ]$, they are first wrapped into the interval $[-\pi , \pi ]$, and then they are divided by $2\pi$. This can be represented as $\widehat{\varphi } _{\textit{ion}}=(\varphi _{\textit{ion}}\pm 2\pi )/2\pi$. In this case, the plus (minus) sign is used when $\varphi _{\textit{ion}}\leqslant -2\pi$ ($\varphi _{\textit{ion}}\geqslant 2\pi$). We can verify the consistency at the initial point $``\lambda =0$” by considering $\varphi _{\textit{ion}}=6.2832$ Rad, which is reported in Table 1. In verifying this, we find that the results presented in Fig. 4a and Table 1 are consistent with each other at $``\lambda =0$”, *viz.* since $\varphi _{\textit{ion}}=6.2832$ satisfies the condition $\varphi _{\textit{ion}}\geqslant 2\pi$, then $0=\widehat{\varphi } _{\textit{ion}}=(\varphi _{\textit{ion}}- 2\pi )/2\pi =(6.2832- 2\pi )/2\pi \approx 0/2\pi =0$. If the ionic Berry phases belong to the range $[-\pi , \pi ]$, they are divided by $2\pi$ only, represented as $\widehat{\varphi } _{\textit{ion}}=\varphi _{\textit{ion}}/2\pi$. We can verify this with the example $0.4360=\widehat{\varphi } _{\textit{ion}}=\varphi _{\textit{ion}}/2\pi =2.7396/2\pi \approx 0.4360$ at $``\lambda =1$”, where $\varphi _{\textit{ion}}=2.7396 \in [-\pi , \pi ]$ as tabulated in Table 1. For the electronic parts of the Berry phases, the hat symbol on the electronic components indicates that the electronic Berry phases are multiplied by 2, and if (after multiplying by 2) they lie out of the range $[-\pi , \pi ]$, then they are wrapped into the interval $[-\pi , \pi ]$, and finally they are divided by $2\pi$, e.g. $\widehat{\varphi } _{\textit{el}}^{{\text { (II)}}}=(2\varphi _{\textit{el}}^{{\text { (II)}}}\pm 2\pi )/2\pi$, where the plus (minus) sign stands for the case $2\varphi _{\textit{el}}^{{\text { (II)}}} \leqslant -2\pi$ ($2\varphi _{\textit{el}}^{{\text { (II)}}}\geqslant 2\pi$). At $``\lambda =1$”, from Table 1 we have $\varphi _{\textit{el}}^{{\text { (II)}}}=-2.2402$ Rad and from Fig. 4a we have $\widehat{\varphi } _{\textit{el}}^{{\text { (II)}}}=0.2869$, which can be consistently converted to $\varphi _{\textit{el}}^{{\text { (II)}}}$ as $0.2869=\widehat{\varphi } _{\textit{el}}^{{\text { (II)}}}=(2(-2.2402)+ 2\pi )/2\pi \approx 0.2869$, where $\widehat{\varphi } _{\textit{el}}^{{\text { (II)}}}=(2\varphi _{\textit{el}}^{{\text { (II)}}}+ 2\pi )/2\pi$ is used, since $2\varphi _{\textit{el}}^{{\text { (II)}}}=2(-2.2402)=-4.4804\leqslant -2\pi$. If after multiplying the electronic Berry phases by 2 they are in the range $[-\pi , \pi ]$, then they are divided by $2\pi$ only, e.g. $\widehat{\varphi } _{\textit{el}}^{{\text { (II)}}}=2\varphi _{\textit{el}}^{{\text { (II)}}}/2\pi$. At $``\lambda =0$”, from Table 1 and Fig. 4a, it can be seen that $\varphi _{\textit{el}}^{{\text { (II)}}}=\widehat{\varphi } _{\textit{el}}^{{\text { (II)}}}=0$, which are converted as $0=\widehat{\varphi } _{\textit{el}}^{{\text { (II)}}}=(2\times 0)/2\pi =0$, where $\widehat{\varphi } _{\textit{el}}^{{\text { (II)}}}=2\varphi _{\textit{el}}^{{\text { (II)}}}/2\pi$ is used, since $2\varphi _{\textit{el}}^{{\text { (II)}}}=2\times 0=0\in [-\pi , \pi ]$. In analogous to $\widehat{\varphi } _{\textit{el}}^{{\text { (II)}}}$, the same conversion relations can be applied on $\widehat{\phi } _{\textit{el}}^{(\text {I}^*)}$. At $``\lambda =0$”, from Fig. 4a and Table 1 it can be seen that $-0.4903=\widehat{\varphi } _{\textit{el}}^{(\text {I}^*)}=(2\varphi _{\textit{el}}^{(\text {I}^*)})/2\pi =(2(-1.5403))/2\pi =-0.4903$, where the condition $2\varphi _{\textit{el}}^{(\text {I}^*)}=2(-1.5403)=-3.0806\in [-\pi , \pi ]$ is satisfied. At $``\lambda =1$”, we have $0.1040=\widehat{\varphi } _{\textit{el}}^{(\text {I}^*)}=(2\varphi _{\textit{el}}^{(\text {I}^*)})/2\pi =(2\times 0.3267)/2\pi =0.1040$, where the condition $2\varphi _{\textit{el}}^{(\text {I}^*)}=2\times 0.3267=0.6534\in [-\pi , \pi ]$ is satisfied, see Fig. 4a and Table 1. In Fig. 4a, $\widehat{\phi } _{\textit{el}}$ is obtained by the summation of $\widehat{\varphi } _{\textit{el}}^{{\text { (II)}}}$ and $\widehat{\phi } _{\textit{el}}^{(\text {I}^*)}$, i.e. $\widehat{\phi } _{\textit{el}}= \widehat{\varphi } _{\textit{el}}^{{\text { (II)}}}+\widehat{\phi } _{\textit{el}}^{(\text {I}^*)}$. We verify from Fig. 4a that $\widehat{\phi } _{\textit{el}}= \widehat{\varphi } _{\textit{el}}^{{\text { (II)}}}+\widehat{\phi } _{\textit{el}}^{(\text {I}^*)}=0+(-0.4903)=-0.4903$ at $``\lambda =0$” and $\widehat{\phi } _{\textit{el}}= \widehat{\varphi } _{\textit{el}}^{{\text { (II)}}}+\widehat{\phi } _{\textit{el}}^{(\text {I}^*)}=0.2869+0.1040=0.3909$ at $``\lambda =1$”. In Fig. 4a, $\widehat{\phi }$ is obtained as $\widehat{\phi }=\widehat{\varphi } _{\textit{ion}}+\widehat{\phi } _{\textit{el}}$. It can be also verified from Fig. 4a as $\widehat{\phi }=\widehat{\varphi } _{\textit{ion}}+\widehat{\phi } _{\textit{el}}=0+(-0.4903)=-0.4903$ at $``\lambda =0$” and $\widehat{\phi }=\widehat{\varphi } _{\textit{ion}}+\widehat{\phi } _{\textit{el}}=0.4360+0.3909=0.8269$ at $``\lambda =1$”.

The results show that the electronic components $\widehat{\varphi } _{\textit{el}}^{{\text { (II)}}}$, $\widehat{\phi } _{\textit{el}}^{(\text {I}^*)}$, $\widehat{\phi } _{\textit{el}}$ are strictly and smoothly increasing functions of the distortion parameter $\lambda$, see Fig. 4a. Without loss of generality, for convenience only, we displace the origins of $\widehat{\phi } _{\textit{el}}^{(\text {I}^*)}$ and $\widehat{\phi } _{\textit{el}}$ and thereby entirely shift them so that they start from zero. These shifts by constant values do not change the results, because the spontaneous polarization as the final important physical quantity is obtained from the difference between the polarizations calculated at the starting and end structures, $\Delta {\text {P}} = \text {P}^{(\lambda =1)}-\text {P}^{(\lambda =0)}$, so that any constant shifts are canceled out. The shifted $\widehat{\phi } _{\textit{el}}^{(\text {I}^*)}$ and $\widehat{\phi } _{\textit{el}}$ are shown as $\widetilde{\phi } _{\textit{el}}^{(\text {I}^*)}$ and $\widetilde{\phi } _{\textit{el}}$ in Fig. 4b.

As the parameter $\lambda$ increases from 0 to 0.8, the ionic component $\widehat{\varphi } _{\textit{ion}}$ decreases from zero to near the lower limit of the border shown by a horizontal dashed line at $\widehat{\varphi }=-0.5$, or equivalently at $\varphi =2\pi \widehat{\varphi }=2\pi (-0.5)=-\pi$, see Fig. 4a. Then, $\widehat{\varphi } _{\textit{ion}}$ at $``\lambda =0.9$” suddenly jumps to near the upper border indicated by a horizontal dashed line at $\widehat{\varphi }=0.5$, or equivalently at $\varphi =2\pi \widehat{\varphi }=2\pi (0.5)=\pi$, see Fig. 4a. Like before $``\lambda =0.9$”, again $\widehat{\varphi } _{\textit{ion}}$ continues to decrease from $``\lambda =0.9$” to $``\lambda =1$”, see Fig. 4a.

The jump in $\widehat{\varphi } _{\textit{ion}}$ detected at $``\lambda =0.8$” is not physically meaningful. By increasing the distortions very slowly, it may be expected to observe a smooth evolution between sequential structures leading to a non-zigzag path. Therefore, we unwrap, as shown in Fig. 4b, the sudden jump by pulling downwards the ascent $\widehat{\varphi } _{\textit{ion}}$ at $``\lambda =0.9$” to $\widetilde{\varphi } _{\textit{ion}}$ which is performed by subtracting a quantum of Berry phase divided by $2\pi$ from $\widehat{\varphi } _{\textit{ion}}$, viz. $\widehat{\varphi } _{\textit{ion}} \xrightarrow {\text {unwraps to}} \widetilde{\varphi } _{\textit{ion}}=\widehat{\varphi } _{\textit{ion}}-2\pi /2\pi =\widehat{\varphi } _{\textit{ion}}-1$.

Similarly, we unwrap $\widehat{\varphi } _{\textit{ion}}$ at $``\lambda =1$” to smooth the evaluation of ionic path from $``\lambda =0.8$” to $``\lambda =1$”, see Fig. 4b. By unwrapping $\widehat{\varphi } _{\textit{ion}}$ at $``\lambda =0.9$” and $``\lambda =1$”, the entire ionic path becomes smooth and strictly decreasing, eliminating the previously observable jump in $\widehat{\varphi } _{\textit{ion}}$ through all the intermediate successive structures from $``\lambda =0$” to $``\lambda =1$”. The unwrapped $\widehat{\varphi } _{\textit{ion}}$ is represented as $\widetilde{\varphi } _{\textit{ion}}$ in Fig. 4b.

The $\widehat{\varphi } _{\textit{el}}^{{\text { (II)}}}$ remains unchanged, since $\widehat{\varphi } _{\textit{el}}^{{\text { (II)}}}$ needs to be neither unwrapped nor shifted. It is also represented as $\widetilde{\varphi } _{\textit{el}}^{{\text { (II)}}}$ in Fig. 4b, keeping in mind that $\widetilde{\varphi } _{\textit{el}}^{{\text { (II)}}}=\widehat{\varphi } _{\textit{el}}^{{\text { (II)}}}$. Although $\widehat{\phi }$ can be first unwrapped similar to $\widehat{\varphi } _{\textit{ion}}\rightarrow \widehat{\varphi } _{\textit{ion}}$ and then shifted similar to $\widehat{\phi } _{\textit{el}} \rightarrow \widetilde{\phi } _{\textit{el}}$, we obtain and represent it as $\widetilde{\phi }$ in Fig. 4b more simply by the summation of the shifted $\widetilde{\phi } _{\textit{el}}$ and wrapped $\widetilde{\varphi } _{\textit{ion}}$ as $\widetilde{\phi }=\widetilde{\varphi } _{\textit{ion}}+\widetilde{\phi } _{\textit{el}}$. The unwrapping and shifting procedure depicted in Fig. 4b yields smooth evaluations of the Berry phases across all structures—initial, intermediate, and final.

Analogous to the Berry phases shown in Fig. 4a, partial electronic and ionic components of the polarizations calculated in $\mu$C/cm$^2$ for the 11 structures $``\lambda = 0, 0.1, 0.2,..., 0.9, 1$” are shown in Fig. 4c. The polarizations of the initial and final structures $``\lambda =0$” and $``\lambda =1$”, as the endpoints of the paths, are identical to the polarizations calculated in “Mean-field-like mBp method of polarization” and Sect. 3.1 of SMs, compare Table 1 with the endpoints of the paths shown in Fig. 4c, taking the conversion relation $``100\,{\upmu } {\text {C}/\text {cm}^{2}}=1~{{\text {C}/\text {m}}^{2}}$” into account. As expected from Eqs. (3) and (9) of SMs, the polarizations vary the same as Berry phases with respect to $\lambda$, compare Fig. 4a,c. Based on the wrapped results presented in Fig. 4c, the spontaneous polarization is calculated to be $94.55\,\upmu \text {C}/\text {cm}^2$, which is consistent with the results presented in Table 1 as $0.9455~\text {C}/\text {m}^2$, viz. $\Delta \text {P}=\text {P}^{(\lambda =1)}-\text {P}^{(\lambda =0)}=94.55\,\upmu \text {C}/\text {cm}^2=0.9455~\text {C}/\text {m}^2$. In order uniquely determine $\Delta \text {P}$ and make certain the latter result, we determine the integer *n* by unwrapping the polarizations presented in Fig. 4c. To this end, the polarizations are unwrapped and the results are shown in Fig. 4d, where the tilde symbol in this figure denotes any necessary unwrapping and/or shifting tasks. The transformation procedure of the polarizations, including both unwrapping and shifting operations, from Fig. 4c to d is similar to that of the Berry phases from Fig. 4a to b, as discussed above. The variations of the unwrapped polarizations with respect to $\lambda$ also behave like the unwrapped Berry phases, compare Fig. 4b,d and see the proportionality relations between polarizations and Berry phases in Eqs. (3) and (9) of SMs. The integer *n* is indicated by the vertical axes on the right of the Fig. 4c,d. By comparing Fig. 4c with Fig. 4d, the integer number *n* is determined to be $-1$ for the FE-LM LiOsO$_3$. Therefore, by recalling that $\Delta {\text {P}} :=\text {P}^{(\lambda =1)}-\text {P}^{(\lambda =0)}+ en\text {c}/{\Omega }$ and noting that $e\text {c}/{\Omega }=71.78\,\upmu \text {C}/\text {cm}^2$, ultimately we uniquely determined the spontaneous polarization of the FE-LM LiOsO$_3$ as $\Delta \widetilde{\text {P}}=\widetilde{\text {P}}^{(\lambda =1)}-\widetilde{\text {P}}^{(\lambda =0)} =\text {P}^{(\lambda =1)}-\text {P}^{(\lambda =0)}+en\text {c}/{\Omega }= 94.55\,\upmu \text {C}/\text {cm}^2+ (-1)\times 71.78\,\upmu \text {C}/\text {cm}^2=22.77 \,\upmu \text {C}/\text {cm}^2$.

The auxiliary symbols $\wedge$ and $\sim$ are used only temporarily in this subsection for clarity. They indicate where wrapping and/or shifting operations are performed. It is important to note that wrapping and shifting are practical operations. Their sole purpose is to ascertain the final, factual spontaneous polarization. Spontaneous polarization is a physical quantity that can be observed in nature and measured experimentally. The theoretical calculation of spontaneous polarization may depend on these practical operations. However, the experimentally measured spontaneous polarization, as observed in nature, is evidently independent of these operations. Given this, we have chosen not to use the $\wedge$ and $\sim$ symbols in other sections and subsections of this manuscript. We have simply reported the results without any additional symbols, such as $\text {P}$, $\Delta \text {P}$, and so on. This is under the understanding that the aforementioned operations are applied as necessary.

## Validity of the SEP predicted for FE-LM LiOsO$_{{\textbf {3}}}$

**Figure 5:**
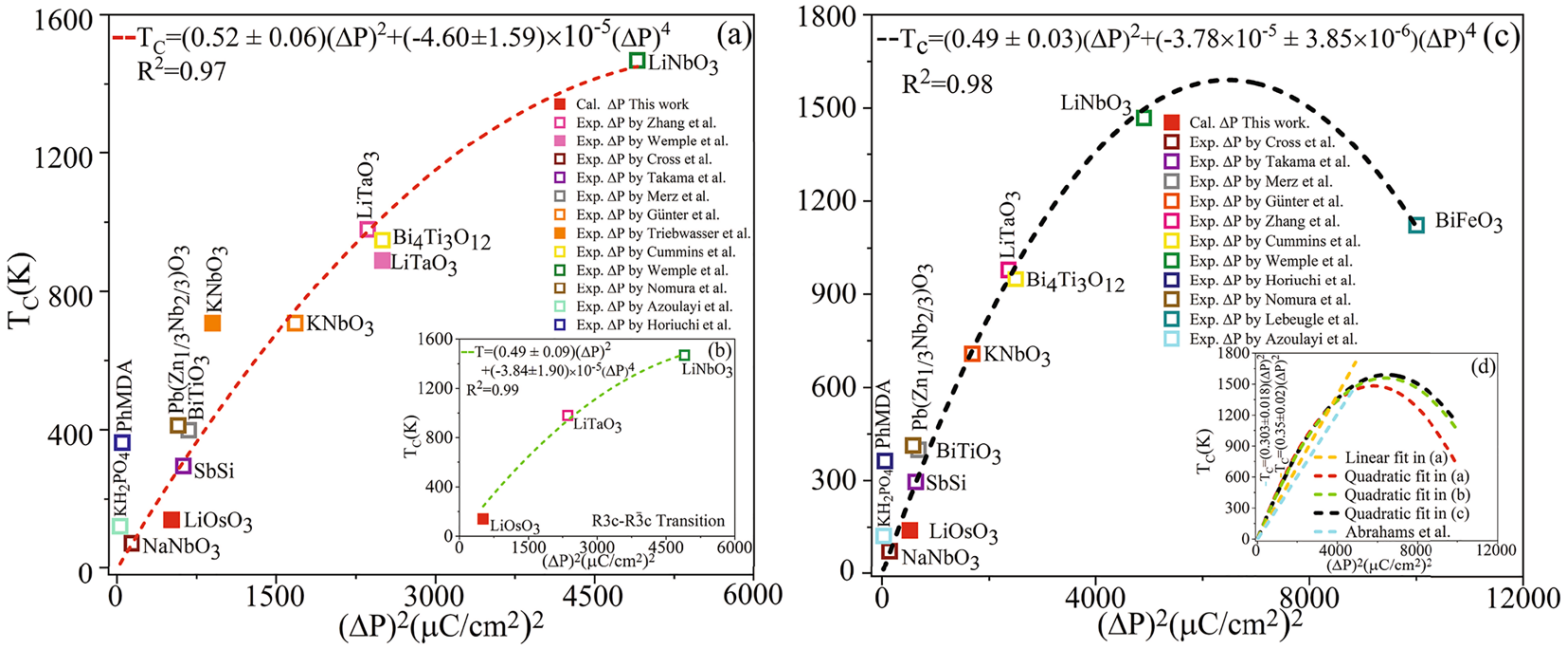
Empirical T$_c-(\Delta \text {P})^2$ dependence for (**a**) 10 normal ferroelectrics, indicated by empty square symbols, (**b**) 2 normal Li-based ferroelectrics, undergoing the same R3c to R$\bar{3}$c phase transition as the system under study, (**c**) 11 normal ferroelectrics, including the 10 compounds considered in (**a**) plus the multiferroic BiFeO$_3$. (**d**) The linear empirical Eq. (12), proposed by Abrahams et al.^67^, and our empirical quadratic Eqs. (17)–(19), as well as linear Eq. (20), emerged from experimental data^68,85,87,89,92–101^. The experimental T$_c$ of the FE-LM LiOsO$_3$ is taken from Ref.^15^ and its $\Delta \text {P}$ is calculated in the present work by our proposed mBp method.

In *[Sec Sec13]”, we predicted the SEP of ferroelectric lithium osmate (LiOsO$_3$) to be approximately $22.77\,\upmu \text {C}/\text {cm}^2$. This value aligns closely with the established SEP of Barium Titanate (BaTiO$_3$), reported to be $25\,\upmu \text {C}/\text {cm}^2$^67,68^, as can be observed in Fig. 5a,c. This parallel is further corroborated by the research conducted by Zabalo et al.^30^, where similar critical bending radii were noted for these two compounds. Now, we aim to further validate and verify the accuracy of our prediction, aligning with predictions made by previous researchers as thoroughly reviewed in the introduction section.

### Numerical verification: consistency of mBp and mWf

In “[Sec Sec12]”, the SEP of theFE-LM LiOsO$_3$ was numerically calculated to be $0.9496\text {C}/\text {m}^2$ using the mWf method, as seen in Table 1. This value is known to suffer from the uncertainty problem, arising from the fact that a Wannier center is well-defined modulo $\textbf{R}$, as discussed in “[Sec Sec13]”. Analogously to the unwrapping procedure elaborated in detail in “[Sec Sec13]” for the mBp method, we similarly found that $n=-1$ for the mWf method. This leads to $\Delta \widetilde{\text {P}}=\widetilde{\text {P}}^{(\lambda =1)}-\widetilde{\text {P}}^{(\lambda =0)} =\text {P}^{(\lambda =1)}-\text {P}^{(\lambda =0)}+en\text {c}/{\Omega }= 94.96\,\upmu \text {C}/\text {cm}^2+ (-1)\times 71.78\,\upmu \text {C}/\text {cm}^2=23.18\,\upmu \text {C}/\text {cm}^2$ for the SEP of the FE-LM LiOsO$_3$ by the mWf method, as shown in Fig. 4d and Table 3. By comparing the $23.18\,\upmu \text {C}/\text {cm}^2$ predicted by the mWf method with the $22.77\,\upmu \text {C}/\text {cm}^2$ predicted by the mBp method, we can at least conclude that the numerical mBp and mWf methods of calculating polarization consistently yield similar results for the SEP in this ferroelectric metal, as shown in Fig. 4d and Table 3. Furthermore, since the numerical mBp and mWf methods of calculating polarization, as discussed in “Mean-field-like mBp method of polarization”, “mWf method of polarization” as well as Secs.3.1 and  3.2 of the SMs, are different, it is highly unlikely that their similar results were merely coincidental. However, in the following sections, we will provide further evidence to suggest that these results are also likely to be close to the experimental value.

### Empirical verification: quadratic order

**Table 3:** $\Delta {\text {P}}$ calculated for LiOsO$_3$, LiNbO$_3$, LiTaO$_3$ and BiFeO$_3$, together with the available experimental, empirical, and theoretical results.

Crystal	Scheme	XC	U(eV)	SP	Order	Metal	Code	T$_c$(K)	*a* (Å)	R (Å)	$\Omega ($Å$^3$)	$\frac{e\text {R}}{\Omega }\,(\upmu {\text {C}}/\text {cm}^2)$	$\Delta {\text {P}} (\upmu {\text {C}}/\text {cm}^2)$	Ref.
LiOsO$_3$	mBp	PBE		No	NM	Yes	WIEN2k		5.077	13.412	299.359	71.78	22.77	*
	mWf	PBE		No	NM	Yes	WIEN2k		5.077	13.412	299.359	71.78	23.18	*
	Emp.			No	NM	Yes		140					21.50±0.64$^a$	*
	Bp	PBE+U	0.2	Yes	G-AFM	No	WIEN2k		5.077	13.412	299.359	71.78	24.33	*
		PBE+U	2.0	Yes	G-AFM	No	WIEN2k		5.077	13.412	299.359	71.78	22.32	*
		LDA+U	2.0	Yes	G-AFM	No	VASP			13.210$^b$	97.800$^b$	216.4$^b$	22.23	^22^
		LDA		Yes	G-AFM	No	VASP						23.46$^c$	^27^
	Exp.			No	NM	Yes		140	5.046	13.239	291.931	72.66		^15^
BiFeO$_3$	Bp	PBE+U	4.0	Yes	G-AFM	No	WIEN2k		5.579	13.870	373.857	59.44	101.90$^d$	^79^
	Bp	PBE+U	2.0	Yes	G-AFM	No	WIEN2k		5.579	13.870	373.857	59.44	103.52$^d$	^79^
		PBE+U	2.0	Yes	G-AFM	No	VASP		5.623	14.058	384.924	58.51	100.30$^e$	^85^
		LDA+U	2.0	Yes	G-AFM	No	VASP		5.497	13.484	352.862	61.22	94.80$^e$	^85^
	Exp.			Yes	G-AFM	No		1143		13.870	373.857	59.44	100.00	^86^
LiNbO$_3$	Bp	PBE		No	NM	No	WIEN2k		5.148	13.863	318.212	69.80	78.31	*
		PBE		No	NM	No	VASP		5.203	14.111	330.812	68.34	84.40	^85^
		LDA		No	NM	No	VASP		5.093	13.807	310.157	71.32	77.90	^85^
		LDA		No	NM	No	ABINIT		5.151	13.703	314.869	69.73	86.00	^87^
	Exp.			No	NM	No		1468$^f$	5.148$^g$	13.863$^g$	318.212$^g$	69.80$^g$	71.00	^88^
LiTaO$_3$	Bp	LDA		No	NM	No	WIEN2k		5.148	13.767	315.970	69.80	53.12	*
	VB			No	NM	No							53.00	^89^
	Exp.			No	NM	No		891$^c$	5.148$^h$	13.767$^h$	315.970$^h$	69.80$^h$	50.00	^88^
	Exp.			No	NM	No		980	5.144	13.781	315.760	69.93	48.60	^90^

R is the magnitude of the real-space lattice vector along the polarization direction, i.e. R = c, where c is the lattice constant along the Cartesian *z*-axis. *a* is the other hexagonal lattice parameter, $\Omega$ is the volume of the unit cell, $\frac{e\text {R}}{\Omega }$ is the quantum of polarization, and T$_c$ is the Curie temperature. Scheme, exchange-correlation functional (XC), Hubbard parameter (U), spin-polarization (SP), magnetic ordering (Order), Metallic state (Metal) and the Code are indicated. The units of the quantities are indicated. The results calculated in the present work are denoted by *.

$^a$The datum is extracted from Ref.^67^, produced using an empirical equation proposed by Abrahams et al.

$^b$The lattice vector in [111] direction and the volume of rhombohedral unit cell, as extracted from Ref.^22^, are noteworthy for their distinct feature: the volume of the rhombohedral unit cell is one-third the size of a hexagonal structure.

$^c$The SEP predicted, in “Hypothetical verification using neural network: bandgap opening by imposing distortion”, by the neural network at zero biaxial strain based on the data extracted from Ref.^27^, see Fig. 8c.

$^d$Our previous work with SOC.

$^e$SOC is neglected.

$$^f$$Ref.^67^.

$$^g$$Ref.^90^.

$$^h$$Ref.^91^

Here, we demonstrate that the spontaneous polarizations, as predicted by the mBp or mWf methods for the FE-LM LiOsO$_3$, can be effectively fitted to the empirical equation proposed by Abrahams et al.^67^. The spontaneous polarization, $\Delta \text {P}$, and the phase transition temperature, T$_c$, play vital roles in ferroelectrics^102^. These two fundamental properties, i.e., $\Delta \text {P}$ and T$_c$, can be influenced by the atomic displacement $(\Delta z)$ of the homopolar atom, which is the most crucial quantity in “Displacive” ferroelectrics^102^. In compounds having similar symmetries, the homopolar atoms usually behave in a similar manner during ferroelectric phase transitions. Consequently, the variations in T$_c$ can be empirically estimated based on $\Delta P$^67,102,103^.

For instance, Abrahams et al.^67^ proposed the following empirical relationship between $\Delta z$ (in Å) and T$_c$ (in K):

9 $$\begin{aligned} \text {T}_c=(2.00\pm 0.09) \times 10^{4} (\Delta z)^2, \end{aligned}$$

and, additionally, they proposed the empirical relationship between $\Delta \text {P}$ (in $\upmu \text {C}/\text {cm}^2$) and $\Delta z$ (in Å):

10 $$\begin{aligned} \Delta \text {P}=(258\pm 9) \Delta z. \end{aligned}$$

By eliminating $\Delta z$ between Eqs. (9) and (10), the following empirical quadratic relationship between $\Delta \text {P}$ (in $\upmu \text {C}/\text {cm}^2$) and T$_c$ (in K) can be derived:

11 $$\begin{aligned} \text {T}_c \approx (0.300 \pm 0.020)(\Delta \text {P})^2. \end{aligned}$$

This relationship aligns with Ref.^67^ (see Sect. 7.1 of SMs for details of the derivation).

Furthermore, from experimental data, Abrahams and coworkers^67^ directly extracted the following empirical relation between $\Delta \text {P}$ (in $\upmu \text {C}/\text {cm}^2$) and T$_c$ (in K):

12 $$\begin{aligned} \text {T}_c=(0.303\pm 0.018) (\Delta \text {P})^2. \end{aligned}$$

This is consistent with Eq. (11), which was indirectly derived from Eqs. (9) and  (10).

Here, we use empirical quadratic Eq. (12), derived directly from experimental data, and calculate the spontaneous polarization for the compound under investigation. To this end, we substitute the experimentally measured T$_c=140$K for LiOsO$_3$^15^ into Eq. (12). In this way, we empirically obtain $\Delta \text {P}=(21.50 \pm 0.64)\,\upmu \text {C}/\text {cm}^2$ for the FE-LM LiOsO$_3$ (see Sect. 7.2 of SMs for details of the derivation). Our empirical result agrees with the theoretical predictions made using the mBp and mWf methods of polarization (see Table 3). This empirical evidence further strengthens the conclusion drawn at the end of “Numerical verification: consistency of mBp and mWf”. The consistency achieved based on quadratic Eq. (12), together with the agreement between results predicted by the mWf and mBp methods of polarization, helps to affirm the accuracy of our theoretical predictions. Not only does experimental data support Eq. (12) and, as a result, validate our numerical predictions, but the proportionality between $\text {T}_c$ and $(\Delta \text {P})^2$ also has strong theoretical backing, as discussed below.

### Phenomenological verification: Landau–Ginzburg theory

Landau and Ginzburg developed a phenomenological theory for the second-order phase transition in ferroelectric materials by considering spontaneous polarization as an order parameter and expressing free energy with respect to this order parameter, $\Delta \text {P}$. The free energy density $\mathscr {F}_{\Delta \text {P}}$ within this framework can be represented in the absence of external electric field and stress as follows^104,105^:

13 $$\begin{aligned} \begin{aligned} \mathscr {F}_{\Delta \text {P}}=\frac{1}{2}\alpha _0(\text {T}-\text {T}_c)(\Delta \text {P})^2+\frac{1}{4}\beta (\Delta \text {P})^4, \end{aligned} \end{aligned}$$

where the power series is truncated at the fourth order, $\alpha _0$ is a constant that depends on the materials, $\beta$ is the Landau coefficient, and the critical phase transition temperature $\text {T}_c$ is the Curie-Weiss temperature $\text {T}_C$. For the second-order phase transition, where no latent heat is present, we have viz.$\text {T}_c=\text {T}_C$. By minimizing the free energy density as follows:

14 $$\begin{aligned} \begin{aligned} \frac{\partial \mathscr {F}_{\Delta \text {P}}}{\partial ({\Delta \text {P}})} =\alpha _0(\text {T}-\text {T}_c)(\Delta \text {P})+\beta (\Delta \text {P})^3=0, \end{aligned} \end{aligned}$$

we can deduce either $\Delta \text {P}=0$, representing the equilibrium polarization of the paraelectric phase, or ta nontrivial (nonzero) equilibrium polarization of the ferroelectric phase:

15 $$\begin{aligned} \begin{aligned} \Delta \text {P}=\sqrt{\frac{\alpha _0(\text {T}_c-\text {T})}{\beta }},~~~\text {for} ~~~ \text {T}<\text {T}_c. \end{aligned} \end{aligned}$$

Thus, the Landau-Ginzburg theory of second-order phase transition (paraelectric$\leftrightarrow$ferroelectric) implies, according to Eq. (15), that:

16 $$\begin{aligned}&\text {T}_c-\text {T}=\frac{\beta }{\alpha _0}(\Delta \text {P})^2, \nonumber \\&\text {T}_c=\text {T}+\frac{\beta }{\alpha _0}(\Delta \text {P})^2, \nonumber \\&\text {T}_c \varpropto (\Delta \text {P})^2. \end{aligned}$$

We notice that the theoretical quadratic Eq. (16), which is derived from the Landau-Ginzburg theory of second order phase transition, is consistent with the empirical quadratic Eq. (12). As shown in “Empirical verification: quadratic order”, our results are also consistent with the empirical Eq. (12), derived from experimental data. Consequently, our results are theoretically supported by Eq. (16) as well.

### Empirical verification: biquadratic order

In “Phenomenological verification: Landau–Ginzburg theory”, we truncated the power series of the free energy density $\mathscr {F}_{\Delta \text {P}}$ (as expressed by Eq. (13)) at the fourth order. This resulted in a quadratic order polynomial for the transition temperature in terms of spontaneous polarization (see Eq. (16)). In “Empirical verification: quadratic order”, we used Eq. (12), which also represents a quadratic equation. However, to incorporate a broader range of interactions, including anharmonic vibrations, it would be necessary to involve higher orders of spontaneous polarization. This approach can provide a more precise tool for verifying the reliability of our SEP calculations for FE-LM LiOsO$_3$. With this in mind, let us go beyond the quadratic order. In principle, the power series of the free energy density could be truncated at the sixth order. This would, in principle, lead to a quartic (biquadratic) relation between spontaneous polarization and the transition temperature. Following this approach, we derive empirical biquadratic polynomials using available experimental $\Delta \text {P}$ and T$_c$ data for a wide range of ferroelectric compounds.

In a $\text {T}_c-(\Delta \text {P})^2$ Cartesian coordinate system, as shown in Fig. 5a, we have positioned thirteen points $(\text {T}_c, (\Delta \text {P})^2)$. Twelve of these points are based on experimental data^68,85,87,89,92–101^ for various FE perovskite semiconductors and one of them, whose experimental T$_c$ is taken from Ref.^15^, is our numerical data calculated by the mBp method for the FE metal under question. In this figure, we did not include the numerical point calculated by the mWf method because it closely aligns with that calculated by the mBp method for the FE LiOsO$_3$ metal. We only fitted a quartic-order polynomial to ten of the thirteen experimental points. These 10 experimental points are indicated by hollow-square-symbols, see Fig. 5a. Some of the experimental data^85,89,97^ have been recently measured. Fig. 5a includes two different experimental data points for LiTaO$_3$. We used one of these data points, recently measured by Zhang et al.^89^, for a better fit, as it brings the coefficient of determination (R-squared) closer to unity, see R$^2=0.97$ in Fig. 5a. Similarly, for NKbO$_3$ compound, among two different sets of experimental data included in Fig. 5a, we used only one of them, reported by Günter^96^, for a more efficient fit that brings R$^2$ closer to unity. We have excluded our numerical point, indicated by solid-square-symbol, in the fitting procedure to avoid affecting the resultant empirical quartic relation emerged from the experimental data. By this way, we have obtained the following biquadratic empirical relation:

17 $$\begin{aligned} \text {T}_c=(0.52\pm 0.06) (\Delta \text {P})^2+(-4.60\pm 1.59) \times 10^{-5}(\Delta \text {P})^4, \end{aligned}$$

where a linear behavior of T$_c$ versus $(\Delta \text {P})^2$, in agreement with Eq. (12) reported by Abrahams et al.^67^, can be observed for smaller $\Delta \text {P}$ for which the effects of the quartic term due to the factor of $10^{-5}$ can be less than the quadratic term, see Fig. 5a. However, a deviation from the linear behavior of T$_c$ with respect to $(\Delta \text {P})^2$ can be observed for larger $\Delta \text {P}$, where the quartic term can compete against the quadratic term despite the $10^{-5}$ factor. Even though the curve shown in Fig. 5a is fitted to the experimental data, and the point $(\text {T}_c, (\Delta \text {P})^2)$ corresponding to LiOsO$_3$ is absent, the fitted curve closely aligns with our numerical results calculated for LiOsO$_3$. This validates our spontaneous polarization calculated for the FE-LM LiOsO$_3$.

Additionally, we have limited the number of compounds utilized in the fitting procedure to the experimental data in Fig. 5a to just LiTaO$_3$ and LiNbO$_3$, which belong to the same family as LiOsO$_3$ displayed in Fig. 5b. These LiXO$_3$ (X=Nb, Ta) ferroelectrics, similar to LiOsO$_3$, undergo an R3c to R$\bar{3}$c phase transition and possess symmetry comparable to FE-LM LiOsO$_3$. Within these three compounds-LiNaO$_3$, LiTaO$_3$, and LiOsO$_3$-their homopolar atoms (Ta, Nb, and Os) play key roles in determining their ferroelectric properties.

A quadratic polynomial was fitted to the experimental $(\text {T}_c, (\Delta \text {P})^2)$ points considering only LiTaO$_3$ and LiNbO$_3$, resulting in the following biquadratic polynomial for $\text {T}_c$ as a function of $\Delta \text {P}$:

18 $$\begin{aligned} \text {T}_c=(0.49\pm 0.09) (\Delta \text {P})^2+(-43.84\pm 1.90) \times 10^{-5}(\Delta \text {P})^4. \end{aligned}$$

Its coefficient of determination, R$^2=0.99$, is closer to unity than R$^2=0.97$, determined for Eq.(17), indicating a better fit to the experimental data. To further test the accuracy of our results, LiOsO$_3$ was also considered. The data for LiOsO$_3$ were not used to obtain the fitted curve in Fig. 5b. However, this curve still closely approximates the point associated with LiOsO$_3$, lending credibility to our theoretical predictions.

Lebeuglea et al. reported a large SEP for the multiferroic BiFeO$_3$ compound^85^. In Fig. 5c, we have included this multiferroic compound along with the standard ferroelectric compounds from Fig. 5a. The data from Lebeuglea et al.^85^ for BiFeO$_3$ were added to Fig. 5c, and a polynomial was fitted to the expanded dataset. This process yielded the following biquadratic polynomial with R$^2=0.98$ for $\text {T}_c$ as a function of $\Delta \text {P}$:

19 $$\begin{aligned} \text {T}_c=(0.49\pm 0.03) (\Delta \text {P})^2+(-3.78\pm 0.39) \times 10^{-5}(\Delta \text {P})^4. \end{aligned}$$

This empirical polynomial also validates our calculated polarization for the LiOsO$_3$, see how the point $(\text {T}_c, (\Delta \text {P})^2)$ of LiOsO$_3$ is close to the curve in Fig. 5c.

To emphasize, Eqs. (11) and (12) from “Empirical verification: quadratic order” are quadratic in terms of $\Delta \text {P}$, while being linear in $(\Delta \text {P})^2$. The Eqs. (17) to (19) in “Empirical verification: biquadratic order”, derived from updated experimental data, are quartic (biquadratic) in $\Delta \text {P}$ and quadratic in $(\Delta \text {P})^2$. In Fig. 5(d), for comparison, the linear empirical Eq. (12), proposed by Abrahams et al.^67^, and the quadratic empirical Eqs. (17) to (19), emerged from some newer experimental data^85,89,95–97,99,101^ added to the valuable older experimental data^68,87,92–94,98,100^ in this work, are plotted in terms of $(\Delta \text {P})^2$. In addition to these 4 fitted curves included in Fig. 5d, similar to Abrahams et al.^67^ but including altogether both older and newer experimental data^68,85,87,89,92–101^ already used in Fig. 5a, we have fitted a linear polynomial for T$_c$ in terms of $(\Delta \text {P})^2$ to the experimental data, as well. This linear fit, which is also added to Fig. 5d for comparison, reads as:

20 $$\begin{aligned} \text {T}_c=(0.35\pm 0.02) (\Delta \text {P})^2, \end{aligned}$$

which aligns with Eq. (12) introduced by Abrahams et al.^67^, as shown in Fig. 5d where both Eqs. (12) and (20) are depicted. Notably, a comparison reveals that the non-linear Eqs. (17), while still in concordance with linear Eqs. (12)and (20), display linear behaviors for $\Delta \text {P}$ values approximately less than $70.71\,\upmu \text {C}/\text {cm}^2$. Lastly, at T$_c=140$ K, the SEPs predicted by these fits range from 17 to 22 $\upmu {\text {C}}/\text {cm}^2$, which fall within the linear regime, i.e., $22\, \upmu {\text {C}}/\text {cm}^2 < 70.71\, \upmu {\text {C}}/\text {cm}^2$. These values align with our SEP results predicted by mBp and mWf methods in “[Sec Sec13]”, as indicated in Table 3.

### Hypothetical verification: bandgap opening by Hubbard model using GGA+U

**Figure 6:**
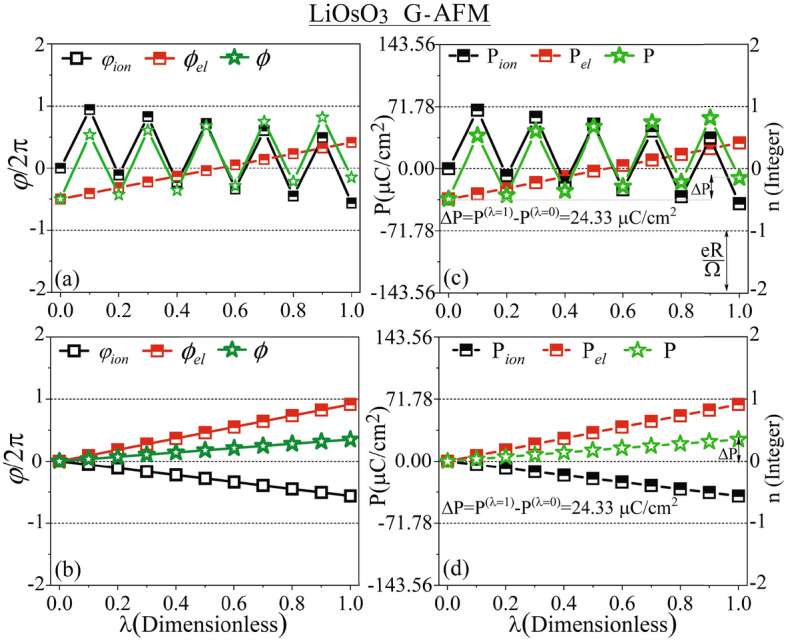
(**a**) Total, and wrapped partial Berry phases versus $\lambda$. (**b**) Unwrapped total, and partial Berry phases versus $\lambda$. All the Berry phases are divided by $2\pi$ in (**a**) and (**b**) so that the wrapping interval is converted from $[-2\pi , 2\pi ]$ to [− 1, 1] in (**a**). (**c**) Total, wrapped partial, and corresponding spontaneous polarizations versus $\lambda$. The partial polarizations are wrapped into [$-e\text {c}/{\Omega }, e\text {c}/{\Omega }$], where $e\text {c}/\Omega =71.78\,\upmu {\text {C}}/\text {cm}^2$ is the quantum of polarization. (**d**) Unwrapped total, partial, and corresponding spontaneous polarizations versus $\lambda$. The unit of polarizations is $\upmu {\text {C}}/\text {cm}^2$ in (**c**) and (**d**). Unlike Fig. 4, here, only for simplicity, the auxiliary symbols $\wedge$ and $\sim$ are not used. All the Berry phases and as a result polarizations are calculated by the standard Berry phase scheme including SP using GGA+U with $\text {U}=0.2\, \text {eV}$ for the nonmetallic state of the G-AFM LiOsO$_3$ along the distortion path as functions of structure $\lambda$ from $``\lambda =0$” to $``\lambda =1$” by step 0.1. The quantum of polarization and its number *n* are shown in (**c**) and (**d**).

**Figure 7:**
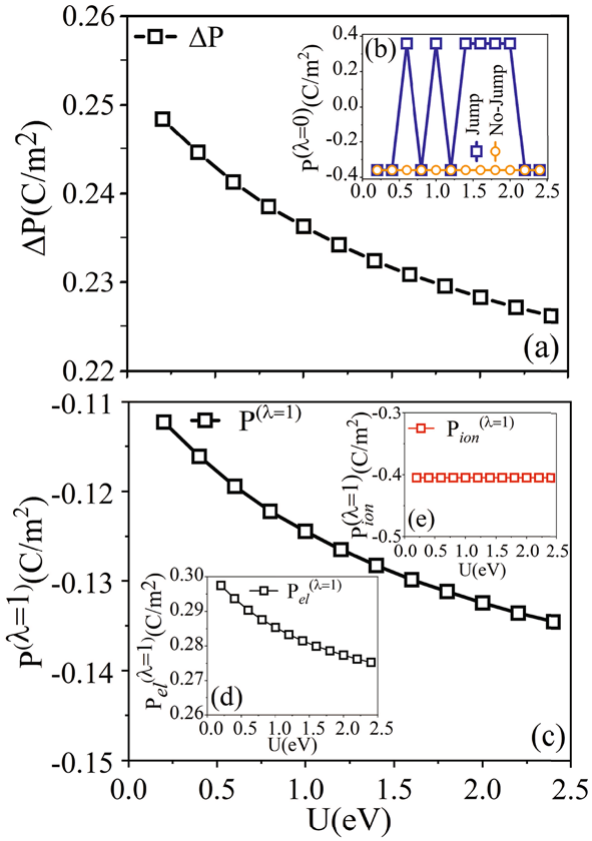
(**a**) Spontaneous polarization, $\Delta \text {P}$, (**b**) total polarization of the CS phase, $\text {P}^{(\lambda =0)}$, (**c**) total polarization of the NCS phase, $\text {P}^{(\lambda =1)}$, (**d**) electronic polarization of the NCS phase, $\text {P}^{(\lambda =1)}_\textit{el}$, and (**e**) ionic polarization of the NCS phase, $\text {P}^{(\lambda =1)}_\textit{ion}$, calculated by the standard Berry phase approach for the nonmetallic state of the G-AFM LiOsO$_3$ using GGA+U with $\text {U}\in ~[0.2, 2.4\,\text {eV}]$. The jumps in $\text {P}^{(\lambda =0)}$ occurred at $\text {U}=0.6,1.0,1.4,1.6,1.8,2.0$, as shown by empty blue square symbols in the inset (**b**), are pulled down and the blue zigzag path is made smooth as a straight orange line by subtracting one quantum of polarization, see empty orange circle symbols in the inset (**b**).

In this subsection, our objective is to calculate the SEP of LiOsO$_3$ in its hypothetical semiconductor phase. To do this, we slightly open the bandgap using PBE-GGA+U. This adjustment enables us to use the conventional Bp or Wf method without any restrictions or modifications. Our goal is to demonstrate that the SEP, as projected by the conventional Bp or Wf method for the synthetically developed G-AFM semiconductor LiOsO$_3$, is comparable to the SEPs predicted by the mBp or mWf polarization method for the naturally occurring nonmagnetic metal LiOsO$_3$. This comparison is intended to validate that our modified mBp or mWf method aligns well with the conventional Bp method, even without our modifications.

To achieve our aim, we seek to validate that the drawn conclusions are robust against changes in DFT methods and supercell configurations. Hence, we will proceed with the calculation of SEP in a manner that harmonizes more consistently with the methodologies used in this research. Thus, following our study’s approach, we will also estimate the SEP using the conventional Berry phase theory, as implemented in the WIEN2k code based on full potential. To this end, we will also utilize GGA+U to open the gap and transform the rhombohedral unit cell into a hexagonal supercell of LiOsO$_3$, as detailed in Secs. 5.2 and 5.3 of the SMs, while imposing G-type AFM ordering. In our GGA+U calculations, we will set U$_\text {eff}=0.2~\text {eV}$. This choice allows us to open the bandgaps of both polar noncentrosymmetric R3c and non-polar centrosymmetric R$\bar{3}$c structures, by minimal amounts of 0.061 eV and 0.071 eV respectively. Indeed, the aim of opening the gap was to leverage the standard Berry phase of polarization to corroborate our findings. By applying GGA+U with U$_\text {eff}=0.2~\text {eV}$, we computed the electronic, ionic, and total Berry phases for the initial, final, and the nine intermediate superstructures, as elaborated in Sect. 5.4 and illustrated in Fig. SM6 of the SMs.

As illustrated in Fig. 6a, the electronic Berry phase exhibits a linear trend, while both ionic and total Berry phases present a zigzag pattern across the array of 11 superstructures. The abrupt zigzag fluctuations in the ionic Berry phases are tempered by unwrapping sudden shifts, thus creating a smoother line, as demonstrated in Fig. 6b. We also modify the trajectory of the electronic Berry phase to ensure its origin at $``\lambda =0$” aligns with the zero-point, as shown in Fig. 6b. The total Berry phase is then obtained by combining the electronic and ionic Berry phases (Fig. 6b).

The initial or misplaced polarizations, visible in Fig. 6c, are adjusted as depicted in Fig. 6d. This specific method and its aim are extensively detailed in “[Sec Sec13]” and visually illustrated in Fig. 4, so further elaboration here is redundant. In Fig. 6, we have intentionally omitted auxiliary symbols $\wedge$ and $\sim$, as discussed in “[Sec Sec13]”.

Upon comparing Fig. 6c and d, we find that the SEP, calculated by considering only the initial $``\lambda =0$” and the final $``\lambda =1$” structures, aligns with the SEP computed across all 11 structures, evidenced by the identical value of $\Delta \text {P}=24.33\, \upmu \text {C}/\text {cm}^2$. Hence, the consideration of intermediate superstructures, or adjustment of abrupt zigzag patterns, is redundant for this compound. However, this finding should not be generalized, as the SEP may vary when intermediate structures are included, as demonstrated in Fig. 4c,d.

Interestingly, the phases (polarizations) for the G-AFM phase fall within the range $[-2\pi , 2\pi ~\text {Rad}]([-71.78, 71.78\, \upmu \text {C}/\text {cm}^2])$ (Fig. 6a,b), while the phases (polarizations) for the NM phase are confined to $[-\pi , \pi \text {Rad}]([-71.78/2, 71.78/2\, \upmu \text {C}/\text {cm}^2])$ (Fig. 4a,b). This discrepancy stems from spin-polarized calculations for the G-AFM phase (Fig. 6a,b) versus non-spin-polarized calculations for the NM phase (Fig. 4a,b). In spin-polarized calculations, the Berry phases (and corresponding polarizations) for spin-up states are summed with those for spin-down states, causing the doubling in wrapping intervals due to the additional spin degree of freedom. However, this factor does not affect the calculation of $\Delta \text {P}$, where the difference between initial and final polarizations is calculated. Our computed SEP, $\Delta \text {P}=24.33\, \upmu \text {C}/\text {cm}^2$ for the nonmetallic state, aligns with the SEP calculated for the metallic state of LiOsO$_3$, $\Delta \text {P}=22.77\,\upmu \text {C}/\text {cm}^2$, as seen in Fig. 4d and Table 3, along with Fig. 6d.

Continuing our analysis, we acknowledge the work of He et al., who calculated the SEP as $22.23\,\upmu \text {C}/\text {cm}^2$^22^. Their research relied on the standard Berry phase theory^52,55^ and employed the pseudopotential-based VASP code^46–50^. Using LSDA+U with $\text {U}_\text {eff}=2~\text {eV}$, they established a $2\times 2\times 2$ supercell derived from the rhombohedral unit cell of G-AFM LiOsO$_3$.

The computed SEP of $22.23\,\upmu \text {C}/\text {cm}^2$ for the conjectured G-AFM insulating LiOsO$_3$ aligns with our SEP for the authentic nonmagnetic metal LiOsO3, calculated as $22.27~(23.18)\,\upmu \text {C}/\text {cm}^2$ by the mBp (mWf) method of polarization we have developed (see Fig. 4d and Table 3). This compatibility validates our mBp and mWf methods and indicates that the influence of GGA+U on the class I$^*$, where the valence bands cross the Fermi level, is limited (refer to Fig. 1 and Table 1, where $\textbf{P}_{\textit{el}}^{(\text {I}^*)}$ is reported as a moderate value of $0.0746~\text {C}/\text {cm}^2$ for the polar NCS R3c structure).

Before concluding this subsection, we will examine the implications of U on polarization in greater depth. Additionally, see Sect. 5.6 of the SMs for a discussion regarding the influence of U on lattice parameters and the energy bandgap.

Our investigation reveals a marginal discrepancy between the SEP obtained using GGA+U with U$_\text {eff}=0.2~\text {eV}$ and that determined by He et al.^22^ using LDA+U with U$_\text {eff}=2.0~\text {eV}$, where $\Delta \text {P}=24.33 > 22.23\,\upmu \text {C}/\text {cm}^2$ (refer to Table 3). Interestingly, the SEP value acquired by He et al. aligns more closely with our PBE-GGA calculated outcome for the metallic state than with our GGA+U calculations involving U$_\text {eff}=0.2~\text {eV}$, such that $\Delta \text {P}= 22.77 \approx 22.23~\upmu \text {C}/\text {cm}$.

In alignment with the procedure employed by He et al., we recalculated the SEP using GGA+U with U$_\text {eff}=2.0~\text {eV}$. The resultant SEP value, $\Delta \text {P}= 22.32~\upmu \text {C}/\text {cm}$, displays superior concurrence with the findings reported by He et al., as well as with our mBp and mWf polarization-derived SEPs in the metallic state. These shared findings indicate $\Delta \text {P}= 22.32 \approx 22.77 \approx 22.23~\upmu \text {C}/\text {cm}$ (refer to Table 3 and Fig. 4d).

Nonetheless, it is essential to remember that we applied the PBE-GGA functional and the FP-APW+lo method, differing from those used in Ref.^22^. Thus, although the closer agreement when using the identical U value is not surprising, given these divergences in computational methodology, it warrants mention.

Given the above analysis, the effects of U parameter on the SEP are significant, albeit not necessarily large. Therefore, we have systematically studied these effects, as promised earlier. We have calculated $\Delta \text {P}$ by GGA+U for a range of U from 0.2 to 2.4 eV in steps of 0.2 eV, as shown in Fig. 7a. Our findings reveal a decrease in $\Delta \text {P}$ with increasing U.

This reduction is attributable to the electronic part of the polarization of the polar NCS R3c phase, which corresponds to the final structure $``\lambda =1$”. For clarification, let’s examine the changes in the electronic and ionic parts of the Berry phases in both the initial $``\lambda =0$” and final $``\lambda =1$” structures.

Our results computed by GGA+U with U$_\text {eff}=0.2$ reveal that $\text {P}_{\textit{ion}}^{(\lambda =0)}=0$ and $\text {P}_{\textit{el}}^{(\lambda =0)}= -e\text {c}/{2\Omega }$. Additional results, not presented here, show that $\text {P}_{\textit{ion}}^{(\lambda =0)}$ and $\text {P}_{\textit{el}}^{(\lambda =0)}$ remain constant over the examined range of U, yielding $\text {P}_{\textit{ion}}^{(\lambda =0)}=0$ and $\text {P}_{\textit{el}}^{(\lambda =0)}=-e\text {c}/{2\Omega }$ for $\text {U}\in [0.2, 2.4~\text {eV}]$. Thus, the total polarization for $``\lambda =0$”, $\text {P}^{(\lambda =0)}=0-e\text {c}/{2\Omega }=-e\text {c}/{2\Omega }$, remains unaltered with variation in U over the examined range, as depicted in Fig. 7b.

The total polarizations for the initial structure $``\lambda =0$”, represented by blue empty square symbols in Fig. 7b, show fluctuations between $-e\text {c}/{2\Omega }$ and $+e\text {c}/{2\Omega }$. By subtracting a quantum of polarization, $+e\text {c}/{\Omega }$, from $\text {P}^{(\lambda =0)}=e\text {c}/{2\Omega }$ at selected $\text {U}$ values, we smooth the path of polarization.

Therefore, $\text {P}^{(\lambda =0)}=-e\text {c}/{2\Omega }$ remains constant for $\text {U}\in [0.2, 2.4~\text {eV}]$, as indicated by orange empty circle symbols in Fig. 7b. This indicates that $\text {P}^{(\lambda =0)}$ remains constant at half of the quantum of polarization and is not affected by variations in U for $\text {U}\in [0.2, 2.4\text {eV}]$. Hence, the variation of $\Delta \text {P}$ cannot be attributed to the non-polar CS R$\bar{3}$c structure and originates solely from the polar NCS R3c structure.

Our GGA+U results indicate that $\text {P}_{\textit{ion}}^{(\lambda =1)}$ remains constant at $-0.40482~\upmu \text {C}/\text {cm}^2$ for $\text {U}\in [0.2, 2.4~\text {eV}]$, while $\text {P}_{\textit{el}}^{(\lambda =1)}$ decreases as U increases, as depicted in Fig. 7d. Consequently, the variation of $\Delta \text {P}$ with U is solely attributable to the variation of $\text {P}_{\textit{el}}^{(\lambda =1)}$ with U, as shown by the comparison of Fig. 7a with Fig. 7c.

The band structures calculated by PBE-GGA for the NM phase and by GGA+U with $\text {U}=0.2~\text {eV}$ and $\text {U}=2~\text {eV}$ for the G-AFM phase, as presented in Fig. SM1 of the SMs, reveal 18 bands within the energy range of [−- 2, 2 eV]. Our calculations show minimal changes to the valence bands with increasing U in GGA+U, while the conduction bands move away from the Fermi level. Our findings suggest that the variation in SEP is primarily due to changes in the electronic part of the NCS phase.

In conclusion, our study articulates the validity of SEPs as projected by the mBp and mWf methodologies for the NM metallic phase of LiOsO$_3$. We have demonstrated that these SEPs can be accurately approximated and corroborated using the generalized gradient approximation plus Hubbard U (GGA+U) for the contrived G-AFM nonmetallic phase of LiOsO$_3$. Furthermore, it has been discerned that the influence of diminutive U parameters on the SEP is relatively marginal, permitting us to open the bandgap of the G-AFM phase through GGA+U and estimate the magnitude of SEP for the NM metallic LiOsO$_3$. Therefore, our endeavors have successfully met the stated objectives.

### Hypothetical verification using neural network: bandgap opening by imposing distortion

**Figure 8:**
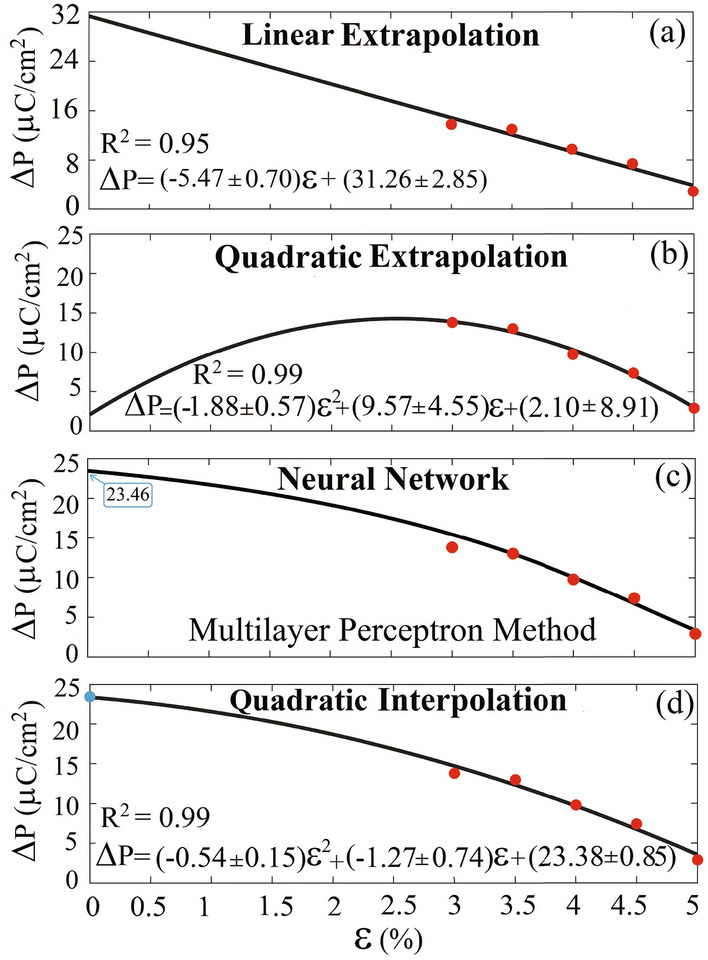
SEP, $\Delta \text {P}$, as a function of tensile biaxial strain, $\upvarepsilon$, imposed on LiOsO$_3$. The data, indicated by red points from $+3\%$ to $+5\%$ by step $+0.5\%$, are extracted from Fig. 3b of Ref.^27^. Using these extracted data only, at $\upvarepsilon =0\%$ the SEP is predicted to be (**a**) $\Delta \text {P}=31.26 \pm 2.85\,\upmu \text {C}/\text {cm}^2$ by the linear extrapolation, (**b**) $\Delta \text {P}=2.10 \pm 8.91\, \upmu \text {C}/\text {cm}^2$ by the quadratic extrapolation, and (**c**) $\Delta \text {P}=23.46 \,\upmu \text {C}/\text {cm}^2$ by the neural network. (**d**) After including the point $(\upvarepsilon =0\%, \Delta \text {P}=23.46 \,\upmu \text {C}/\text {cm}^2)$, predicted by the neural network in (**c**), as indicated by a blue point here in (**d**), to the set of red points, extracted from Fig. 3b of Ref.^27^, at $\upvarepsilon =0\%$ the SEP is predicted to be $\Delta \text {P}=23.38 \pm 0.85\,\upmu \text {C}/\text {cm}^2$ by the quadratic interpolation.

Here, we present an alternative strategy for bandgap modulation, complementing the GGA+U methodology described in “Hypothetical verification: bandgap opening by Hubbard model using GGA+U”. It involves the application of appropriate strain to the system. Zhang et al.^27^ examined the correlation between the bandgap and biaxial strain in LiOsO$_3$, maintaining consistent crystal symmetry. Their results demonstrated a direct relationship between the increase in the percentage of tensile biaxial strain, represented as $\upvarepsilon ~(\%)$, and the augmentation of the bandgap.

On expanding the bandgap, Zhang et al.^27^ proceeded to calculate the SEP of the insulated LiOsO$_3$, using the Berry phase theory^52,55^ as implemented in the pseudopotential-based VASP code^46–50^. The SEP was measured as a function of $\upvarepsilon ~(\%)$ ranging from $+3$ to $+5\%$ in steps of $+0.5\%$.

The data obtained from Zhang et al.’s work^27^ were used to predict the SEP value at $\upvarepsilon =0$ using linear extrapolation, quadratic extrapolation, and neural network machine learning techniques. The data points were represented as five red markers spaced at increments of $+0.5\%$ from $+3$ to $+5\%$ in Fig. 8. Linear regression yielded the equation $\Delta \text {P}=[(-5.47 \pm 0.70) \upvarepsilon +(31.26 \pm 2.85)]\upmu \text {C}/\text {cm}^2$, as depicted in Fig. 8a.

However, the limitations of linear extrapolation became apparent when comparing the rate of change of $\Delta \text {P}$ at different $\upvarepsilon$ values. A quadratic fit was applied, resulting in the equation $\Delta \text {P}=[(-1.88 \pm 0.57) \upvarepsilon ^2 +(9.57 \pm 4.55)\upvarepsilon +(2.10 \pm 8.91)]\upmu \text {C}/\text {cm}^2$, as seen in Fig. 8b. Despite an improved $R^2$ value for the quadratic function, the relative error remained significantly high.

Given these limitations, the study turned to machine learning, employing the Bayesian regularization-trained multilayer perceptron (MLP) methodology^106–111^. The MLP procedure applied Bayesian activation function to optimize the weights and reduce the error function $E(O)=T-f(IW_{io})$. The network output vector *O* is computed as $O=f(IW_{io})$. After the training, the predicted SEP at $\upvarepsilon =0$ was $23.46~\upmu \text {C}/\text {cm}^2$, as shown in Fig. 8c, consistent with the predictions made by the mBp and mWf methods.

Lastly, a refined empirical quadratic equation was derived for $\Delta \text {P}$ as a function of strain $\upvarepsilon$ by including the prediction from the neural network, yielding:

21 $$\begin{aligned} \Delta \text {P}=[(-0.54\pm 0.15)\upvarepsilon ^2 + (-1.27 \pm 0.74)\upvarepsilon + (23.38 \pm 0.85)], \end{aligned}$$

which was graphed in Fig. 8d. This model’s prediction at $\upvarepsilon =0$ aligns closely with the neural network prediction, as well as the predictions made by the mBp and mWf methods, providing validation for these methodologies.

### Comparative verification: BiFeO$_{{\textbf {3}}}$, LiNbO$_{{\textbf {3}}}$, and LiTaO$_{{\textbf {3}}}$

**Figure 9:**
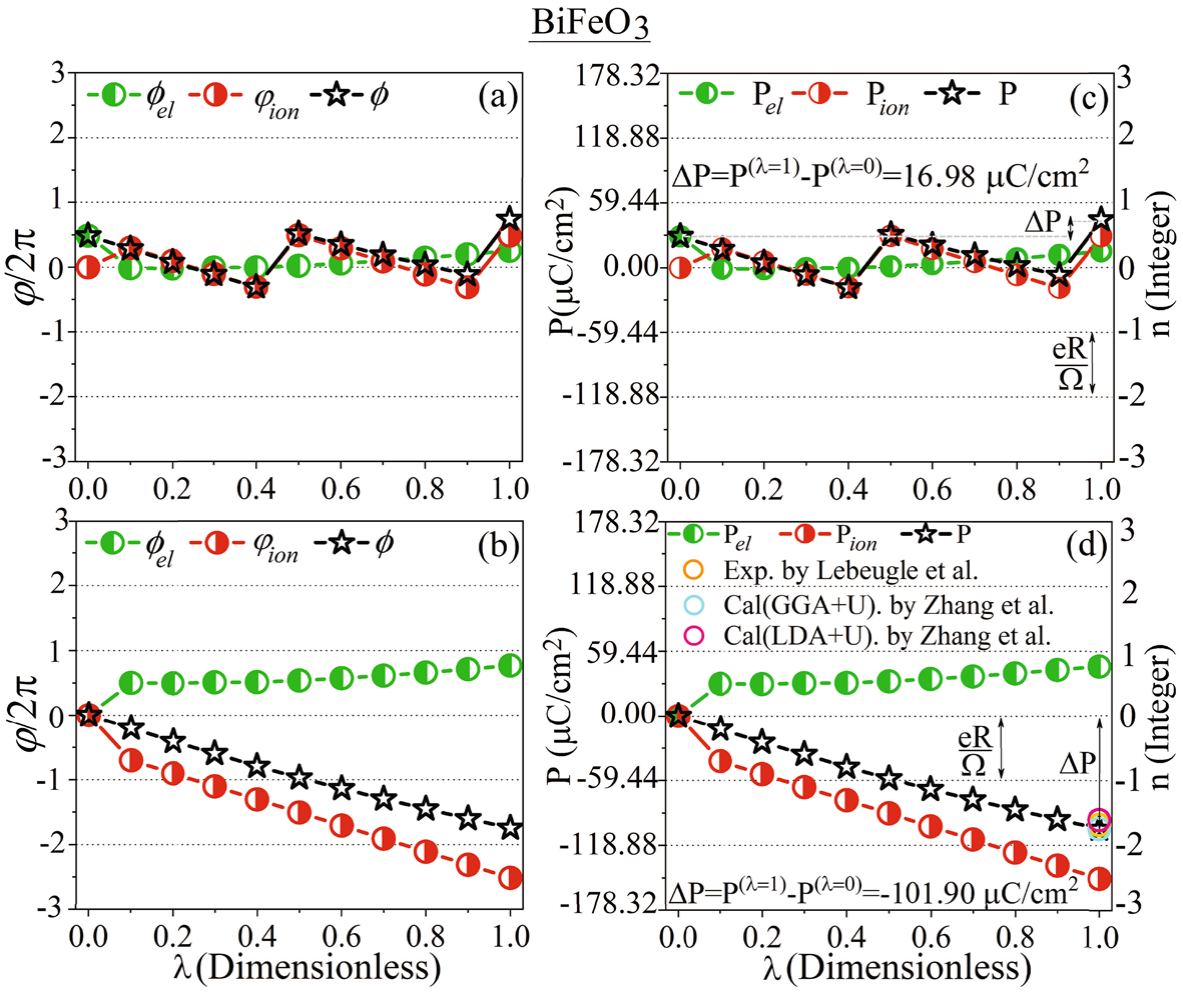
(**a**) Total, and wrapped partial Berry phases versus $\lambda$. (**b**) Unwrapped total, and partial Berry phases versus $\lambda$. All the Berry phases are divided by $2\pi$ in (**a**) and (**b**) so that the wrapping interval is converted from $[-\pi , \pi ]$ to [− 0.5, 0.5] in (**a**). (**c**) Total, wrapped partial, and corresponding spontaneous polarizations versus $\lambda$. The partial polarizations are wrapped into [$-e\text {c}/2{\Omega }, e\text {c}/2{\Omega }$], where $e\text {c}/\Omega =59.44\,\upmu {\text {C}}/\text {cm}^2$ is the quantum of polarization. (**d**) Unwrapped total, partial, and corresponding spontaneous polarizations versus $\lambda$. The unit of polarizations is $\upmu {\text {C}}/\text {cm}^2$ in (**c**) and (**d**). Like Fig. 6 but unlike Fig. 4, here, only for simplicity, the auxiliary symbols $\wedge$ and $\sim$ are not used. All the Berry phases and as a result polarizations are calculated by the standard Berry phase scheme including SP and SOC by GGA+U with $\text {U}_\text {eff}=4~\text {eV}$ for the Multiferroic G-AFM BiFeO$_3$ along the distortion path as functions of structure $\lambda$ from $``\lambda =0$” to $``\lambda =1$” by step 0.1. The quantum of polarization and its number *n* are shown in (**c**) and (**d**). Due to the SOC, the scaled wrapping interval, $[-0.5, 0.5]$, is obtained to be half of the interval $[-1, 1]$. Experimental^85^ and theoretical^84^ SEPs are included for comparison.

**Figure 10:**
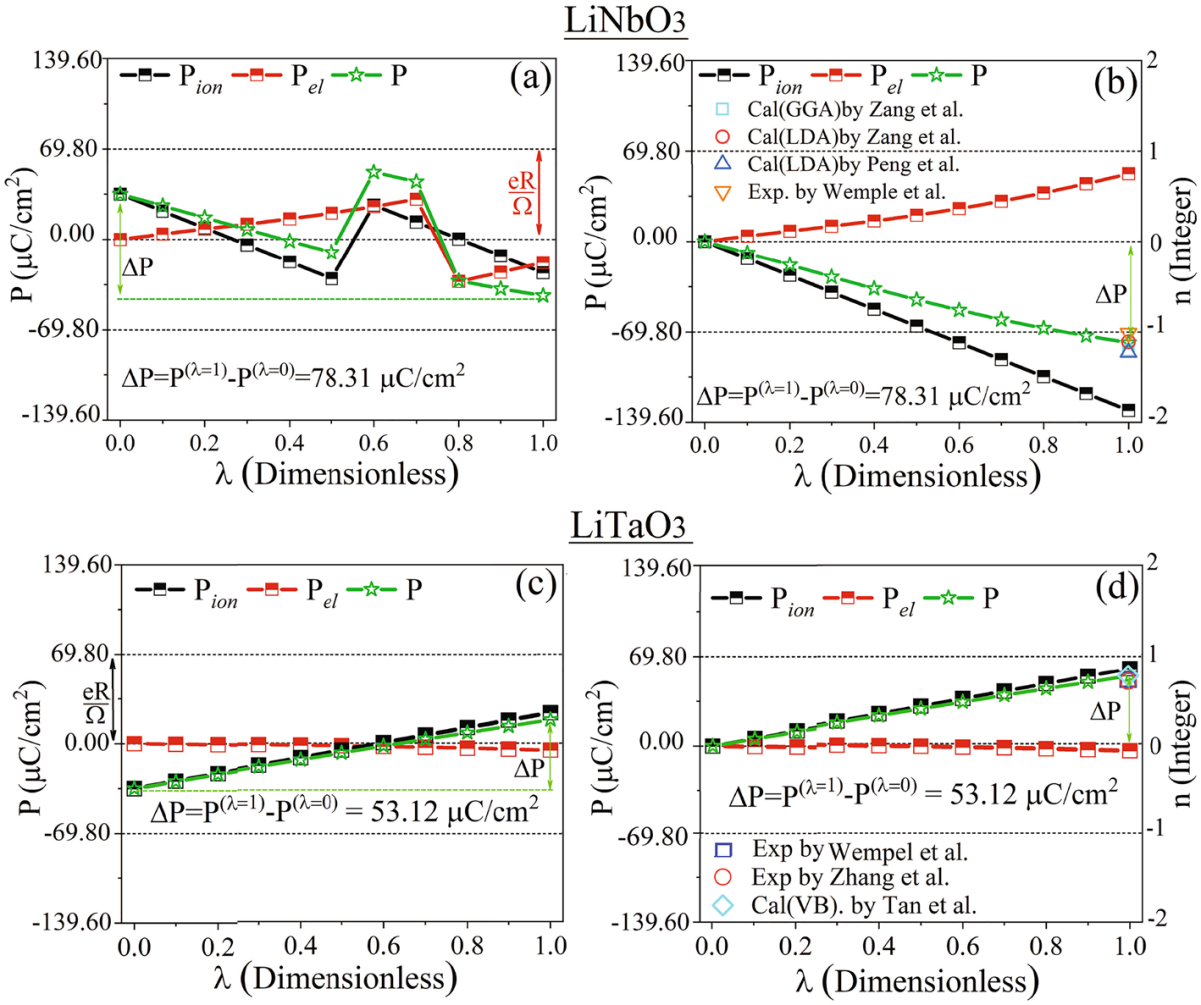
Total, wrapped partial, and corresponding spontaneous polarizations versus $\lambda$ for (**a**) LiNbO$_3$ and (**c**) LiTaO$_3$. The partial polarizations are wrapped into [$-e\text {c}/{\Omega }, e\text {c}/{\Omega }$], where $e\text {c}/\Omega =69.80\,\upmu {\text {C}}/\text {cm}^2$ is the quantum of polarization. Unwrapped total, partial, and corresponding spontaneous polarizations versus $\lambda$ for (**b**) LiNbO$_3$ and (**d**) LiTaO$_3$. The unit of polarizations is $\upmu {\text {C}}/\text {cm}^2$. Like Figs. 6 and 9 but unlike Fig. 4, here, only for simplicity, the auxiliary symbols $\wedge$ and $\sim$ are not used. All the Berry phases and as a result polarizations are calculated by the standard Berry phase scheme including SP by PBE-GGA for the normal FE LiNbO$_3$ and by LDA for the normal FE LiTaO$_3$ along the distortion path as functions of structure $\lambda$ from $``\lambda =0$” to $``\lambda =1$” by step 0.1. For comparison, experimental^87,87,89^ and theoretical^84,86,88^ SEPs are included in (**b**) for LiNbO$_3$ (in (**d**) for LiTaO$_3$).

In this section, we aim to validate the efficacy of our unwrapping procedure in determining the most suitable branch for the unique prediction of the resultant SEP, as elucidated and applied to LiOsO$_3$ in *[Sec Sec13]” for its actual metallic state, and in “Hypothetical verification: bandgap opening by Hubbard model using GGA+U” and “Hypothetical verification using neural network: bandgap opening by imposing distortion” for its conjectural nonmetallic state.

To facilitate this, we have selected the normal ferroelectrics BiFeO$_3$, LiNbO$_3$, and LiTaO$_3$ for comparison. These compounds, similar to LiOsO$_3$, undergo the R$\bar{3}$c-R3c ferroelectric transition. This transition, as detailed in Sect. 5.4 of the SMs, facilitates the creation of their intermediate structures in a manner analogous to LiOsO$_3$. We simulate this transition from $\lambda =0$ to $\lambda =1$, passing through the intermediate structures $\lambda =0.1, 0.2,...,0.9$ as illustrated in Fig. SM6 of the SMs.

Contrary to LiOsO$_3$, the SEPs of these selected compounds have been both experimentally measured^85,87,89^ and theoretically computed^84,88^. The availability of experimental data provides us the opportunity to evaluate the accuracy of our unwrapping and uniquifying process in the context of real-world results.

We begin our analysis with the multiferroic BiFeO$_3$, drawing extensively from our previous work^78^. For its G-AFM phase, we calculate the Berry phases and polarizations across all 11 superstructures depicted in Fig. SM6 of the SMs. These calculations, conducted via GGA+U methodology with an optimized $\text {U}_\text {eff}=4~\text {eV}$, are presented in Fig. 9. Figure 9a presents the total and wrapped partial electronic and ionic components of the Berry phases, scaled by $2\pi$.

The scaled wrapping interval for G-AFM BiFeO$_3$, $[-0.5, 0.5]$, is precisely half of that for G-AFM LiOsO$_3$, $[-1, 1]$, as observed when comparing Fig. 9a with Fig. 6a. Spin-orbit coupling (SOC) demonstrates a greater influence on G-AFM BiFeO$_3$ than G-AFM LiOsO$_3$, thus our computations for G-AFM BiFeO$_3$ (G-AFM LiOsO$_3$) incorporate (exclude) SOC, as illustrated in Fig. 9 (Fig. 6).

Relativistic quantum mechanics elucidates that the DOSs of spins up and down either combine or separate in accordance with the presence or absence of SOC^112–114^. Consequently, Berry phases, polarizations, and other electronic properties either couple or decouple accordingly, necessitating the choice of a relativistic ($|j, m_j, l, s\rangle$) or non-relativistic basis ($|l, m_l, s, m_s\rangle$).

In this context, the wrapping interval of Fig. 6a is twice that of Fig. 9a, given the corresponding quanta of polarizations. Fig. 9c illustrates the changes in the wrapped polarizations across the 11 superstructures.

The uncertainty problem arising from the calculated wrapped SEP of $16.98~\upmu \text {C}/\text {cm}^2$, which differs significantly from the experimental value of $100.00~\upmu \text {C}/\text {cm}^2$^84^, requires resolution via identification of the optimal branch, or integer quantum number *n*.

We apply an unwrapping procedure (analogous to that discussed in “[Sec Sec13]”) to the Berry phases and polarizations, as depicted in Fig. 9b,d, respectively. By comparing total polarizations at $``\lambda =1$” before and after wrapping, or equivalently total Berry phases, we discern the integer quantum number *n* to be $-2$.

Therefore, taking into account $n=-2$ and the quantum of polarization $59.44~\upmu \text {C}/\text {cm}^2$, the SEP derived from the unwrapped total polarization $16.98~\upmu \text {C}/\text {cm}^2$ is calculated to be $-101.90~\upmu \text {C}/\text {cm}^2$.

Our SEP calculation using the density functional and standard Berry phase theories with GGA+U and $\text {U}_\text {eff}=4~\text {eV}$ is consistent with both experimental data^85^ and theoretical results^84^. This congruence affirms the validity of our unwrapping and uniquifying procedure. We also replicated standard Berry phase calculations using GGA+U with $\text {U}_\text {eff}=2~\text {eV}$, and found an increase in $|\Delta \text {P}|$ as U decreases from 4 to 2 eV, affirming the $\Delta \text {P}$-U relationship depicted in Fig. 7a.

In this investigation, we consider the typical ferroelectrics (FEs) LiNbO$_3$ and LiTaO$_3$. These materials exhibit equivalent electronic configurations, similar chemical compositions, and undergo identical R$\bar{3}$c-R3c ferroelectric transitions as LiOsO$_3$^84,86^. Their spontaneous electrical polarizations (SEPs) have been documented experimentally. In Fig. 10a–d, both the wrapped and unwrapped or shifted total and partial polarizations as a function of superstructures $\lambda = 0, 1, 2, \ldots , 1$ are represented for LiNbO$_3$ and LiTaO$_3$, respectively.

These manipulations do not modify the SEPs for LiNbO$_3$ and LiTaO$_3$; for both, the shift *n* is null, demonstrated by an equivalent $78.31~\upmu \text {C}/\text {cm}^2$ value for LiNbO$_3$ and $53.12~\upmu \text {C}/\text {cm}^2$ for LiTaO$_3$, observed in Fig. 10a–d for $\Delta \text {P}$.

The G-AFM FE LiOsO$_3$, with a bandgap, also exhibits $n=0$, illustrated by a constant $24.33~\upmu \text {C}/\text {cm}^2$ value for $\Delta \text {P}$ in Fig. 6c,d^84,86^. Conversely, the NM FE-LM LiOsO$_3$ and the GAM normal multiferroic BiFeO$_3$ display $n=1\ne 0$ and $n=-2\ne 0$, respectively. For these cases, the SEPs differ as shown by the distinct values extracted from the respective figures for $\Delta \text {P}$. This indicates that the unwrapping or shifting procedure modifies “$\text {P}^{(\lambda =1)}-\text {P}^{(\lambda =0)}$” unless the optimal branch is chosen, in which case it remains consistent.

For LiNbO$_3$ (Fig. 10a), some discontinuities are noted, while for LiTaO$_3$ (Fig. 10c), these are absent. This suggests a $n=0$ for LiNbO$_3$, as the difference “$\text {P}^{(\lambda =1)}-\text {P}^{(\lambda =0)}$” is impervious to a simple shift. However, this is not solely dependent on the initial and final structures $``\lambda =0$” and $``\lambda =1$”, and necessitates examination of intermediate structures to guarantee the selection of the optimal branch.

Theoretical data and experimental values at room temperature for LiNbO$_3$ are presented in Fig. 10b and Table 3, offering a comparative analysis. Our results align with existing theoretical research and experimental data^84,86,87^. Likewise, for LiTaO$_3$, our findings accord with the experimental SEPs measured by Wemple et al.^87^, and Zhang et al.^89^, and the theoretical datum calculated by Tan et al.^88^. Notably, these consistencies between theoretical and experimental results extend further when considering that the theoretical DFT results were computed at zero temperature while experimental data were obtained at room temperature. Reduction in temperature can decrease entropy and enhance the electrical order, thus potentially increasing the experimentally measured polarization. This, in turn, can improve the consistency between theoretical results (calculated at zero temperature) and experimental data (recorded at lower temperatures).

The ferroelectric structural distortion in LiXO$_3$ perovskites ($\text {X}=\text {Nb},\text {Ta}, \text {Os}$) has been linked to the hybridization of O:p and X:d orbitals^22,84,115^. The degree of this distortion is influenced by the eccentricity of the X atom, as measured by the *c*/*a* ratio, a recognized indicator of structural distortion strength^84^, and outlined in “[Sec Sec10]”.

In the LiXO$_3$ family, the computed *c*/*a* ratios are 2.69, 2.67, and 2.64 for LiNbO$_3$, LiTaO$_3$, and LiOsO$_3$ respectively, illustrating a decrease in structural distortion with decreasing *c*/*a* ratio. This trend also corresponds to a decrease in the spontaneous electric polarization (SEP). Hence, the smaller SEP of LiOsO$_3$ can be substantiated when compared to the larger SEPs of LiNbO$_3$ and LiTaO$_3$, i.e. $(c/a){\text {LiNbO}3}=2.64>(c/a){\text {LiTaO}3}=2.67>(c/a){\text {LiOsO}3}=2.64 \Rightarrow \Delta ({\text {P}}){\text {LiNbO}3}=78.31~\upmu \text {C}/\text {cm}^2>(\Delta {\text {P}}){\text {LiTaO}3}=53.12~\upmu \text {C}/\text {cm}^2>(\Delta {\text {P}}){\text {LiOsO}_3}=22.77~\upmu \text {C}/\text {cm}^2$, as shown in Table 3.

However, this comparison should not be generalized for compounds with different correlations. While the SEP decreases with an increase in U for LiOsO$_3$ (with a constant *c*/*a* ratio, as depicted in Fig. 7), it may increase with U if the *c*/*a* ratio is not fixed due to its own increase with U (as shown in Table (SM2) of the SMs). Hence, the SEP is influenced by both the *c*/*a* ratio and U, and its behavior relative to U is contingent upon whether the *c*/*a* ratio is held constant. This necessitates caution when extrapolating these results to other cases.

## Conclusion

In this work, we have explored a groundbreaking possibility: the existence of nonzero spontaneous electric polarization (SEP) in metals. We have challenged the widely accepted belief that itinerant electrons invariably annihilate ferroelectricity in metals. Our work builds on the theoretical conjecture of Anderson and Blount, and experimental findings of Y. Shi and team, offering a quantitative validation.

Our research’s pivot is the adjustment of existing methods, namely the Berry phase theory and Wannier functions theory, for calculating electric polarization in systems with nonzero bandgaps. By addressing their limitations and modifying these methods, we have developed the modified Berry phase (mBp) theory of polarization and modified Wannier functions (mWf) theory of polarization. These adapted methods are poised to work effectively in predicting the SEP of metals.

In the case study of the ferroelectric-like metal (FE-LM), lithium osmate (LiOsO$_3$), our calculated SEP demonstrates an alignment with the SEP in Barium Titanate (BaTiO$_3$), a regular ferroelectric compound. The consistency with both empirical and theoretical data underscores the validity of the mBp and mWf methods. We further validated our findings via multiple approaches: numerical verification, empirical testing, comparison with the Landau-Ginzburg theory, hypothetical adjustments to the bandgap, and comparative analysis with normal ferroelectric materials.

Notably, due to the absence of SEP at zero biaxial strain, we employed the multilayer perceptron method - a subset of the feedforward artificial neural network class in machine learning - to project the SEP at this state. This prediction was based on available data for nonzero strains.

Overall, the consistency across all validation methods implies a high likelihood of our predictions being accurate. As such, our proposed mBp and mWf methods can be reliably applied to predict SEP in metals. This opens up avenues for the theoretical identification and practical synthesis of new ferroelectric-like metals, enhancing the broader understanding of ferroelectricity, as aptly expressed by Evgeny Y. Tsymbal. Our research marks a significant contribution to the continuous enrichment of knowledge in physics and material science, 100 years after the original discovery of ferroelectricity.

### Supplementary Information

Supplementary Information.

## Data Availability

All data analyzed during this study are included in this published article and its supplementary material. In addition, to enhance the reproducibility and transparency of the results, the raw data sets utilized in the present study are also available from the corresponding author upon reasonable request.
