# Supplementary material for: Nonzero spontaneous electric polarization in metals: novel predictive methods and applications
Source: Sci Rep. 2024 Jan 5;14:672. doi: 10.1038/s41598-023-49463-w (PMC10770415; doi:10.1038/s41598-023-49463-w)
Supplement: Supplementary file 1 — Supplementary Information. [file 41598_2023_49463_MOESM1_ESM.pdf]

Supplementary Materials (SMs): Nonzero spontaneous electric polarization in metals: Novel predictive methods and applications

Shahrbano Rahimi[1], S. Jalali-Asadabadi[1,*], Peter Blaha[2], and Farhad Jalali-Asadabadi[1]

[1]Department of Physics, Faculty of Physics, University of Isfahan (UI), Hezar Jerib Avenue, Isfahan 81746-73441, Iran. Fax: +98-031-37934800; Tel: +98-031-37934776
[*]sjalali@sci.ui.ac.ir
[2]Institute of Materials Chemistry, Vienna University of Technology, Getreidemarkt 9/165-TC, A-1060 Vienna, Austria

ABSTRACT

In the ”Supplementary Materials (SMs)” document, we provide additional information to support the discussions presented in the main manuscript. The supplementary material covers various aspects, including the theoretical background, computational methodologies, crystal structures, dynamical stability analysis, and empirical remarks. Specifically, the document discusses the theoretical background, which includes the Berry phase (Bp) and Wannier functions (Wf) approaches. We also address the obstacles and demands related to the study. The main objective of this SMs document is to provide a detailed description of the extended electric polarization methodology for ferroelectric-like metals, which involves the modified Berry phase (mBp) method and modified Wannier (mWf) method of polarization. The computational details of our study are outlined, and we present the crystal structures of interest, including rhombohedral and hexagonal structures. We compare the rhombohedral and hexagonal structures and analyze nine intermediate structures. The G-type antiferromagnetic structure is also discussed, along with the structural and electronic properties of $LiOsO_3$. Furthermore, we examine the dynamical stability or instability of the polar noncentrosymmetric R3c phase and the nonpolar centrosymmetric R$\bar{3}$c phase in $LiOsO_3$. We provide empirical remarks, including the derivation of empirical Eq.(11) from Eqs.(9) and (10) of the main text and the accuracy of spontaneous electric polarization from empirical Eq. (12) of the main text. Overall, the supplementary material enhances the understanding and provides additional supporting information for the main manuscript.

1 THEORETICAL BACKGROUND

In the modern theory of polarization [1, 2, 3, 4, 5, 6, 7, 8, 9], the total microscopic polarization of structure λ is defined as follows:

$$\mathbf{P}^{(\lambda)} = \frac{e}{\Omega^{(\lambda)}} \sum_{s=1}^{\mathbb{N}} Z_s^{(\lambda)} \mathbf{r}_s^{(\lambda)} + \frac{2e}{(2\pi)^3} \sum_{n=1}^{\mathbb{M}} \int_{1\mathrm{BZ}^{(\lambda)}} d\mathbf{k} \langle u_{n,\mathbf{k}}^{(\lambda)} | -i\nabla_{\mathbf{k}} | u_{n,\mathbf{k}}^{(\lambda)} \rangle, \tag{1}$$

where λ, which can be 0 and 1, refers to the initial non-polar CS R$\bar{3}$c structure and final polar noncenrosymmetric R3c phase with lower-symmetry than the R$\bar{3}$c; *viz.* $(\lambda = 0) \equiv$ (R$\bar{3}$c) and $(\lambda = 1) \equiv$ (R3c). In this equation, e is the electron charge, $\Omega^{(\lambda)}$ is the unit cell volume of the structure λ, s is an index to numerate number of ions in the unit cell, $\mathbb{N}$ is the number of ions. In the first term of this equation, $Z_s^{(\lambda)}$ is the ionic number in the unit cell of the phase, *i.e.*, the number of valence electrons of atom s in structure λ so that $eZ_s^{(\lambda)}$ is the positive point charge of the atom s in the unit cell. $\mathbf{r}_s^{(\lambda)}$ is the position vector of atom s in structure λ, which can be decomposed into its components $r_{s,\mu}^{(\lambda)}$ along the crystallographic directions $\hat{\mathbf{e}}_\mu$ as: $\mathbf{r}_s^{(\lambda)} = \sum_{\mu=1}^{3} r_{s,\mu}^{(\lambda)} \hat{\mathbf{e}}_\mu^{(\lambda)} = \sum_{\mu=1}^{3} R_\mu^{(\lambda)} \rho_{s,\mu}^{(\lambda)} \hat{\mathbf{e}}_\mu^{(\lambda)}$, where $R_\mu^{(\lambda)}$ is the lattice constant along μ in direction of unit vector $\hat{\mathbf{e}}_\mu^{(\lambda)}$ of structure λ and $\rho_{s,\mu}^{(\lambda)} = r_{s,\mu}^{(\lambda)} / R_\mu^{(\lambda)}$ is the fractional coordinate of ion s in structure λ along μ-direction. The factor 2 in the numerator of the second term shows the occupation number of states in the valence bands for spin-degenerate systems, n is the band index, $\mathbb{M}$ is the number of occupied bands, $\mathbf{k}$ is the wave vector. The $u_{n,\mathbf{k}}^{(\lambda)}$ are the complex amplitudes or the cell-periodic functions of the Bloch eigenstates $\psi_{n,\mathbf{k}}^{(\lambda)}(\mathbf{r}) = u_{n,\mathbf{k}}^{(\lambda)}(\mathbf{r}) \exp(i\mathbf{k}.\mathbf{r})$ in the structure λ. The amplitudes $u_{n,\mathbf{k}}^{(\lambda)}(\mathbf{r})$ are

cell-periodic so that according to the Bloch's theorem [10] they remain invariant under translation by lattice vector $\mathbf{R}$, *viz.* $u^{(\lambda)}_{n,\mathbf{k}}(\mathbf{r}+\mathbf{R}) = u^{(\lambda)}_{n,\mathbf{k}}(\mathbf{r})$. We have calculated and produced the Bloch functions using the WANNIER90 code [11, 12, 13, 14, 15] and the WIEN2WANNIER code [16], see also [17].

The $-i\nabla_{\mathbf{k}}$ is the momentum representation of the position vector $\mathbf{r}$, and $\langle u^{(\lambda)}_{n,\mathbf{k}}| - i\nabla_{\mathbf{k}}|u^{(\lambda)}_{n,\mathbf{k}}\rangle$ is the electronic contribution of the band n at the wave vector $\mathbf{k}$ in the λ-phase to the Berry phase, *i.e.*, $\varphi^{(\lambda)}_{el,\,n}(\mathbf{k})$, or "Berry connection" or "gauge potential" [7, 18, 19]. The first (second) term in Eq. (1) refers to the ionic (electronic) part of the polarization.

1.1 Berry phase (Bp) approach

The integral in the second term is taken over the first Brillouin zone ($1\mathrm{BZ}^{(\lambda)}$) of the structure λ and gives the electronic Berry phase of the band n for the λ phase, $\varphi^{(\lambda)}_{el,n}$. The summation in the second term is taken over the bands and therefore it is proportional to the electronic Berry phase of the λ structure, *viz.* $\varphi^{(\lambda)}_{el}$. Similarly, the summation in the first term is proportional to the ionic Berry phase of the λ-structure, *i.e.* $\varphi^{(\lambda)}_{ion}$, so that Eq. (1) for polarization in the μ Cartesian direction can be represented as:

$$P^{(\lambda)}_{\mu} = \frac{e}{2\pi}\frac{\varphi^{(\lambda)}_{\mu}}{\Omega^{(\lambda)}}\,R^{(\lambda)}_{\mu}, \tag{2}$$

where $\varphi^{(\lambda)}_{\mu}(=\varphi^{(\lambda)}_{ion}+2\varphi^{(\lambda)}_{el})$ is the total Berry phase corresponding to the μ component of the polarization for the structure λ. The factor of 2 indicates the spin degeneracy for non-spin-polarized systems. For spin-polarized systems we have $\varphi^{(\lambda)}_{\mu} = \varphi^{(\lambda)}_{ion} + \varphi^{(\uparrow),(\lambda)}_{el} + \varphi^{(\downarrow),(\lambda)}_{el}$, where $(\uparrow)$ and $(\downarrow)$ stand for spins up and down, respectively. In Eq. (2), $R^{(\lambda)}_{\mu}$ is the length of the lattice vector in the real space for the structure λ, *viz.* $\mathbf{R}^{(\lambda)} = \sum_{\mu=1}^{3} R^{(\lambda)}_{\mu}\hat{\mathbf{e}}^{(\lambda)}_{\mu}$, where $R^{(\lambda)}_{\mu}\hat{\mathbf{e}}^{(\lambda)}_{\mu}(R^{(\lambda)}_{\mu})$ is the primitive vector (lattice constant, as aforementioned before) of structure λ along μ in the direction of unit vector $\hat{\mathbf{e}}^{(\lambda)}_{\mu}$. Before continuing it is worth noting that the Berry phase is a scalar quantity which has not component like a vector. However, the Berry phase depends on the direction of the polarization. Therefore, the index μ is used in $\varphi^{(\lambda)}_{ion,\mu}$ to indicate that the Berry phase is related to the μ component of the electric polarization vector. In order to abbreviate the phrase, however, let us define this abbreviation "the Berry phase in μ direction" and use it through this paper rather than the long phrase of "the Berry phase corresponding to the μ component of the electric polarization". Let us return to the main discussion. Based on the above definitions, $\varphi^{(\lambda)}_{\mu}$ can be decomposed into its ionic and electronic contributions, *viz.* $\varphi^{(\lambda)}_{\mu} = \varphi^{(\lambda)}_{ion,\mu} + \varphi^{(\lambda)}_{el,\mu}$. From Eqs. (1) and (2), the ionic part of the Berry phase can be represented in terms of the introduced fractional coordinate of ion s in structure λ, *i.e.*, $\rho^{(\lambda)}_{s,\mu} = r^{(\lambda)}_{s,\mu}/R^{(\lambda)}_{\mu}$:

$$\varphi^{(\lambda)}_{ion,\mu} = 2\pi\sum_{s=1}^{N} Z^{(\lambda)}_{s}\rho^{(\lambda)}_{s,\mu}\ . \tag{3}$$

The electronic part of the Berry phase for the band n in $\hat{\mathbf{e}}_{\mu}$ direction $\varphi^{(\lambda)}_{el,n,\mu}$ can be written in this form [20]:

$$\varphi^{(\lambda)}_{el,n,\mu} = -\frac{1}{\Omega_{1BZ}{}^{(\lambda)}}\int_{1\mathrm{BZ}^{(\lambda)}} d\mathbf{k}\left\langle u^{(\lambda)}_{n,\mathbf{k}}\right|\mathbf{G}_{\mu}.i\nabla_{\mathbf{k}}\left|u^{(\lambda)}_{n,\mathbf{k}}\right\rangle, \tag{4}$$

where $\Omega^{(\lambda)}_{1BZ}$ is the unit cell volume of the reciprocal lattice of structure λ, and $\mathbf{G}_{\mu}$ is the primitive reciprocal lattice vector in the $\hat{\mathbf{e}}_{\mu}$ direction, *viz* $\mathbf{G}_{\mu} = G_{\mu}\hat{\mathbf{e}}_{\mu}$. According to Eq. (1), electronic part of the polarization vector of the structure λ for the band n can be represented as:

$$\mathbf{P}^{(\lambda)}_{el,n} = \frac{2e}{(2\pi)^3}\int_{1\mathrm{BZ}^{(\lambda)}} d\mathbf{k}\left\langle u^{(\lambda)}_{n\mathbf{k}}\right| - i\nabla_{\mathbf{k}}\left|u^{(\lambda)}_{n\mathbf{k}}\right\rangle. \tag{5}$$

After multiplying both sides of Eq. (5) by $\mathbf{G}_{\mu}$, we have:

$$\mathbf{G}_{\mu}.\mathbf{P}^{(\lambda)}_{el,n} = -\frac{2e}{(2\pi)^3}\int_{1\mathrm{BZ}^{(\lambda)}} d\mathbf{k}\left\langle u^{(\lambda)}_{n\mathbf{k}}\right|\mathbf{G}_{\mu}.i\nabla_{\mathbf{k}}\left|u_{n\mathbf{k}}{}^{(\lambda)}\right\rangle. \tag{6}$$

By comparing Eqs. (4) and (6), we see that:

$$\mathbf{G}_{\mu}.\mathbf{P}^{(\lambda)}_{el,n} = \frac{2e}{(2\pi)^3}\Omega_{1BZ}{}^{(\lambda)}\ \varphi^{(\lambda)}_{el,n,\mu}. \tag{7}$$

If we recall that $\mathbf{G}_\mu = G_\mu \hat{\mathbf{e}}_\mu$, the inner product in the left hand side of Eq. (7) can be written as $\mathbf{G}_\mu.\mathbf{P}^{(\lambda)}_{el,n} = G_\mu P^{(\lambda)}_{el,n,\mu}$. Then, if we multiply both sides of Eq. (7) by R_μ, it can be read as:

$$R_\mu G_\mu P^{(\lambda)}_{el,n,\mu} = \frac{2e}{(2\pi)^3} \Omega_{1BZ}{}^{(\lambda)}\ \varphi^{(\lambda)}_{el,n,\mu} R_\mu, \tag{8}$$

where R_μ is the length of the real-space primitive lattice vector corresponding to G_μ. By replacing $\Omega^{(\lambda)}_{1BZ} = (2\pi)^3/\Omega^{(\lambda)}$ and considering $R_\mu G_\mu = 2\pi$, Eq. (8) can be simplified as:

$$P^{(\lambda)}_{el,n,\mu} = \frac{2e}{2\pi} \frac{1}{\Omega^{(\lambda)}}\ \varphi^{(\lambda)}_{el,n,\mu} R_\mu. \tag{9}$$

This equation implies that in order to find $P^{(\lambda)}_{el,n,\mu}$ it is enough to obtain $\varphi^{(\lambda)}_{el,n,\mu}$. In principle, the $\varphi^{(\lambda)}_{el,n,\mu}$ can be evaluated by Eq. (4). In practice, however, it is time consuming [2]. To compute $\varphi^{(\lambda)}_{el,n,\mu}$ instead the following procedure which is more practical can be used [2]. First, an appropriate k-points sampling is performed. In the 1BZ of the reciprocal lattice each sample $\mathbf{k}$ is decomposed into two $\mathbf{k}_\parallel$ and $\mathbf{k}_\perp$ samples, *viz.* $\mathbf{k} = \mathbf{k}_\parallel + \mathbf{k}_\perp$, where $\mathbf{k}_\parallel$ ($\mathbf{k}_\perp$) is parallel (perpendicular) to (on) the $\mathbf{G}_\mu$. Thus, $\mathbf{k}_\perp$ samples are 2-dimensional (2D) vectors which form several 2D-sheets. The normal vectors of these 2D-planes are $\mathbf{k}_\parallel$ samples. By this, a suitable arrangement of k-points is formed in the 1BZ for calculating polarization along $\mathbf{k}_\parallel$. Now, the electronic part of the Berry phase of structure λ along the polarization direction $\hat{\mathbf{e}}_\mu$ by considering all the occupied bands, $\varphi^{(\lambda)}_{el,\mu}(\mathbf{k}_\perp) = \Sigma_n \varphi^{(\lambda)}_{el,n,\mu}(\mathbf{k}_\perp)$, can be defined along $\mathbf{k}_\parallel$ over a set of $\mathbb{N}_{\mathbf{k}_\perp}$ discrete points at every $\mathbf{k}_\perp$ point as:

$$\begin{aligned} \varphi^{(\lambda)}_{el,\mu}(\mathbf{k}_\perp) &= 2\sum_{n=1}^{\mathbb{M}} \int_0^{|\mathbf{G}_\parallel|} d\mathbf{k}_\parallel \langle u^{(\lambda)}_{n,\mathbf{k}_\parallel} | -i\nabla_{\mathbf{k}_\parallel} | u^{(\lambda)}_{n,\mathbf{k}_\parallel}\rangle, \\ &= \frac{2}{i}\sum_{n=1}^{\mathbb{M}} \int_{-|\mathbf{G}_\parallel|/2}^{|\mathbf{G}_\parallel|/2} d\mathbf{k}_\parallel \langle u^{(\lambda)}_{n,\mathbf{k}_\parallel} | \nabla_{\mathbf{k}_\parallel} | u^{(\lambda)}_{n,\mathbf{k}_\parallel}\rangle, \end{aligned} \tag{10}$$

where the factor 2 stands for the spin degeneracy, and $\mathbb{M}$ is the number of the occupied bands. The $d\mathbf{k}_\parallel |u^{(\lambda)}_{n,\mathbf{k}_\parallel}\rangle$ can be approximately substituted into Eq. (10) by $d\mathbf{k}_\parallel |\nabla_{\mathbf{k}_\parallel} u^{(\lambda)}_{n,\mathbf{k}_\parallel}\rangle \approx |u^{(\lambda)}_{n,\mathbf{k}_\parallel}\rangle - |u^{(\lambda)}_{n,\mathbf{k}_\parallel + d\mathbf{k}_\parallel}\rangle$, which is nothing more than the Taylor expansion of $|u^{(\lambda)}_{n,\mathbf{k}_\parallel + d\mathbf{k}_\parallel}\rangle$ up to the first order, *viz.* $|u^{(\lambda)}_{n,\mathbf{k}_\parallel + d\mathbf{k}_\parallel}\rangle \approx |u^{(\lambda)}_{n,\mathbf{k}_\parallel}\rangle + d\mathbf{k}_\parallel |\nabla_{\mathbf{k}_\parallel} u^{(\lambda)}_{n,\mathbf{k}_\parallel}\rangle$ [7]. In this case, $\varphi^{(\lambda)}_{el,\mu}(\mathbf{k}_\perp)$, which is wrapped into the phase interval of $[-\pi,\pi]$ [21], can be represented as [2, 7]:

$$\varphi^{(\lambda)}_{el,\mu}(\mathbf{k}_\perp) = \lim_{J\to\infty} 2\Im \left[\ln \left(\prod_{j=0}^{J-1} \left| \mathbb{O}^{(\lambda)}_{\mathbb{M}\times\mathbb{M}}(\mathbf{k}_j, \mathbf{k}_{j+1}) \right| \right) \right], \tag{11}$$

where $\Im$ stands for the "imaginary part of", and $\left|\mathbb{O}^{(\lambda)}_{\mathbb{M}\times\mathbb{M}}(\mathbf{k}_j, \mathbf{k}_{j+1})\right|$ is the determinant of the overlap matrix $\mathbb{O}^{(\lambda)}_{\mathbb{M}\times\mathbb{M}}(\mathbf{k}_j, \mathbf{k}_{j+1}) = \left\langle u^{(\lambda)}_{n,\mathbf{k}_j} \middle| u^{(\lambda)}_{n,\mathbf{k}_{j+1}} \right\rangle$ that is calculated by the inner product of the Bloch wavefunctions $u^{(\lambda)}_{n,\mathbf{k}_j} (= e^{-i\mathbf{G}_\parallel \cdot \mathbf{r}} u^{(\lambda)}_{n,\mathbf{k}_0})$ and $u^{(\lambda)}_{n,\mathbf{k}_{j+1}}$. Here, $n(\in [1,\mathbb{M}])$ is the band index. The wave vector in the reciprocal space $\mathbf{k}_j = \mathbf{k}_s + \frac{j}{J}\mathbf{G}_\parallel = -\mathbf{G}_\parallel/2 + \frac{j}{J}\mathbf{G}_\parallel = \frac{2j-J}{J}\mathbf{G}_\parallel/2$ is the j-th k-point in the 1BZ where $\mathbf{k}_s = -\mathbf{G}_\parallel/2$ is the starting $\mathbf{k}$-point locating at the bottom surface of the 1BZ, j runs through a set of J discrete points from $j=0$ to $j=J-1$ along the polarization direction $\hat{\mathbf{e}}_\mu$ and $\mathbf{G}_\parallel$ is the shortest reciprocal vector parallel to $\hat{\mathbf{e}}_\mu$, see Fig. 2 of the main text. If $j=0$ ($j=J$), then $\mathbf{k}_j$ is located at the bottom (top) surface of the 1BZ, *viz.* $\mathbf{k}_{j=0} = \frac{0-J}{J}\frac{\mathbf{G}_\parallel}{2} = -\mathbf{G}_\parallel/2$ ($\mathbf{k}_{j=J} = \frac{2J-J}{J}\frac{\mathbf{G}_\parallel}{2} = +\mathbf{G}_\parallel/2$), see Fig. 2 of the main text. However, if the bottom surface of the 1BZ is meshed by the k-points inside the 1BZ, the top surface of the 1BZ will be automatically meshed by the k-points inside the next BZ located on top of the 1BZ due to the periodic boundary conditions. Namely, the top surface of the 1BZ is identical to the bottom surface of the next unit cell located on top of the initial 1BZ. Thus, the upper limit in Eq. (11) is set to $j=J-1$. If $j=J-1$, then $\mathbf{k}_j$ is located at one discreet step, i.e. $\mathbf{G}_\parallel/J$, before the top surface of the 1BZ, *viz.* $\mathbf{k}_{j=J-1} = \frac{2J-2-J}{J}\frac{\mathbf{G}_\parallel}{2} = +\mathbf{G}_\parallel/2 - \mathbf{G}_\parallel/J$, see Fig. 2 of main text. The starting $\mathbf{k}$ point, $\mathbf{k}_s$, belongs to both a string parallel to the $\hat{\mathbf{e}}_\mu$ and the surface perpendicular to the $\hat{\mathbf{e}}_\mu$, see Fig. 2 of the main text. Thus, the $\mathbf{k}_s$ plays a dual role which can be either $-\mathbf{G}_\parallel/2$ or $\mathbf{k}_\perp$, see Fig. 2 of the main text. The $\mathbf{k}_s$ is $-\mathbf{G}_\parallel/2$ when we are working with the right-hand side of Eq. (11) considering the $\mathbf{k}$ mesh distributed over the strings parallel to the polarization direction $\hat{\mathbf{e}}_\mu$, while $\mathbf{k}_s$ is $\mathbf{k}_\perp$ when we are working with the left-hand side of Eq. (11) considering the $\mathbf{k}$ mesh distributed over the surface perpendicular to the polarization direction $\hat{\mathbf{e}}_\mu$.

Then, the electronic Berry phase can be calculated by taking an average over the perpendicular area $A_\perp$ or numerically over the discrete points $\mathbb{N}_{\mathbf{k}_\perp}$ of the 2D $\mathbf{k}_\perp$-point samples as:

$$\begin{aligned} \varphi^{(\lambda)}_{el,\mu} &= \frac{1}{A_\perp}\int_{A_\perp} dA_\perp \varphi^{(\lambda)}_{el,\mu}(\mathbf{k}_\perp) \\ &\approx \frac{1}{\mathbb{N}_{k_\perp}}\sum_{\mathbf{k}_\perp}\varphi^{(\lambda)}_{el,\mu}(\mathbf{k}_\perp), \end{aligned} \tag{12}$$

where $\mathbf{k}_\perp$ points distributed over the perpendicular 2D area $A_\perp$ are nothing more than the $\mathbf{k}_s$ points, as discussed their double roles above, distributed along the parallel 1D strings. Now, we have straightforwardly obtained the total Berry phase as $\varphi^{(\lambda)}_{\mu} = \varphi^{(\lambda)}_{el,\mu} + \varphi^{(\lambda)}_{ion,\mu}$ for the structures "$\lambda = 0$" and "$\lambda = 1$" individually. By plugging $\varphi^{(\lambda)}_{\mu}$ into Eq. (2), we have then obtained the value of the electric polarization $P^{(\lambda)}_{\mu}$ and as a result the polarization vector $\mathbf{P}^{(\lambda)} = P^{(\lambda)}_{3}\hat{\mathbf{e}}_3$ for the structures "$\lambda = 0$" and "$\lambda = 1$" individually. Ultimately, employing the modern theory of polarization [1, 2, 3, 4, 5, 6, 7, 8, 9], we have calculated the spontaneous polarization $\Delta\mathbf{P}$ as $\Delta\mathbf{P} = \mathbf{P}^{(\lambda=1)} - \mathbf{P}^{(\lambda=0)}$, where $\mathbf{P}^{(\lambda=0)}$ refers to the polarization of the non-polar CS structure having higher-symmetry structure while $\mathbf{P}^{(\lambda=1)}$ refers to the polar noncentrosymmetric (NCS) structure having lower-symmetry, [2, 7, 9]. For the compound in question, the non-polar centrosymmetric (CS) is R$\bar{3}$c and the polar NCS is R3c.

It is worth recalling that polarization can generally occurs in three dimensions (3D). Thus, μ generally can be 1, 2, and 3 in Eq. (11). However, we have transformed the crystal system of $LiOsO_3$ from the rhombohedral (trigonal) to the hexagonal structure, as discussed in Sec. 5. By this transformation, we have oriented the polarization direction towards a single direction along the c-axis of the hexagonal structure, as discussed in "SEP direction in $LiOsO_3$ FE-LM" of the main text. Therefore, for our case the μ is set to 3 in Eq. (11). Thus, for our case $\mathbf{k}_j = \frac{2j-J}{J}\frac{\mathbf{G}_\parallel}{2}$ can be simplified as $\mathbf{k}_j = \frac{2j-J}{J}\frac{\pi}{c}\hat{\mathbf{e}}_3$, where c is the lattice constant along $\hat{\mathbf{e}}_3$, and $\mathbf{G}_\parallel$ is $\frac{2\pi}{c}\hat{\mathbf{e}}_3$, see Fig. 2 of the main text.

1.2 Wannier functions (Wf) approach

Before starting it is worth to highlight that a group of isolated bands differs from an isolated group of bands or composite bands. A group of bands is defined [13, 14, 15, 22] to be isolated if it is only well separated by finite gaps from all other higher and lower bands in the whole of the BZ. We define a group of isolated bands to be not only well separated by finite gaps from all other higher and lower bands in the whole of the BZ but also no band of the group can show degeneracies and cross any other bands as well as hybridize with itself. The composite bands, as defined in Ref. [13], are identical to an isolated group of bands, as defined in Ref. [13] and indicated in Refs.[14, 15, 22], while differ from a group of isolated bands, as defined above in this work. A group of isolated bands is also an isolated group of bands. However, an isolated group of bands may not be necessarily a group of isolated bands, because bands crossing and degeneracies as well as hybridizations within itself are arbitrary (forbidden) in the former (latter) group. Crystals having a group of isolated bands are ideal as can be hardly found in nature. This is why the localized Wannier functions method [2, 23] has been generalized to the maximally localized Wannier functions method [13, 14, 15, 22]. Let us begin the main discussion below.

Gregory H. Wannier devised a transformation between atomic-like localized functions and the Bloch wavefunctions of ideal insulators containing one electron in the lowest excited state taken out of a full band of several electrons to investigate the energy spectra of the ideal insulators [23]. Since then, this idea has been called the localized Wannier functions method. The tight-binding [10] and some of the many-body [24] Hamiltonians can be related to the localized Wannier functions. R. D. King-Smith and David Vanderbilt [2] applying the localized Wannier functions formulated for a single isolated band developed the theory of polarization of crystalline solids.

In the Wannier functions approach, the ionic part of the polarization for structure λ, with no need to connect it to the Berry phase via Eq. (3), can be directly evaluated by the first term to the right-hand side of Eq. (1) as [1, 2, 3, 4, 5, 6, 7, 8, 9]:

$$\mathbf{P}^{(\lambda)}_{ion} = \frac{e}{\Omega^{(\lambda)}}\sum_{s=1}^{\mathbb{N}} Z^{(\lambda)}_s \mathbf{r}^{(\lambda)}_s, \tag{13}$$

In this approach, the electronic part of polarization, as given by the second term to the right-hand side of Eq. (1), can be simplified [2]. To this end, the cell-periodic functions $u^{(\lambda)}_{n,\mathbf{k}}(\mathbf{r})$ of the Bloch eigenstates, $\psi^{(\lambda)}_{n,\mathbf{k}}(\mathbf{r}) = u^{(\lambda)}_{n,\mathbf{k}}(\mathbf{r})\exp(i\mathbf{k}.\mathbf{r})$, for the structure λ can be expanded in terms of Wannier functions $W^{(\lambda)}_n(\mathbf{r}-\mathbf{R})$ of the occupied bands n as follows:

$$u^{(\lambda)}_{n,\mathbf{k}}(\mathbf{r}) = \frac{1}{\sqrt{N}}\sum_{\mathbf{R}}\exp\left(i\mathbf{k}.(\mathbf{r}-\mathbf{R})\right)W^{(\lambda)}_n(\mathbf{r}-\mathbf{R}), \tag{14}$$

where N is the number of unit cell in the crystal and $W_n^{(\lambda)}(\mathbf{r}-\mathbf{R})$ for the group of isolated bands, as defined in this subsection, can be defined as [2, 10]:

$$
\begin{aligned}
W_n^{(\lambda)}(\mathbf{r}-\mathbf{R}) &:= \mathscr{W}_{n,\mathbf{R}}^{(\lambda)}(\mathbf{r}) \\
&:= \frac{\sqrt{N}\Omega^{(\lambda)}}{(2\pi)^3}\int_{1\mathrm{BZ}^{(\lambda)}} d\mathbf{k}\exp\left(-i\mathbf{k}.(\mathbf{r}-\mathbf{R})\right)u_{n,\mathbf{k}}^{(\lambda)}(\mathbf{r}),
\end{aligned}
\tag{15}
$$

where $\mathscr{W}$ symbol in $\mathscr{W}_{n,\mathbf{R}}^{(\lambda)}(\mathbf{r})$ standing for the localized Wannier functions is used to avoid conflicting with its generalized maximally localized form W, as to be defined in Eq. (22). The Eq. (15) is generalized to Eq. (22) in Ref. [13] to obtain the maximally localized Wannier functions. As can be seen from Eq. (15), in the group of isolated bands, the bands are not mixed to each other. The Wannier functions and Bloch wavefunctions are labeled by the same band index n. This indicates, in agreement with Ref. [13], that each Wannier function like each Bloch wavefunction can be corresponded only to a single band n. There is a one to one correspondence between a single band n and a single localized Wannier function $\mathscr{W}_{n,\mathbf{R}}^{(\lambda)}(\mathbf{r})$ indexed by the same subscript n. Eq. (15) implies that a single localized Wannier function $W_{n,\mathbf{R}}^{(\lambda)}(\mathbf{r})$ indexed by the subscript n can contribute only to a single isolated band with the same index n. This may not be the case for an isolated group of bands as to be discussed subsequently.

By substituting Eq. (14) into Eq. (1), the electronic part of polarization can be also directly evaluated in real space as [25]:

$$
\begin{aligned}
\mathbf{P}_{el}^{(\lambda)} &= \frac{2e}{\Omega^{(\lambda)}}\sum_{n=1}^{\mathbb{M}}\langle\mathscr{W}_{n,\mathbf{R}}^{(\lambda)}|\,\mathbf{r}\,|\mathscr{W}_{n,\mathbf{R}}^{(\lambda)}\rangle \\
&= \frac{2e}{\Omega^{(\lambda)}}\sum_{n=1}^{\mathbb{M}}\iint d\mathbf{r}'d\mathbf{r}''\langle\mathscr{W}_{n,\mathbf{R}}^{(\lambda)}|\mathbf{r}'\rangle\langle\mathbf{r}'|\,\mathbf{r}\,|\mathbf{r}''\rangle\langle\mathbf{r}''|\,\mathscr{W}_{n,\mathbf{R}}^{(\lambda)}\rangle \\
&= \frac{2e}{\Omega^{(\lambda)}}\sum_{n=1}^{\mathbb{M}}\iint d\mathbf{r}'d\mathbf{r}''\mathscr{W}_{n,\mathbf{R}}^{*(\lambda)}(\mathbf{r}')\mathbf{r}''\langle\mathbf{r}'|\mathbf{r}''\rangle\mathscr{W}_{n,\mathbf{R}}^{(\lambda)}(\mathbf{r}'') \\
&= \frac{2e}{\Omega^{(\lambda)}}\sum_{n=1}^{\mathbb{M}}\iint d\mathbf{r}'d\mathbf{r}''\mathscr{W}_{n,\mathbf{R}}^{*(\lambda)}(\mathbf{r}')\mathbf{r}''\delta(\mathbf{r}'-\mathbf{r}'')\mathscr{W}_{n,\mathbf{R}}^{(\lambda)}(\mathbf{r}'') \\
&= \frac{2e}{\Omega^{(\lambda)}}\sum_{n=1}^{\mathbb{M}}\int d\mathbf{r}'\mathscr{W}_{n,\mathbf{R}}^{*(\lambda)}(\mathbf{r}')\mathbf{r}'\mathscr{W}_{n,\mathbf{R}}^{(\lambda)}(\mathbf{r}') \\
&= \frac{2e}{\Omega^{(\lambda)}}\sum_{n=1}^{\mathbb{M}}\int \mathbf{r}\left|\mathscr{W}_{n,\mathbf{R}}^{(\lambda)}(\mathbf{r})\right|^2 d\mathbf{r},
\end{aligned}
\tag{16}
$$

where the dummy variable $\mathbf{r}'$ is changed to $\mathbf{r}$ in the last integral which can be more compactly written as:

$$
\mathbf{P}_{el}^{(\lambda)} = \frac{e}{\Omega^{(\lambda)}}\sum_{n=1}^{\mathbb{M}} 2\langle\mathbf{r}\rangle_{\mathscr{W}_{n,\mathbf{R}}^{(\lambda)}}. \tag{17}
$$

The total polarization per unit volume of structure λ can be by combining Eqs. (13) and (17) represented as:

$$
\mathbf{P}^{(\lambda)} = \mathbf{P}_{ion}^{(\lambda)} + \mathbf{P}_{el}^{(\lambda)} = \frac{e}{\Omega^{(\lambda)}}\left[\sum_{s=1}^{\mathbb{N}} Z_s^{(\lambda)}\mathbf{r}_s^{(\lambda)} + \sum_{n=1}^{\mathbb{M}} 2\langle\mathbf{r}\rangle_{\mathscr{W}_{n,\mathbf{R}}^{(\lambda)}}\right]. \tag{18}
$$

This equation can be applied on systems having groups of isolated bands as used in Ref. [2] and defined as well as extended to the isolated groups of bands in Ref. [13, 14], which the latter is reviewed and redefined also in [15, 22]. In Eqs. (16), (16) and (18), $\langle\mathbf{r}\rangle_{\mathscr{W}_{n,\mathbf{R}}^{(\lambda)}} = \langle\mathscr{W}_{n,\mathbf{R}}^{(\lambda)}|\,\mathbf{r}\,|\mathscr{W}_{n,\mathbf{R}}^{(\lambda)}\rangle = \int \mathbf{r}|\mathscr{W}_{n,\mathbf{R}}^{(\lambda)}(\mathbf{r})|^2 d\mathbf{r}$ is the center of charge of the n-th localized Wannier function in structure λ. Eq. (17) implies that $\Delta\mathbf{P}_{el} = \mathbf{P}_{el}^{(\lambda=1)} - \mathbf{P}_{el}^{(\lambda=0)}$ is proportional to the displacement of the center of charge of the Wannier functions due to the adiabatic phase transition [2]. Although the form of Eq. (17) has become very similar to Eq. (13), it would be noted that the latter is a classical equation while the former is a quantum mechanical equation where the expectation value of $\langle\mathbf{r}\rangle_{\mathscr{W}_{n,\mathbf{R}}^{(\lambda)}}$ should be evaluated quantum mechanically under the Wannier functions. In special cases, however, the second term in Eq. (18), i.e. the electronic polarization, can really reduce to a form as simple as the first term in Eq. (18), i.e. the ionic part of the polarization. If the structure "$\lambda = 0$" is identical with structure "$\lambda = 1$" that is if

the Hamiltonian of the system remains unchanged, i.e., $H^{\lambda=0} = H^{\lambda=1}$, under the adiabatic transition, then the electronic part of polarization can be also easily evaluated in reciprocal space with no need to transform it to Eqs. (11) and (12). This can occur during an adiabatic transition, for instance, in a closed path or in paths corresponding to the translation of the crystal by the periodic translations of $\mathbf{r} \longrightarrow \mathbf{r} + \lambda\mathbf{R}$. In this case, the initial and final cell-periodic functions can differ by a maximum of a periodic phase factor [2], *viz.* $u_{n,\mathbf{k}}^{(\lambda=1)}(\mathbf{r}) = e^{i\theta_{n,\mathbf{k}}} u_{n,\mathbf{k}}^{(\lambda=0)}(\mathbf{r})$. Therefore, for such special cases, the change in electronic polarization is quantized as:

$$\Delta\mathbf{P}_{el}^{(\lambda)} = \frac{e}{\Omega^{(\lambda)}} \sum_{n=1}^{\mathbb{M}} 2\mathbf{R}_n^{(\lambda)} = \mathbb{M}\left(\frac{2e}{\Omega^{(\lambda)}}\mathbf{R}^{(\lambda)}\right), \tag{19}$$

where $e\mathbf{R}^{(\lambda)}/\Omega^{(\lambda)}$ is the quantum of the electronic polarization counted by a natural number of the occupied bands $\mathbb{M}$. Eq. (19) implies that the electronic polarization can only change by an integer multiple of this quantum of polarization for an adiabatic change in the Hamiltonian for which $H^{(\lambda=0)} = H^{(\lambda=1)}$ [2]. An integer or a natural multiple of the quantum of polarization is identical to the zero polarization. However, this does not hold for our case where the Hamiltonian of the $LiOsO_3$ changes from the non-polar CS R$\bar{3}$c to the polar NCS R3c, *viz.* $H^{(\lambda=0\equiv R\bar{3}c)} \neq H^{(\lambda=1\equiv R3c)}$. Therefore, in place of the special Eq. (19), one would consider Eq. (16) or (17) which can be applied on insulators having groups of isolated and occupied bands. However, even Eqs. (16) and (17) are neither applicable for metals having partially occupied bands nor insulators lacking groups of isolated bands. Let us close the isolated single band discussion by recalling that the Wannier functions are not unique which leads to the freedom in the choice of the following phase $\theta_{n,\mathbf{k}}$:

$$u_{n,\mathbf{k}}^{(\lambda)}(\mathbf{r}) \longrightarrow e^{i\theta_{n,\mathbf{k}}} u_{n,\mathbf{k}}^{(\lambda)}(\mathbf{r}). \tag{20}$$

Nicola Marzari and David Vanderbilt [13] extended the localized Wannier functions method so that it can be applicable not only for special cases having groups of isolated bands but also for more general cases having an isolated group or isolated groups of bands (composite bands), see the bands of classes I and II in Fig. 1 of the main text as two isolated groups of bands. This extension is the popular maximally localized Wannier functions method. The system under study has two classes of isolated bands which are well separated from each other but the bands cross each other, see Fig. 1 of the main text and PBE-GGA results presented in Fig. SM1. In this extended approach, in place of the periodic gauge transformation given in Eq. (20), the following gauge for the composite bands is proposed:

$$u_{n,\mathbf{k}}^{(\lambda)}(\mathbf{r}) \longrightarrow \sum_{m=1}^{\mathbb{J}} U_{mn}^{(\mathbf{k}),(\lambda)} u_{m,\mathbf{k}}^{(\lambda)}(\mathbf{r}), \tag{21}$$

where $\mathbb{J}$ is the number of composite bands and $U_{mn}^{(\mathbf{k}),(\lambda)}$ are matrix elements of the unitary matrix U at point $\mathbf{k}$ for structure λ. It is important to note that, in contrast to the periodic gauge introduced in Eq. (20), $U_{mn}^{(\mathbf{k}),(\lambda)}$ can mix the bands due to the summation over the number of composite bands. Eq. (21) reduces to Eq. (20) when U is diagonal, as expected. By inserting Eq. (21) into Eq. (15), the Wannier orbitals in unit cell R of structure λ is generalized as:

$$\begin{aligned} &W_n^{(\lambda)}(\mathbf{r}-\mathbf{R}) := W_{n,\mathbf{R}}^{(\lambda)}(\mathbf{r}) \\ &:= \frac{\sqrt{N}\Omega^{(\lambda)}}{(2\pi)^3} \int_{1\mathrm{BZ}^{(\lambda)}} d\mathbf{k} \left[e^{-i\mathbf{k}.(\mathbf{r}-\mathbf{R})} \sum_{m=1}^{\mathbb{J}} U_{mn}^{(\mathbf{k}),(\lambda)} u_{m,\mathbf{k}}^{(\lambda)}(\mathbf{r}) \right], \end{aligned} \tag{22}$$

where index n in $W_{n,\mathbf{R}}^{(\lambda)}(\mathbf{r})$, in contrast to $\mathscr{W}_{n,\mathbf{R}}^{(\lambda)}(\mathbf{r})$ expressed in Eq. (15), cannot be interpreted as the usual band index. In the right-hand-side of this equation the summation is taken over the number of bands of the selected isolated group of bands. Therefore, the bands are mixed so that $W_{n,\mathbf{R}}^{(\lambda)}(\mathbf{r})$ cannot be necessarily attributed to a single band only. In fact, here, n (m) in Eq. (22) is an index to count the number of maximally localized Wannier functions (composite bands). The number of composite bands $\mathbb{J}$ can be equal to the number of maximally localized Wannier functions, namely m and n can vary from 1 to $\mathbb{J}$. However, there may not be a one to one correspondence between a single band indexed by m and a single maximally localized Wannier function $W_{n,\mathbf{R}}^{(\lambda)}(\mathbf{r})$ indexed by subscript n. In other words, Eq. (22) implies that several various bands with different m indexes can contribute to a single maximally localized Wannier function $W_{n,\mathbf{R}}^{(\lambda)}(\mathbf{r})$ indexed by only one subscript n.

Ivo Souza, Nicola Marzari, and David Vanderbilt [14] generalized the maximally localized Wannier functions method considering more general cases lacking even composite bands, see figure 1 of Ref. [14] where 5 narrow d bands of copper are entangled to s wide band. This is the so-called generalized maximally localized Wannier functions method. The idea of this generalized method is to disentangle the narrow bands from the wide band(s).

To find the set of maximally localized Wannier functions, corresponding to an isolated group of bands, the sum of the quadratic spreads of the Wannier probability distributions $|W_{n,\mathbf{R}}^{(\lambda)}(\mathbf{r})|^2$ is proposed by Marzari and Vanderbilt [13] to be minimized with respect to the unitary matrix $U^{(\lambda)}$:

$$\mathscr{S}_{\mathbf{R}}^{(\lambda)}[U^{(\lambda)}] = \sum_{n=1}^{\mathbb{J}} \left[\langle \mathbf{r^2} \rangle_{W_{n,\mathbf{R}}^{(\lambda)}} - \langle \mathbf{r} \rangle^2_{W_{n,\mathbf{R}}^{(\lambda)}} \right], \tag{23}$$

where $\mathscr{S}_{\mathbf{R}}^{(\lambda)}[U^{(\lambda)}]$ is a localization functional measuring the total delocalization or spread of the $\mathbb{J}$ Wannier functions in the unit cell R of the structure λ around their centers. According to the approach proposed in Ref. [13] and used in Ref. [14] including additional new physical and practical aspects regarding disentanglement procedure, the variation of the localization functional with respect to $U^{(\lambda)}$ is set to zero:

$$\frac{\delta\left(\mathscr{S}_{\mathbf{R}}^{(\lambda)}[U^{(\lambda)}]\right)}{\delta\left(U^{(\lambda)}\right)} = 0 \xrightarrow{\text{yields}} \begin{cases} U_{\text{min}}^{(\lambda)}, \\ \left(\mathscr{S}_{\mathbf{R}}^{(\lambda)}[U_{\text{min}}^{(\lambda)}]\right)_{\text{min}}, \\ \left\{W_{n,\mathbf{R}}^{(\lambda)}(\mathbf{r})\right\}_{\text{Maximally Localized}}, \end{cases} \tag{24}$$

which gives the desired set of maximally localized functions. In practice, the minimization and disentanglement procedures are performed self-consistently. Therefore, the procedures need an initial guess to begin. Hence, it would be more efficiently converged, if the initial guess is shrewd enough. In this way, the problem of calculating the gradient of the spread function requires to calculate the following overlap integral:

$$M_{mn}^{(\mathbf{k},\mathbf{b}),(\lambda)} = \langle u_{m,\mathbf{k}}^{(\lambda)} | u_{n,\mathbf{k+b}}^{(\lambda)} \rangle = \langle \psi_{m,\mathbf{k}}^{(\lambda)} | e^{i\mathbf{b}.\mathbf{r}} | \psi_{n,\mathbf{k+b}}^{(\lambda)} \rangle, \tag{25}$$

where each vector $\mathbf{k}$ is connected to its nearest neighbors by vectors $\mathbf{b}$. To this end, a set of initial trial orbitals g_n is chosen. These g_n are Gussian functions centered at or near midbond positions. Then, the projector operator $\sum_m |\psi_{m,\mathbf{k}}^{(\lambda)}\rangle \langle \psi_{m,\mathbf{k}}^{(\lambda)}|$ is applied on g_n as follows:

$$|\mathrm{g}_{n,\mathbf{k}}^{(\lambda)}\rangle = \sum_m |\psi_{m,\mathbf{k}}^{(\lambda)}\rangle \langle \psi_{m,\mathbf{k}}^{(\lambda)}| g_n\rangle, \tag{26}$$

where $|\mathrm{g}_{n,\mathbf{k}}^{(\lambda)}\rangle$ are the initial trial orbitals g_n projected onto Bloch states of a set of bands at wave vector $\mathbf{k}$ for structure λ. The $|\mathrm{g}_{n,\mathbf{k}}^{(\lambda)}\rangle$ are orthonormalized as follows:

$$|\tilde{\mathrm{g}}_{n,\mathbf{k}}^{(\lambda)}\rangle = \sum_m \frac{|\mathrm{g}_{n,\mathbf{k}}^{(\lambda)}\rangle}{\sqrt{\langle \mathrm{g}_{m,\mathbf{k}}^{(\lambda)} | \mathrm{g}_{n,\mathbf{k}}^{(\lambda)}\rangle}}. \tag{27}$$

Using these orthonormalized projected initial trial orbitals $|\tilde{\mathrm{g}}_{n,\mathbf{k}}^{(\lambda)}\rangle$, the initial cell-periodic functions are produced as follows:

$$u_{n,\mathbf{k}}^{(0),(\lambda)}(\mathbf{r}) = e^{-i\mathbf{k}.\mathbf{r}} \tilde{\mathrm{g}}_{n,\mathbf{k}}^{(\lambda)}(\mathbf{r}). \tag{28}$$

Using these initial cell-periodic functions, the initial elements of the overlap matrix and the cell-periodic functions are generated, receptively, as follows:

$$M_{mn}^{(0),(\mathbf{k},\mathbf{b}),(\lambda)} = \langle u_{m,\mathbf{k}}^{(0),(\lambda)} | u_{n,\mathbf{k+b}}^{(0),(\lambda)} \rangle, \tag{29}$$

$$|u_{n,\mathbf{k}}^{(\lambda)}\rangle = \textstyle\sum_m U_{mn}^{(\mathbf{k}),(\lambda)} |u_{m,\mathbf{k}}^{(0),(\lambda)}\rangle, \tag{30}$$

where $U^{(\mathbf{k}),(\lambda)}$ is initially set to identity matrix, i.e., $U_{mn}^{(\mathbf{k}),(\lambda)} = \delta_{mn}$. The $U_{mn}^{(\mathbf{k}),(\lambda)}$ are updated self-consistently during the steepest-descent procedure to minimize the localization function as follows:

$$U^{(\mathbf{k}),(\lambda)} \longrightarrow U^{(\mathbf{k}),(\lambda)} e^{\Delta W^{(\mathbf{k}),(\lambda)}}, \tag{31}$$

where $\Delta W^{(\mathbf{k}),(\lambda)}$ is a finite form of the infinitesimal antiunitary matrix $dW^{(\mathbf{k}),(\lambda)}$, i.e. $dW^{\dagger(\mathbf{k}),(\lambda)} = -dW^{(\mathbf{k}),(\lambda)}$, see equation (57) of Ref. [13]. The $M_{mn}^{(0),(\mathbf{k},\mathbf{b}),(\lambda)}$ are also updated iteratively as follows:

$$M_{mn}^{(0),(\mathbf{k},\mathbf{b}),(\lambda)} \longrightarrow M_{mn}^{(\mathbf{k},\mathbf{b}),(\lambda)} = U^{\dagger(\mathbf{k}),(\lambda)} M_{mn}^{(0),(\mathbf{k},\mathbf{b}),(\lambda)} U^{(\mathbf{k}),(\lambda)}. \tag{32}$$

The above procedure is self-consistently repeated until reaching a desired convergence. We follow the standard minimization procedure, as proposed in Ref. [13], and the disentanglement procedure, as proposed in [14]. To this end, we use Wannier90 code [11, 12, 13, 14, 15] as interfaced to the WIEN2k [26, 27] by the WIEN2WANNIER code [16] where the matrix elements $M_{mn}^{(0),(\mathbf{k},\mathbf{b}),(\lambda)}$, as expressed in Eq. (25), and the coefficients $\langle \psi_{m,\mathbf{k}}^{(\lambda)} | g_n \rangle$, as expressed in Eq. (26), are reformulated and evaluated in the LAPW bases [28, 29, 30].

Let us by replacing $\mathscr{W}_{n,\mathbf{R}}^{(\lambda)}$, as given by Eq. (22) and appeared in Eqs. (16) to (18), with $W_{n,\mathbf{R}}^{(\lambda)}$, as expressed by Eq. (22), extend the electronic polarization formulae from the framework of the localized Wannier functions [2] to that of the more advanced maximally localized Wannier functions [13, 14], respectively, as follows:

$$\mathbf{P}_{el}^{(\lambda)} = \frac{2e}{\Omega^{(\lambda)}} \sum_{n=1}^{\mathbb{J}} \int \mathbf{r} \left| W_{n,\mathbf{R}}^{(\lambda)}(\mathbf{r}) \right|^2 d\mathbf{r}, \tag{33}$$

$$\mathbf{P}_{el}^{(\lambda)} = \frac{e}{\Omega^{(\lambda)}} \sum_{n=1}^{\mathbb{J}} 2 \langle \mathbf{r} \rangle_{W_{n,\mathbf{R}}^{(\lambda)}}, \tag{34}$$

$$\mathbf{P}^{(\lambda)} = \mathbf{P}_{ion}^{(\lambda)} + \mathbf{P}_{el}^{(\lambda)} = \frac{e}{\Omega^{(\lambda)}} \left[\sum_{s=1}^{\mathbb{N}} Z_s^{(\lambda)} \mathbf{r}_s^{(\lambda)} + \sum_{n=1}^{\mathbb{J}} 2 \langle \mathbf{r} \rangle_{W_{n,\mathbf{R}}^{(\lambda)}} \right], \tag{35}$$

where the number of occupied bands $\mathbb{M}$ is also replaced by the number of composite bands $\mathbb{J}$. In analogy to $\langle \mathbf{r} \rangle_{\mathscr{W}_{n,\mathbf{R}}^{(\lambda)}} = \langle \mathscr{W}_{n,\mathbf{R}}^{(\lambda)} | \mathbf{r} | \mathscr{W}_{n,\mathbf{R}}^{(\lambda)} \rangle = \int \mathbf{r} |\mathscr{W}_{n,\mathbf{R}}^{(\lambda)}(\mathbf{r})|^2 d\mathbf{r}$ in Eqs. (16), (17) and (18), in Eqs. (33), (34) and (35), $\langle \mathbf{r} \rangle_{W_{n,\mathbf{R}}^{(\lambda)}} = \langle W_{n,\mathbf{R}}^{(\lambda)} | \mathbf{r} | W_{n,\mathbf{R}}^{(\lambda)} \rangle = \int \mathbf{r} |W_{n,\mathbf{R}}^{(\lambda)}(\mathbf{r})|^2 d\mathbf{r}$ is also the center of charge of the n-th maximally localized Wannier function in the home unit cell $\mathbf{R}$ of the structure λ. Eq. (34) which is analogous to Eq. (17) similarly implies that $\Delta \mathbf{P}_{el} = \mathbf{P}_{el}^{(\lambda=1)} - \mathbf{P}_{el}^{(\lambda=0)}$ is proportional to the displacement of the center of charge of the maximally Wannier functions due to the adiabatic phase transition [22]. Although the form of the second term of Eq. (35) has become very similar to that of the first term of Eq. (35), the ionic polarization is calculated classically while the electronic polarization is calculated quantum mechanically as $\langle \mathbf{r} \rangle_{W_{n,\mathbf{R}}^{(\lambda)}}$ under the maximally localized state $W_{n,\mathbf{R}}^{(\lambda)}$.

K. S. Thygesen, L. B. Hansen, and K. W. Jacobsen generalized the fully occupied Wannier functions to partly occupied Wannier functions [31, 32]. It is worth noting that ”partly occupied” differs from ”partially occupied”. For example, ”partly occupied maximally localized Wannier functions” refer to those Wannier functions that are made maximally localized by considering both the fully occupied valence bands and empty low-lying conduction bands, instead of considering fully occupied valence bands only, see Refs. [31, 32, 33]. The ”partially occupied state” refers to a state that is not fully occupied. By including low-lying empty conduction states to the fully occupied valence states the resulting states can be partially occupied, if the electrons distributed over the valence states only are allowed to be redistributed over the valence and the added conduction states, as explored by the partly occupied Wannier functions method to reduce the spreads and as a result enhance the localization degrees of the produced maximally localized Wannier functions [31, 32, 33]. The ”partially occupied band” can be also associated to a band that crosses the Fermi level. In this case, a partially occupied band can belong to both the valence and conduction regions in a metal.

In analogy to the extension made from Eq. (20), as the gauge transformation compatible with the Wannier functions of the group of isolated bands, to Eq. (21), as the more general gauge transformation suitable for the Wannier functions of the isolated group of bands, they [31, 32] furthermore generalized the latter gauge transformation given in Eq. (21) to the following transformation which is applicable for the partly occupied Wannier functions:

$$u_{n,\mathbf{k}}^{(\lambda)}(\mathbf{r}) \longrightarrow \sum_{m=1}^{\mathbb{J}^{(\mathrm{II})}} U_{mn}^{(\mathbf{k}),(\lambda)} u_{m,\mathbf{k}}^{(\lambda),(\mathrm{II})}(\mathbf{r}) + \sum_{m=\mathbb{J}^{(\mathrm{II})}+1}^{\mathbb{J}^{(\mathrm{II})}+\mathbb{J}^{(\mathrm{I})}} U_{mn}^{(\mathbf{k}),(\lambda)} \mathscr{U}_{m,\mathbf{k}}^{(\lambda),(\mathrm{I})}(\mathbf{r}), \tag{36}$$

where II (I) stands for the fully occupied (empty) valence (conduction) part of the bands and whence $\mathbb{J}^{(\mathrm{II})}$ ($\mathbb{J}^{(\mathrm{I})}$) is the number of occupied (empty) valence (low-lying conduction) bands in region II (I). In the second term of Eq. (36), $\mathscr{U}_{m,\mathbf{k}}^{(\lambda),(\mathrm{I})}(\mathbf{r})$ is

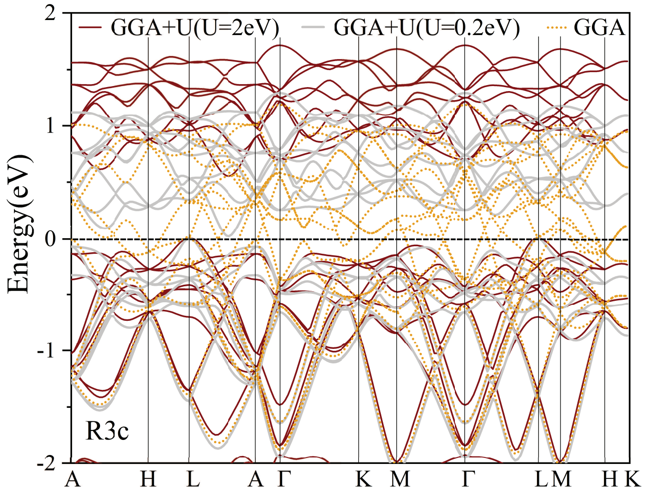

Figure SM1. Band structures of the polar NCS Rc3 hexagonal supercell of $LiOsO_3$ calculated by the non-spin-polarized PBE-GGA [35] (yellow color), and spin-polarized GGA+U [36, 37, 38, 39] considering G-type antiferromagnetic (G-AFM) ordering with U = 0.2 eV (gray color) and 2 eV (purple color).

defined for $m \in [\mathbb{J}^{(\mathrm{II})}+1, \mathbb{J}^{(\mathrm{II})}+\mathbb{J}^{(\mathrm{I})}]$ as follows:

$$\mathscr{U}_{m,\mathbf{k}}^{(\lambda),(\mathrm{I})}(\mathbf{r}) = \sum_{l=\mathbb{J}^{(\mathrm{II})}+1}^{\mathbb{J}-\mathbb{J}^{(\mathrm{II})}} c_{lm} u_{l,\mathbf{k}}^{(\lambda),(\mathrm{II})}(\mathbf{r}), \tag{37}$$

where the upper limit of the summation satisfies $\mathbb{J}-\mathbb{J}^{(\mathrm{II})} \geq \mathbb{J}^{(\mathrm{I})}$. The inequality is strict when the chosen isolated group of valence and conduction bands in part I plus II does not include all the number of bands generated by DFT calculation $\mathbb{J}$ and the equality holds when the total number of bands $\mathbb{J}$ are included in the mixed part including both the valence and low-lying conduction bands, i.e. mixed part means pure part I including the occupied valence bands only plus pure part II including the empty low-lying conduction bands only. The columns of the matrix c are orthonormal and represent the coordinates of the extra degrees of freedom $\left\{\mathscr{U}_{m,\mathbf{k}}^{(\lambda),(\mathrm{I})}(\mathbf{r})\right\}$ with respect to the $(\mathbb{J}-\mathbb{J}^{(\mathrm{II})})$-dimensional space of the unoccupied eigenstates. The matrix U is unitary and represents a rotation in the space of the functions $\left\{u_{1,\mathbf{k}}^{(\lambda),(\mathrm{II})}(\mathbf{r}),\ldots,u_{\mathbb{J}^{(\mathrm{II})},\mathbf{k}}^{(\lambda),(\mathrm{II})}(\mathbf{r});\mathscr{U}_{\mathbb{J}^{(\mathrm{II})}+1,\mathbf{k}}^{(\lambda),(\mathrm{I})}(\mathbf{r}),\ldots,\mathscr{U}_{\mathbb{J}^{(\mathrm{II})}+\mathbb{J}^{(\mathrm{I})},\mathbf{k}}^{(\lambda),(\mathrm{I})}(\mathbf{r})\right\}$.

Despite the generalizations made, however, the formulas Eqs. (16) to (18) or Eqs. (33) to (35) can be still only applied on insulators or semiconductors having non-zero bandgaps, see the occupation number 2 in these equations. We modify these formulas by considering correct occupation numbers for each Wannier centers in Sec. (3.2) so that they can be applicable for ferroelectric metals, as well. In this work, using the generalized maximally localized Wannier functions obtained principally from Eq. (24) and calculated practically within the self-consistently procedure discussed above through Eqs. (23) to (32), and modifying the second term of Eq. (35), i.e. Eq. (34), as discussed in Sec. 3.2, we calculate the total polarization vectors for structures "$\lambda = 0$" and "$\lambda = 1$". Our review in this supplementary is so presented that solely our modification discussed in Sec. 3.2 is made clear. For a comprehensive review of the maximally localized Wannier functions scheme and its applications in the transport properties of nanostructures, we refer the readers to Refs. [22] and [34], respectively.

2 OBSTRUCTS AND DEMAND

SEP is the key character of any normal FE materials [40, 41, 42, 43]. It can be calculated by the modern theory of polarization [1, 2, 3, 4, 5, 6, 7, 8, 9] employing two different approaches, i.e., Bp [44, 45, 46, 47] and maximally localized Wf [11, 12, 13, 14, 16, 48]. On the one hand, however, in FE-LMs, none of these standard methods can be applied to calculate their SEPs because, in these approaches, all the valence states entered in the electronic part of the polarization are considered to be completely full [49]. But, in metals, there are fractional states nearby the Fermi level. In fact, polarization expressions were routinely formulated in the Bp and Wf methods of polarization for the normal ferroelectric having nonzero bandgaps only, because polarization was not commonly expected to occur in metals before 1965 [50]. Therefore, the Bp [44, 45, 46, 47] and Wf [11, 12, 13, 14, 16, 48] approaches in their standard forms are useless for metals [49]. This is the main historical obstruct that manifests itself in practice as two practical obstructs which are discussed below.

Before continuing it would be noted that the necessary underlying idea behind the modern theory of polarization [1, 2, 3, 4, 5, 6, 7, 8, 9] required to follow the discussions presented in this work is compactly represented and reformulated in Sec. 1. In order to concise the current discussion, the formulation and definitions are presented in subsections 1.1 and 1.2 so that they can be readily accessible with no need to redefine every details required here.

In the Bp approach, the total microscopic EP is defined by Eq. (1). The total EP can be related to the total Berry phase via Eq. (2). The first term of Eq. (1), which is the ionic contribution to the EP, is also related to the ionic Berry phase by Eq. (3). Furthermore, the first term of Eq. (1), which contains the electronic contribution to the EP, is related to the electronic Berry phase principally through Eqs. (4) to (9) or practically by Eqs. (10) and (12) or more practically and conveniently by Eqs. (11) and (12), as well. However, the electronic part is well defined only for those materials that have energy gap. To be more specific, the second term of Eq. (1), or its transformed expression given by Eq. (9) containing the electronic Berry phase given by Eq. (4) or its practical form given by Eq. (11) or even its more practical and convenient form given by Eq. (11) and whence Eq. (12), cannot be applied on the metals due to the following two practical obstructs. The first practical obstruct is that all the bands are assumed to be completely filled in Eqs. (1), (2), and (4) to (12). In these electronic equations, the occupation number is set to 2 for every band. In a metal, however, some of the bands cross the Fermi level. The latter bands cannot be fully filled. These bands are partially occupied. Hence, the occupation numbers can no longer remain 2 for the bands crossing the Fermi level. Therefore, the above assumption made is not satisfied by the partially occupied bands and thence even the practical Eq. (11) loses its applicability for metals. The second practical obstruct is that in metals the occupation numbers of the partially occupied bands in the vicinity of the Fermi level depend on the k-points generated as a full mesh in the Brillouin zone (FBZ). However, the Berry phase can be gauge-dependent under unitary transformation, only if the bands contributing to the overlap matrix in the Eq. (11) do not depend on k which is not clearly satisfied by a metal [49]. A. Filippetti et al. [49] have overcome the above obstructs for a special system, i.e., the $Pm2_1n$ phase of the layered perovskite $Bi_5Ti_5O_{17}$ (Bi-5517). The Bi-5517 system has rather flat bands along Γ-Y direction of the Brillouin zone. The conduction band bottom density of states (DOS) of this special layered system has shown two-dimensional character, with 40% of the conduction charge confined within the central Ti layer of each block, 25% in each of the two intermediate layers and only 5% in each of the edge Ti's. Furthermore, they [49] have shown that the Bi-5517 layers behave as an insulator along the polarization direction. The bands of Bi-5517 along the polarization direction do not cross the Fermi level, and the Bi-5517 layers behave as a metal only at the surface perpendicular to the polarization direction [49]. However, these specific behaviors and conditions of Bi-5517, which are mandatory to use the method proposed in Ref. [49], may not be occurred for all the other FE-LMs. For instance, the band structure of the $LiOsO_3$ compound does not show these special behaviors and thence do not satisfy the conditions of the method proposed for the Bi-5517 layers. In order to show this, the band structures are calculated for the polar NCS Rc3 hexagonal supercell of $LiOsO_3$ in the framework of the DFT [51, 52], employing the full-potential APW+lo method [28, 29, 30] as implemented in the WIEN2k code [26, 27], using the Perdew-Becke-Erzenhof (PBE)-GGA functional [35], see Fig. SM1. It is already known that $LiOsO_3$ behaves as a nonmagnetic metal [53, 54, 55]. Furthermore, Liu et al. [56], by DFT [51, 52] calculations using the pseudopotential-based VASP code [57, 58, 59, 60, 61], have shown that the effect of spin-orbit coupling (SOC) in spite of its large strength is small and the electronic correlation is weak in $LiOsO_3$. Therefore, the PBE-GGA band structure, as shown in Fig. SM1 calculated in the absence of spin-polarization (SP) excluding SOC, can be reliable enough for the current purpose in this section. We have also opened the bandgap of $LiOsO_3$ by the spin-polarized GGA+U [36, 37, 38, 39] with U = 0.2 eV considering G-type antiferromagnetic (G-AFM) ordering as well as GGA+U with U = 2 eV for the G-AFM ordering, as would be seen in Fig. SM1. By this way, the $LiOsO_3$ compound can be hypothetically forced to behave as a semiconductor. In this case, the standard Berry phase theory can be applicable, as to be discussed later, see also Ref. [62]. Here, for the current purposes, however, let us consider the PBE-GGA band structure only to avoid deviating from current discussion. Our PBE-GGA band structure in agreement with the earlier theoretical and experimental results [53, 56, 62, 63, 64, 65, 66, 67, 68, 69, 70, 71, 72] show that $LiOsO_3$ is a metal. The PBE-GGA band structure shows that the bands cross the Fermi level along both the perpendicular (Γ-K, Γ-M, A-H, and A-L) and the parallel (Γ-A, M-L, and K-H) directions to the polarization direction, see Figs. SM1 and Fig. 2 of the main text. The bands also do not show the flat behavior along the strings parallel to or on the 2-denominational sheets perpendicular to the polarization, see Fig. SM1 and Fig. 2 of the main text. Therefore, $LiOsO_3$ does not behave like Bi-5517 layers. Hence, alternative practical approaches are required for the study of the SEPs of the other FE-LMs including the compound under consideration.

On the other hand, the field of FE-LMs is under development by the researchers' participation in this new field [49, 53, 54, 56, 63, 64, 65, 66, 68, 69, 70, 71, 72, 73, 74, 75, 76, 77, 78, 79, 80] after the discovery of ferroelectricity in $LiOsO_3$ metal [54] in the framework of the A&B-theory [50]. This causes the demand of developing and designing new ferroelectric-like materials to increase, which, in turn, doubles the need for generalization of the Bp and Wf schemes for the study of the FE-LMs. Thus, the formulation would be modified by considering and resolving the above two practical obstructs more generally so that it can be also applied in practice on any ferroelectric-like metals.

Therefore, due to the above obstructs and demand, we below modify the EP formulae expressed in the frameworks of

Bp and Wf approaches to make feasible the SEP calculations of FE-LMs. Our modifications made in the framework of the Wannier functions (Berry phase) approach are direct (mean-filed-like). In the anticipation of further investigation, a comprehensive and systematic way to predict the SEP of a FE-LM using the Berry phase approach can be to calculate $\varphi^{\lambda}_{el,n,\mu}$ directly by Eq. (4) and then apply our modifications by considering occupation numbers band by band, as proposed in Sec. 3.1. However, since this direct approach is cumbersome [2], in this work, we still prefer to utilize the more practical and convenient approach proposed by R. D. King-Smith and David Vanderbilt [2] and then modify it by a mean-filed-like approach, as proposed and performed in Sec. 3.1. Instead, in addition to the Berry phase approach, in this paper, we comprehensively and systematically calculate the SEP by directly evaluating, as proposed in Refs. [2, 81], the electronic polarization given in Eq. (5) using the Wannier functions approach [11, 12, 13, 14, 16, 48] and then modifying it accurately without applying the mean-filed-like method, see "SEP of $LiOsO_3$: mBp approach of electric polarization" of the main text. We show in "SEP of $LiOsO_3$: mBp approach of electric polarization" and "SEP of $LiOsO_3$: mWf approach of electric polarization" of the main text that the SEP results of the FE-LM in question calculated by our mean-field-like mBp and mWf approaches of polarization are in excellent agreement with each other. This validates the accuracy of the results and the modifications made and also show that the mean-filed-like Berry phase approach is as reliable as the Wannier functions approach.

3 EXTENDED EP METHODOLOGY FOR FE-LMs

To discuss the methodology, we would first classify the bands. Although the classification could be performed even by a schematic band structure showing only a metallic behavior, we have found that one of the genuine band structures of the system in question is quite convenient for this purpose, see Fig. 1 of the main text. We have obtained this metallic band structure by performing *ab initio* calculations, as discussed in Sec. 4, using the PBE-GGA functional [35] for the rhombohedral structure of $LiOsO_3$. The actual band structure of the main hexagonal supercell used in this work to calculate the EP is shown Fig. SM1. However, for the current purpose of classification, it is more convenient to use the neatly ordered band structure shown in Fig. 1 of the main text instead of the higgledy-piggledy band structure shown in Fig. SM1, see Sec. 5.3 for more elucidation.

Let us classify the bands of $LiOsO_3$ into three classes. These classes are labeled by I, I* and II, as shown in Fig. 1 of the main text. In the latter figure, we classify the bands into three classes. These classes are labeled by I, I* and II. These labels are dual-purpose and, in addition to their roles in classifying the bands, they can be also used to distinguish three different energy intervals indicated in Fig. 1 of the main paper. The class I includes valence bands which are crossing the Fermi level and conduction bands. The highest energy limit of class I* is the Fermi energy, while its lowest energy limit possesses in common with that of class I. Therefore, the class I* is a subclass of class I. Only some of the valence bands in classes I and I* are full while their other remaining valence bands are partially filled and hence they can only partially contribute into the electronic part of the Berry phase. The conduction bands in class I are empty and hence their contributions into the electronic part of the EP are zero. The class II includes valence bands which are all fully occupied. The valence bands included in class II are well separated by an energy interval of ≈ 0.4 eV from the bands included in class I, see Fig. 1 of the main article. The class I includes valence bands which are all either fully occupied or partially occupied and conduction bands which are unoccupied. The valence and conduction bands included in class I, as shown in Fig. 1 of the main manuscript, are also well separated by an energy gap from their upper conduction bands, not shown herein Fig. 1 of the main text. Therefore, class I (II) constitutes an isolated group of bands because it is well separated from class II (I) and its lower (higher) valence (conduction) bands, as shown in Fig. 1 of the main text. It is worth to highlight that a group of isolated bands and an isolated group of bands, which sometimes are taken to be identical, are different in this work. A group of bands is defined [13, 14, 15, 22] to be isolated if it is only well separated by finite gaps from all other higher and lower bands in the whole of the BZ. We define a group of isolated bands to be not only well separated by finite gaps from all other higher and lower bands in the whole of the BZ but also no band of the group can show degeneracies and cross any other bands as well as hybridize with itself. The composite bands, as defined in Ref. [13], are identical to an isolated group of bands, as defined in Ref. [13] and indicated in Refs.[14, 15, 22], while differ from a group of isolated bands, as defined above in this work. A group of isolated bands is also an isolated group of bands. However, an isolated group of bands may not be necessarily a group of isolated bands, because bands crossing and degeneracies as well as hybridizations within itself are arbitrary (forbidden) in the former (latter) group. Crystals having a group of isolated bands are ideal as can be hardly found in nature. This is why the localized Wannier functions method [2, 23] has been generalized to the maximally localized Wannier functions method [13, 14, 15, 22]. The zeroth step constitutes the backbone of the modified methods because the rest steps of the methods, as to be subsequently proposed in Secs. 3.1 and 3.2, are meaningful and/or applicable only for those metals whose bands can be classified as discussed above. For every metal such as normal metals, the above classification of the bands may not be possible. Therefore, our methods are inapplicable (applicable) for normal metals (only FE-LMs). We are now in a position to present our modified methods, i.e. mBp and mWf methods of polarization. Let us begin below with the mBp method of polarization including six steps.

3.1 Modified Berry phase (mBp) method of polarization

The contributions of the fully occupied valence bands of the class II into the electronic part of the electric polarization can be straightforwardly calculated by the standard version of the Berry phase approach, as expressed by Eqs. (11) and (12). The former equations have been already well formulated for fully occupied bands. This part of calculations converges rapidly over a limited set of k-points with no need for any further attempts beyond the standard procedure.

In the first step, we perform a regular self-consistent density functional theory (DFT) calculation using WIEN2k code [26, 27] for the structure λ. Then, we apply the standard Berry phase approach, as discussed in Sec. 1.1, over the DFT results using the BerryPI code [21]. In this way, we apply Eqs. (11) and (12) and calculate the Berry phase for the μ component of the polarization. Subsequently, we using Eq. (3) extract the ionic part of the polarization for the structure λ, i.e. $\varphi_{ion,\mu}^{(\lambda)}$. It is important to note that the electronic part of the Berry phase calculated in this first step is wrong for the FE-LM and should not be considered. In the first step, it is still assumed that all the valence bands of class I or I* are completely occupied which cannot be true for the FE-LM. Thus, only $\varphi_{ion,\mu}^{(\lambda)}$ calculated in the first step can be trusted and reserved.

In the second step, we restrict the energies to the energy interval II. We then using the self-consistent DFT results calculated in the first step recalculate the Berry phase applying again its standard approach. In this step, the bands of class I are omitted and thereby they are not allowed to contribute into the electronic part of the Berry phase. Therefore, in the second step, after applying Eq. (11), we obtain the electronic part of the Berry phase in μ direction for all the bands of class II at every $\mathbf{k}_\perp$ for the structure λ, i.e. $\varphi_{el,\mu}^{(\lambda),(\mathrm{II})}(\mathbf{k}_\perp)$. Then, by applying Eq. (12) on $\varphi_{el,\mu}^{(\lambda),(\mathrm{II})}(\mathbf{k}_\perp)$, we make the electronic Berry phase of class II independent of $\mathbf{k}_\perp$ by taking an average over the perpendicular area or over the discrete points $\mathbb{N}_{\mathbf{k}_\perp}$ of the 2D $\mathbf{k}_\perp$-point samples shown in Fig. 2 of the main text as $\varphi_{el,\mu}^{(\lambda),(\mathrm{II})}$. But the value of $\varphi_{el,\mu}^{(\lambda),(\mathrm{II})}$ is only a portion of the electronic part of the Berry phase. The $\varphi_{el,\mu}^{(\lambda),(\mathrm{II})}$ originates from bands of class II only. Therefore, $\varphi_{el,\mu}^{(\lambda),(\mathrm{II})}$ is also reserved as the reliable electronic part of the Berry phase of the structure λ at $\mathbf{k}_\perp$ in μ direction for the class II only.

Before starting the next step it is worth noting that the combination of Eqs. (11) and (12) is what is called the standard Berry phase approach for the prediction of the electric polarization, as presented in Ref. [2]. This approach in its standard form can be, in practice, used to calculate the electronic Berry phase numerically. In this approach [2], Eq. (12) plays a mean-field-like role. Thus, this approach [2], that is the combination of Eqs. (11) and (12), can be called a mean-field-like approach which is convenient for practical (numerical) usages. The electronic Berry phase in μ direction can be directly, in principle, evaluated for band n by Eq. (4), see Ref. [81] where J. Zak has shown that the energy bands in solids can be labeled by Berry's phase. In this case, it is not needed to take an average over the discrete points $\mathbb{N}_{\mathbf{k}_\perp}$ using the mean-field-like Eq. (12). However, direct evaluation of the polarization using Eq. (5) or Berry phase using Eq. (4), in practice, is cumbersome [2]. R. D. King-Smith and David Vanderbilt [2] have elegantly resolved this difficulty by introducing this appropriate mean-field-like approach instead of direct evaluation of the electronic Berry phase to speed up the numerical calculations within an acceptable accuracy. Let us, keeping the mean-field role of Eq. (12) in mind for the fourth step, turn our attention to the next (third) step.

In the third step, we would calculate the contributions of the bands of the class I into the electronic part of the Berry phase by temporarily assuming that all the bands of this class are completely occupied. The latter assumption is not obviously satisfied by all the bands of class I, as can be seen from Fig. 1 of the main text. However, the basic task of the next step is to modify the results calculated by this wrong temporarily assumption. To this end, in the third step, we restrict the energies to the energy interval I. We then using the self-consistent DFT results calculated in the first step recalculate the Berry phase applying once more its standard approach. In the third step, the bands of the class II are omitted and thereby they are not allowed to contribute into the electronic part of the Berry phase. Therefore, in the third step, we obtain the electronic part of the Berry phase for all the bands of the class I, $\varphi_{el,\mu}^{(\lambda),(\mathrm{I})}(\mathbf{k}_\perp)$, using Eq. (11). However, $\varphi_{el,\mu}^{(\lambda),(\mathrm{I})}(\mathbf{k}_\perp)$ does not give a valid value, because by applying Eq. (11) on the bands of class I it is assumed that all the bands of the class I are also fully occupied. This is in the case that there are fully and partially occupied valence as well as unoccupied conduction bands in the class I. We reserve $\varphi_{el,\mu}^{(\lambda),(\mathrm{I})}(\mathbf{k}_\perp)$ and modify it in the fourth step.

Before continuing it is noteworthy that the electronic part of the Berry phase calculated in the first step cannot be reproduced by adding the electronic part of the Berry phase calculated in the second step for the bands of the class II to the electronic part of the Berry phase calculated in the third step for the bands of the class I, *viz.* $\varphi_{el,\mu}^{(\lambda),(\mathrm{I})}(\mathbf{k}_\perp)+\varphi_{el,\mu}^{(\lambda),(\mathrm{II})}(\mathbf{k}_\perp)\neq\varphi_{el,\mu}^{(\lambda)}(\mathbf{k}_\perp)$. The source of the latter inequality originates from the natural fact that in the first step it is assumed that all the bands are fully occupied only up to the Fermi level, while in the third step all the bands of the class I including conduction bands are assumed to be fully occupied. These assumptions clearly are not the same as each other, however, it is obvious that both of them are incorrect. We have verified, not shown here, that the inequality becomes equality for semiconductors where all their valence bands are occupied below their corresponding Fermi levels and well separated by their bandgaps from their unoccupied conduction bands. This, consistent with Ref. [49], confirms that superposition of the partial contributions into the Berry phase can successfully yield the final total Berry phase. The success of the superposition of the partial contributions may not be

surprising because it can be also more clearly understood from the original Eq. (10). In this equation, we can split the sum into multiple sums over the limits of the summation. For instance, the number of bands can be split for the classes I and II as: $\sum_{n=1}^{\mathbb{M}} \xrightarrow{\text{splits to}} \sum_{n=1}^{\mathbb{M}_{\mathrm{II}}} + \sum_{n=\mathbb{M}_{\mathrm{II}}+1}^{\mathbb{M}}$. Here, $\mathbb{M}$ is the total number of occupied bands, $\mathbb{M}_{\mathrm{II}}$ is the number of occupied bands in class II, and $\mathbb{M} - \mathbb{M}_{\mathrm{II}} \equiv \mathscr{M}_{\mathrm{I}^*}$ is the remaining number of bands in class I*, which not all of them are necessarily fully occupied. The numbers of occupied bands $\mathbb{M}$ and $\mathbb{M}_{\mathrm{II}}$ as well as the number of bands $\mathscr{M}_{\mathrm{I}^*}$ are natural numbers. But the occupation numbers of all the $\mathscr{M}_{\mathrm{I}^*}$ bands are not natural numbers. The class I* contains both the fully and partially occupied bands so that $\mathscr{M}_{\mathrm{I}^*} = \mathbb{M}_{\mathrm{I}^*} + \widetilde{\mathbb{M}}_{\mathrm{I}^*}$, where $\mathbb{M}_{\mathrm{I}^*}$ ($\widetilde{\mathbb{M}}_{\mathrm{I}^*}$) is the number of fully (partially) occupied bands. Therefore, if we only consider $\mathbb{M}_{\mathrm{I}}^*$, we miss $\widetilde{\mathbb{M}}_{\mathrm{I}^*}$, and if we consider $\mathscr{M}_{\mathrm{I}^*}$, we cannot say that all of them are fully occupied. Consequently, the splitting of the summation over the number of occupied bands can be meaningful only if the class of the bands is so selected that all the bands are entirely included in the class, namely, no band inside the class can cross the upper and lower boundaries of the energy interval determined by the class of the bands. For instance, classes I and II are well defined because they have encompassed the whole of all their own bands entirely. However, class I* is not well defined because its upper limit which is the Fermi level is crossed by some of its bands. This implies that applying Eq. (10) or its practical form expressed by Eq. (11) on the bands of class I* is physically meaningless. This is why we have considered class II in the second step and class I (instead of class I*) in the third step. All the $\mathbb{M}_{\mathrm{I}}$ bands of the class I are temporarily assumed to be occupied in step 3, as well. For the metal under consideration, however, the class I* should be considered. Hence, in the next step, we intend to indirectly replace class I with class I* by modifying class I bit-by-bit cautiously through a mean-field-like approximation.

In the fourth step, the goal is to modify the undesired contributions of the bands of class I, as already calculated in the third step. The goal cannot be reached solely by restricting the energies, as performed successfully in the step two and unsuccessfully in the step three, to the energy interval I*. Although the undesired contributions of the conduction bands of the class I can be removed by selecting the region I*, the goal cannot be achieved because of the partially occupied valence bands existed in the region I*. Thus, we use another strategy here. Let us recall that the standard Berry phase approach [2] is itself a mean-field-like approach, see Eq. (12) where an average is taken over discrete $\mathbf{k}_\perp$. Here, the idea of the alternative strategy is to propose another mean-field-like approach but this time by taking average over the bands of class I which are all assumed to be occupied in step 3. By this way, we can practically extract, in a mean-field-like manner, the modified contribution of band n in class I at point $\mathbf{k}_\perp$ for structure λ to the electronic Berry phase in μ direction from $\varphi_{el,\mu}^{(\lambda),(\mathrm{I})}(\mathbf{k}_\perp)$ calculated in the third step. We have extracted this mean-field band-dependent contribution of the band n which is denoted as $\phi_{el,n,\mu}^{(\lambda),(\mathrm{I})}(\mathbf{k}_\perp)$ by multiplying the band-independent $\varphi_{el,\mu}^{(\lambda),(\mathrm{I})}(\mathbf{k}_\perp)$ with a normalized weight factor $\mathbb{w}_n^{(\lambda)}(\mathbf{k}_\perp)$ for band n at point $\mathbf{k}_\perp$ for structure λ as follows:

$$\phi_{el,n,\mu}^{(\lambda),(\mathrm{I})}(\mathbf{k}_\perp) = \mathbb{w}_n^{(\lambda)}(\mathbf{k}_\perp)\varphi_{el,\mu}^{(\lambda),(\mathrm{I})}(\mathbf{k}_\perp), \tag{38}$$

where

$$\mathbb{w}_n^{(\lambda)}(\mathbf{k}_\perp) = \mathbb{C}\frac{\mathbb{n}_n^{(\lambda)}(\mathbf{k}_\perp)}{\mathbb{n}_{max}^{(\lambda)}}, \tag{39}$$

where $\phi_{el,n,\mu}^{(\lambda),(\mathrm{I})}(\mathbf{k}_\perp)$ is the partial band-dependent contribution extracted by a mean-flied approximation from the band-independent $\varphi_{el,\mu}^{(\lambda),(\mathrm{I})}(\mathbf{k}_\perp)$ contribution calculated in the third step. Here, symbol φ stands for the Berry phase when it is determined from the standard Berry phase approach, while symbol ϕ is used when our modification is applied. $\mathbb{n}_n^{(\lambda)}(\mathbf{k}_\perp)$ is the occupancy of band n at point $\mathbf{k}_\perp$ for structure λ, $\mathbb{C}$ is a normalization factor, and $\mathbb{n}_{max}^{(\lambda)}$ is the maximum occupancy that a band at a point $\mathbf{k}$ in the FBZ of structure λ can hold. If $\mathbb{N}_{\mathbf{k}_{\mathrm{BZ}}}^{(\lambda)}$ is defined to be the total number of $\mathbf{k}$-points generated in the FBZ of structure λ, then $\mathbb{n}_{max}^{(\lambda)} = 2/\mathbb{N}_{\mathbf{k}_{\mathrm{BZ}}}^{(\lambda)}$. The occupation numbers $\mathbb{n}_n^{(\lambda)}(\mathbf{k}_\perp)$ and $\mathbb{n}_{max}^{(\lambda)}$ can be read from the self-consistent field DFT calculations performed in the first step. To derive the normalization constant $\mathbb{C}$, let us take summation over n on both sides of Eq. (38):

$$\sum_{n=1}^{\mathbb{M}_{\mathrm{I}}} \phi_{el,n,\mu}^{(\lambda),(\mathrm{I})}(\mathbf{k}_\perp) = \varphi_{el,\mu}^{(\lambda),(\mathrm{I})}(\mathbf{k}_\perp) \sum_{n=1}^{\mathbb{M}_{\mathrm{I}}} \mathbb{w}_n^{(\lambda)}(\mathbf{k}_\perp), \tag{40}$$

where $\varphi_{el,\mu}^{(\lambda),(\mathrm{I})}(\mathbf{k}_\perp)$ is pulled outside the summation sign as it does not depend on n. Since the normalization constant does not depend on the case, let us, without loss of generality, consider a case for which no correction is needed. In the absence of any corrections, it is expected that:

$$\sum_{n=1}^{\mathbb{M}_{\mathrm{I}}} \phi_{el,n,\mu}^{(\lambda),(\mathrm{I})}(\mathbf{k}_\perp) = \varphi_{el,\mu}^{(\lambda),(\mathrm{I})}(\mathbf{k}_\perp). \tag{41}$$

By combining Eqs. (40) and (41) and using Eq. (39) we have:

$$\sum_{n=1}^{\mathbb{M}_{\mathrm{I}}} \mathbb{w}_n^{(\lambda)}(\mathbf{k}_\perp) = \frac{\mathbb{C}}{\mathbb{n}_{max}^{(\lambda)}} \sum_{n=1}^{\mathbb{M}_{\mathrm{I}}} \mathbb{n}_n^{(\lambda)}(\mathbf{k}_\perp) = 1, \tag{42}$$

where $\mathbb{C}$ and $\mathbb{n}_{max}^{(\lambda)}$ are pulled outside the summation sign as they are both constant. In the absence of any corrections, $\mathbb{n}_n^{(\lambda)}(\mathbf{k}_\perp)$ is nothing more than $\mathbb{n}_{max}^{(\lambda)}$. Hence, in this case, the $\mathbb{n}_n^{(\lambda)}(\mathbf{k}_\perp)$ becomes also a constant and can be taken out of the sum. Therefore, from Eq. (42) the normalization factor can be derived to be $\mathbb{C} = 1/\mathbb{M}_{\mathrm{I}}$. By the latter relation, Eq. (39) can be represented as:

$$\mathbb{w}_n^{(\lambda)}(\mathbf{k}_\perp) = \frac{1}{\mathbb{M}_{\mathrm{I}}} \frac{\mathbb{n}_n^{(\lambda)}(\mathbf{k}_\perp)}{\mathbb{n}_{max}^{(\lambda)}}. \tag{43}$$

Let us before continuing discuss the properties of the weight factor, as expressed in Eq. (43), to show that how it can extract the energy interval I^* from the energy interval I. By this way, we also see that how the weight factor can replace the bands of class I having incorrect occupation numbers with the bands of class I^* considering their correct occupation numbers, by modifying the assumption made in step 3. The weight factor $\mathbb{w}_n^{(\lambda)}(\mathbf{k}_\perp)$ equals $1/\mathbb{M}_{\mathrm{I}}$ for every occupied valence band n inside class I, because the occupancy of an occupied valence band equals the maximum occupancy that this band can take at every point $\mathbf{k}_\perp$, *viz.* $\mathbb{n}_n^{(\lambda)}(\mathbf{k}_\perp) = 2/\mathbb{N}_{\mathbf{k}_{\mathrm{BZ}}}^{(\lambda)} = \mathbb{n}_{max}^{(\lambda)} \xrightarrow{\text{Eq. (43)}} \mathbb{w}_n^{(\lambda)}(\mathbf{k}_\perp) = 1/\mathbb{M}_{\mathrm{I}}$ for an occupied valence band n. This, in analogy to Eq.(42), again leads to $\sum_{n=1}^{\mathbb{M}_{\mathrm{I}}} \mathbb{w}_n^{(\lambda)}(\mathbf{k}_\perp) = \sum_{n=1}^{\mathbb{M}_{\mathrm{I}}} 1/\mathbb{M}_{\mathrm{I}} = 1/\mathbb{M}_{\mathrm{I}} \sum_{n=1}^{\mathbb{M}_{\mathrm{I}}} 1 = \mathbb{M}_{\mathrm{I}}/\mathbb{M}_{\mathrm{I}} = 1$. For an occupied band n, we have also $\sum_{n=1}^{\mathbb{M}_{\mathrm{I}}} \mathbb{w}_n^{(\lambda)}(\mathbf{k}_\perp) = \sum_{n=1}^{\mathbb{M}_{\mathrm{I}}} 1/\mathbb{M}_{\mathrm{I}} = 1/\mathbb{M}_{\mathrm{I}} \sum_{n=1}^{\mathbb{M}_{\mathrm{I}}} 1 = \mathbb{M}_{\mathrm{I}}/\mathbb{M}_{\mathrm{I}} = 1$. Therefore, from Eq. (38) we have $\phi_{el,n,\mu}^{(\lambda),(\mathrm{I})}(\mathbf{k}_\perp) = \varphi_{el,\mu}^{(\lambda),(\mathrm{I})}(\mathbf{k}_\perp)/\mathbb{M}_{\mathrm{I}}$ for an occupied valence band n. This ensures that $\varphi_{el,\mu}^{(\lambda),(\mathrm{I})}(\mathbf{k}_\perp)$ is diminished by the normalization factor after applying Eq. (38). We recall that the band-independent $\varphi_{el,\mu}^{(\lambda),(\mathrm{I})}(\mathbf{k}_\perp)$ is obtained in step 3 by temporarily assuming that all the bands of class I are fully occupied, while the band-dependent $\phi_{el,n,\mu}^{(\lambda),(\mathrm{I})}(\mathbf{k}_\perp)$ is introduced by Eq. (38) to modify this assumption band by band. Therefore, when $\varphi_{el,\mu}^{(\lambda),(\mathrm{I})}(\mathbf{k}_\perp)$ is pointed out, all the bands of class I is considered to be full, while when $\phi_{el,n,\mu}^{(\lambda),(\mathrm{I})}(\mathbf{k}_\perp)$ is pointed out, the band n of class I can be fully occupied, partially occupied, or empty. Thus, if the band n of class I in $\phi_{el,n,\mu}^{(\lambda),(\mathrm{I})}(\mathbf{k}_\perp)$ and all the bands of class I contributed in $\varphi_{el,\mu}^{(\lambda),(\mathrm{I})}(\mathbf{k}_\perp)$ are fully occupied, then the relation $\phi_{el,n,\mu}^{(\lambda),(\mathrm{I})}(\mathbf{k}_\perp) = \varphi_{el,\mu}^{(\lambda),(\mathrm{I})}(\mathbf{k}_\perp)/\mathbb{M}_{\mathrm{I}}$ perfectly shows that $\varphi_{el,\mu}^{(\lambda),(\mathrm{I})}(\mathbf{k}_\perp)$ contribution can be uniformly distributed in a mean-field manner over the partial band-dependent $\phi_{el,n,\mu}^{(\lambda),(\mathrm{I})}(\mathbf{k}_\perp)$ contributions. The relation $\phi_{el,n,\mu}^{(\lambda),(\mathrm{I})}(\mathbf{k}_\perp) = \varphi_{el,\mu}^{(\lambda),(\mathrm{I})}(\mathbf{k}_\perp)/\mathbb{M}_{\mathrm{I}}$ confirms that Eq. (38), as a mean-field approximation, can keep the occupied band n of class I. This is fine, because this occupied valence band of class I which also belongs to the class I^* is needed to reconstruct class I^* from class I. The weight factor $\mathbb{w}_n^{(\lambda)}(\mathbf{k}_\perp)$ vanishes for every empty conduction band n inside class I, because the occupancy of an empty conduction band equals zero, *viz.* $\mathbb{n}_n^{(\lambda)}(\mathbf{k}_\perp) = 0 \xrightarrow{\text{Eq. (43)}} \mathbb{w}_n^{(\lambda)}(\mathbf{k}_\perp) = 0$ for an empty conduction band n. Therefore, from Eq. (38) we have $\phi_{el,n,\mu}^{(\lambda),(\mathrm{I})}(\mathbf{k}_\perp) = 0$ for an empty conduction band n. This makes sure that $\varphi_{el,\mu}^{(\lambda),(\mathrm{I})}(\mathbf{k}_\perp)$ perfectly vanishes by applying the mean-field Eq. (38) on this empty band of class I. This is also favorable, because this empty conduction band which belongs to the class I is only temporarily assumed to be fully occupied in step three, and therefore, here in this step, time is apt to empty it by the correct occupation numbers calculated in the first step. This empty conduction band which does not belong to the class I^* is not needed to reconstruct class I^* from class I and should be removed. The weight factor $\mathbb{w}_n^{(\lambda)}(\mathbf{k}_\perp)$ is between zero and $1/\mathbb{M}_{\mathrm{I}}$ for a partially filled band n inside class I because the occupancy of a partially valence band is less than the maximum occupancy that a band at a point $\mathbf{k}$ can hold, *viz.* $0 < \mathbb{n}_n^{(\lambda)}(\mathbf{k}_\perp) < \mathbb{n}_{max}^{(\lambda)} \xrightarrow{\text{Eq. (43)}} 0 < \mathbb{w}_n^{(\lambda)}(\mathbf{k}_\perp) < 1/\mathbb{M}_{\mathrm{I}}$ for a partially band n. Therefore, from Eq. (38) we have $0 < \phi_{el,n,\mu}^{(\lambda),(\mathrm{I})}(\mathbf{k}_\perp) < \varphi_{el,\mu}^{(\lambda),(\mathrm{I})}(\mathbf{k}_\perp)/\mathbb{M}_{\mathrm{I}}$ for a partially occupied band n. This shows that $\varphi_{el,\mu}^{(\lambda),(\mathrm{I})}(\mathbf{k}_\perp)$, as originated from the fully occupied conduction band n of class I due to the wrong assumption made in step 3, is modified by applying Eqs. (38) and (39) on the bands of class I. This is desirable too, because this partially occupied band which belongs to the class I is only temporarily assumed to be fully occupied there in step three, and therefore, here, in step four, time is apt to tune its occupancy by the accurate occupation numbers obtained in step one. Although only some pieces of this partially occupied band belong to the class I^*, this band is also necessary to reconstruct class I^* from class I by tuning the occupancy of band n applying Eq. (38). This partially occupied band plays a crucial role to calculate EP for metal in practice. In essence, the weight factor, by keeping the desired occupied valence bands of class I, and removing the undesired empty conduction bands of class I, as well as modifying the undesired occupied conduction bands of class I to the desired partially occupied bands of class I^*, due to its properties discussed above can be used to transform the class I to the class I^*.

At a point $\mathbf{k}_\perp$, Eq. (38) yields the modified contribution for only a single band n of class I. However, all the bands of this class can contribute into the Berry phase at the point $\mathbf{k}_\perp$ taking the modifications discussed above into account. Therefore, we take summation of the modified partial band-dependent Berry phase $\phi_{el,n,\mu}^{(\lambda),(\mathrm{I})}(\mathbf{k}_\perp)$ over all the bands of class I:

$$\phi_{el,\mu}^{(\lambda),(\mathrm{I}^*)}(\mathbf{k}_\perp) = \sum_{n=1}^{\mathbb{M}_\mathrm{I}} \phi_{el,n,\mu}^{(\lambda),(\mathrm{I})}(\mathbf{k}_\perp), \tag{44}$$

where I* is used in the left hand side due to the properties of the weight factor. By substituting Eqs. (38) and (43) into Eq. (44), the latter equation can be represented as:

$$\begin{aligned}\phi_{el,\mu}^{(\lambda),(\mathrm{I}^*)}(\mathbf{k}_\perp) &= \frac{\sum_{n=1}^{\mathbb{M}_\mathrm{I}} \left[\mathbb{n}_n^{(\lambda)}(\mathbf{k}_\perp)\varphi_{el,\mu}^{(\lambda),(\mathrm{I})}(\mathbf{k}_\perp)\right]}{\mathbb{n}_{max}^{(\lambda)}\mathbb{M}_\mathrm{I}} \\ &= \frac{\mathbb{N}_{\mathbf{k}_{\mathrm{BZ}}}}{2\mathbb{M}_\mathrm{I}} \sum_{n=1}^{\mathbb{M}_\mathrm{I}} \left[\mathbb{n}_n^{(\lambda)}(\mathbf{k}_\perp)\varphi_{el,\mu}^{(\lambda),(\mathrm{I})}(\mathbf{k}_\perp)\right].\end{aligned} \tag{45}$$

In Eq. (45), $\phi_{el,\mu}^{(\lambda),(\mathrm{I}^*)}(\mathbf{k}_\perp)$, in the framework of the mean-field-like approximation expressed by Eqs. (38) and (43), gives the modified Berry phase of all the bands in μ direction for class I* only at a single point $\mathbf{k}_\perp$, as shown in Fig. 2 of the main text, for the structure λ. Now, we are in a position to apply the mean-field-like Eq. (12) of the standard Berry phase approach on the $\phi_{el,\mu}^{(\lambda),(\mathrm{I}^*)}(\mathbf{k}_\perp)$, as already known by Eq. (45). By applying Eq. (12), the electronic Berry phase of class I* can be made independent of $\mathbf{k}_\perp$ by taking an average over the perpendicular area $A_\perp$, as shown in Fig. 2 of the main text, or here numerically over the discrete points $\mathbb{N}_{\mathbf{k}_\perp}$ of the 2D $\mathbf{k}_\perp$-point samples shown in Fig. 2 of the main text as follows:

$$\begin{aligned}\phi_{el,\mu}^{(\lambda),(\mathrm{I}^*)} &= \frac{1}{A_\perp}\int_{A_\perp} dA_\perp \phi_{el,\mu}^{(\lambda),(\mathrm{I}^*)}(\mathbf{k}_\perp) \\ &\approx \frac{1}{\mathbb{N}_{k_\perp}} \sum_{\mathbf{k}_\perp} \phi_{el,\mu}^{(\lambda),(\mathrm{I}^*)}(\mathbf{k}_\perp),\end{aligned} \tag{46}$$

In the fifth step, we first find the total electronic Berry phase for structure λ in μ direction, i.e. $\phi_{el,\mu}^{(\lambda)}$, by combining the electronic Berry phase of class II for structure λ in μ direction calculated in step 2 as $\varphi_{el,\mu}^{(\lambda),(\mathrm{II})}$, with the electronic Berry phase of class I* for structure λ in μ direction calculated in step 4 as $\phi_{el,\mu}^{(\lambda),(\mathrm{I}^*)}$, *viz.* $\phi_{el,\mu}^{(\lambda)} = \varphi_{el,\mu}^{(\lambda),(\mathrm{II})} + \phi_{el,\mu}^{(\lambda),(\mathrm{I}^*)}$. Then, in order to find the total Berry phase for structure λ in μ direction, i.e. $\phi_\mu^{(\lambda)}$, we add $\phi_{el,\mu}^{(\lambda)}$ to the ionic Berry phase for structure λ in μ direction calculated and reserved in step 1 as $\varphi_{ion,\mu}^{(\lambda)}$, *viz.* $\phi_\mu^{(\lambda)} = 2\phi_{el,\mu}^{(\lambda)} + \varphi_{ion,\mu}^{(\lambda)}$, where the factor of 2 stands for the spin degeneracy in non-spin-polarized systems. For spin polarized systems Berry phase of spin up is added to that of spin down, see Eq. (2).

In the sixth step, we can substitute $\varphi_\mu^{(\lambda)}$ into Eq. (2). Then, after multiplying both sides of Eq. (2) by $\hat{\mathbf{e}}_\mu$ and taking summation over μ on both sides of the resultant equation, we obtain the polarization vector for structure λ as:

$$\sum_{\mu=1}^{3} P_\mu^{(\lambda)}\hat{\mathbf{e}}_\mu = \mathbf{P}^{(\lambda)} = \frac{e}{2\pi\Omega^{(\lambda)}} \sum_{\mu=1}^{3} \varphi_\mu^{(\lambda)} R_\mu^{(\lambda)}\hat{\mathbf{e}}_\mu. \tag{47}$$

But, in "SEP of $LiOsO_3$: mBp approach of electric polarization" of the main text, we prefer to first substitute $\varphi_{ion,\mu}^{(\lambda)}$ into Eq. (2) and obtain the ionic part of polarization. Then, we substitute the electronic parts of polarization $\varphi_{el,\mu}^{(\lambda),(\mathrm{II})}$, $\phi_{el,\mu}^{(\lambda),(\mathrm{I}^*)}$, and $\phi_{el,\mu}^{(\lambda)}$ one by one into Eq. (9) to obtain individually partial components of the polarization. By this way, instead of obtaining the total polarization only, we obtain the ionic part of polarization an all the partial contributions of the electronic polarization. By this way, we can by taking summation over all the partial polarizations obtain the total polarization, as well.

The procedure discussed above from step 1 to this stage of step 6 is performed twice a structure: one for structure "$\lambda = 0$" and the other for structure "$\lambda = 1$". By this way, we obtain the electric polarization vectors $\mathbf{P}^{(\lambda=0)}$ for the structure "$\lambda = 0$" and $\mathbf{P}^{(\lambda=0)}$ for the structure "$\lambda = 1$" individually. Finally, we can obtain the spontaneous polarization $\Delta\mathbf{P}$ as $\Delta\mathbf{P} = \mathbf{P}^{(\lambda=1)} - \mathbf{P}^{(\lambda=0)}$, employing the modern theory of polarization [1, 2, 3, 4, 5, 6, 7, 8, 9].

3.2 Modified Wannier functions (mWf) method of polarization

The electronic polarization in the framework of the Wannier functions for the structure λ can be only calculated for systems having nonzero bandgaps [2, 6]. The electronic polarization can be calculated within the Wannier functions approach either by Eqs. (16) and (17) which are applicable for insulators or semiconductors having groups of isolated bands [2] or by their corresponding generalized Eqs. (33) and (34) which are applicable for insulators or semiconductors having composite bands, i.e. isolated groups of bands as defined in Refs. [13, 14, 15, 22]. A group of isolated bands and an isolated group of bands, which sometimes are taken to be identical, are different in this work, as defined individually at the beginning of Sec. 1.2. The localized Wannier functions used in Eqs. (16) and (17) corresponding to a group of isolated valence bands [2] are generalized to the maximally localized Wannier functions used in Eqs. (33) and (34) obtaining from cell-periodic part of Bloch wavefunctions corresponding to the composite bands [13, 14]. The composite bands can include both valence and low-lying conduction bands [14]. Thygesen, Hansen, and Jacobsen developed an approach to construct partly occupied maximally localized Wannier functions [31, 32], see Eqs. (36) and (37). They have shown that the average localization per Wannier function depends on the number of included unoccupied orbitals so that the maximum localization can occur at an optimized number of included unoccupied orbitals [31], see also Fig. 4 of Ref. [32] and Fig. 9 of Ref. [22] and their related discussions. Andrinopoulos, Hine, and Arash Mostofi [82] improved the van der Waals contribution to the total energy of a system calculated with DFT using partly occupied MLWFs that include anti-bonding states instead of using maximally localized Wannier functions that include only the valence states. These extensions [31, 32, 82] allow us to produce maximally localized Wannier functions considering bands of class I including valence and low-lying conduction bands, see Fig. 1 of the main text. In metals, it is well known that [14, 31, 83], if occupied valence states are considered only, the generated Wannier functions may not be well localized. If in addition to the occupied valence states, however, the unoccupied conduction states are also considered, the localization of the Wannier functions produced can be efficiently improved [15, 22, 31, 82]. Therefore, adding unoccupied low-lying conduction states to the occupied valence states provides promising situation to study the metallic system under consideration in the framework of the maximally localized Wannier functions. Although the maximally localized Wannier functions have been well generalized to the partly occupied maximally localized Wannier functions[31, 32, 82], the applications of these generalization have not been used for the prediction of the electric polarization in FE-LMs. The occupancy used in the electronic polarization formulas Eqs. (16) to (18) as well as Eqs. (33) to (35) is still set to 2 for every Wannier center of charge even in the generalized Eqs. (33) to (35). This is correct only if all the Wannier centers are fully occupied. This is not the case, however, for metals having partially occupied Wannier centers. In metals, if we include low-lying conduction bands to the valence bands, the Wannier centers can be partially occupied and thence their occupation numbers can be less than 2. Furthermore, the occupation numbers of different partially occupied Wannier centers can be different from each other. These points together with the generalization made for constructing maximally localized Wannier functions [13, 14, 31, 32, 82] motivated us to extend Eq. (34) or equivalently Eq. (33) as:

$$\mathbf{P}_{el}^{(\lambda)} = \frac{e}{\Omega^{(\lambda)}} \sum_{n=1}^{\mathbb{J}} \mathbb{n}_{W_{n,\mathbf{R}}^{(\lambda)}} \langle \mathbf{r} \rangle_{W_{n,\mathbf{R}}^{(\lambda)}} \tag{48}$$

$$\equiv \frac{e}{\Omega^{(\lambda)}} \sum_{n=1}^{\mathbb{J}} \mathbb{n}_{W_{n,\mathbf{R}}^{(\lambda)}} \int \mathbf{r} \left| W_{n,\mathbf{R}}^{(\lambda)}(\mathbf{r}) \right|^2 d\mathbf{r} \tag{49}$$

$$= \frac{e}{\Omega^{(\lambda)}} \sum_{n=1}^{\mathbb{J}} \mathbb{n}_{W_{n,\mathbf{R}}^{(\lambda)}} \langle W_{n,\mathbf{R}}^{(\lambda)} | \mathbf{r} | W_{n,\mathbf{R}}^{(\lambda)} \rangle , \tag{50}$$

where $\mathbb{n}_{W_{n,\mathbf{R}}^{(\lambda)}}$ is the occupancy of Wannier center n and $\mathbb{J}$ is the number of Wannier centers in structure λ. Therefore, by plugging Eq. (48) into Eq. (35), the total polarization can be also generalized to:

$$\mathbf{P}^{(\lambda)} = \mathbf{P}_{ion}^{(\lambda)} + \mathbf{P}_{el}^{(\lambda)} = \frac{e}{\Omega^{(\lambda)}} \left[\sum_{s=1}^{\mathbb{N}} Z_s^{(\lambda)} \mathbf{r}_s^{(\lambda)} + \sum_{n=1}^{\mathbb{J}} \mathbb{n}_{W_{n,\mathbf{R}}^{(\lambda)}} \langle \mathbf{r} \rangle_{W_{n,\mathbf{R}}^{(\lambda)}} \right], \tag{51}$$

where the first (second) term can be physically (cautiously) interpreted as ionic (electronic) polarization per unit volume $\Omega^{(\lambda)}$ of structure λ originated from $\mathbb{N}$ ions ($\mathbb{J}$ Wannier centers) each with positive (negative) charges of $+eZ_s^{(\lambda)}$ ($-e\mathbb{n}_{W_{n,\mathbf{R}}^{(\lambda)}}$) positioned at $\mathbf{r}_s^{(\lambda)}$ ($\langle \mathbf{r} \rangle_{W_{n,\mathbf{R}}^{(\lambda)}}$). In the ionic polarization, $\mathbf{r}_s^{(\lambda)}$ is a classical position of ion s in structure λ, while in the electronic polarization, $\langle \mathbf{r} \rangle_{W_{n,\mathbf{R}}^{(\lambda)}}$ which represents the position of the Wannier center n in structure λ is an expectation value. Therefore, $\langle \mathbf{r} \rangle_{W_{n,\mathbf{R}}^{(\lambda)}}$ is not position of a classical particle and should be quantum mechanically interpreted and calculated using the integral appeared in the right-hand side of Eq. (49). In order to calculate the $\mathbf{P}^{(\lambda)}$, we first decompose the electronic polarization into

number of composite bands. For the case of $LiOsO_3$, there are two composite bands, i.e. isolated class of bands II including deep-lying fully occupied valence bands only and isolated class of bands I including shallow-lying fully and partially occupied valence bands as well as low-lying empty conduction bands. Thus, we decompose $\mathbf{P}_{el}^{(\lambda)}$ into two parts:

$$\begin{aligned}\mathbf{P}_{el}^{(\lambda)} &= \mathbf{P}_{el}^{(\lambda),(\mathrm{II})} + \mathbf{P}_{el}^{(\lambda),(\mathrm{I}^*)} \\ &= \frac{e}{\Omega^{(\lambda)}}\left[\sum_{n=1}^{\mathbb{J}^{(\mathrm{II})}} 2\,\langle \mathbf{r}\rangle_{W_{n,\mathbf{R}}^{(\lambda)}} + \sum_{n=1}^{\mathbb{J}^{(\mathrm{I})}} \mathbb{n}_{W_{n,\mathbf{R}}^{(\lambda)}}\,\langle \mathbf{r}\rangle_{W_{n,\mathbf{R}}^{(\lambda)}}\right],\end{aligned} \tag{52}$$

where $\mathbf{P}_{el}^{(\lambda),(\mathrm{II})}$ ($\mathbf{P}_{el}^{(\lambda),(\mathrm{I}^*)}$) is the partial electronic contribution of Wannier centers of class II (I^*) into the electronic part of polarization $\mathbf{P}_{el}^{(\lambda)}$ at lattice vector $\mathbf{R}$ in structure λ. Here, $\mathbb{J}^{(\mathrm{II})}$ ($\mathbb{J}^{(\mathrm{I})}$) in the first (second) sum is the number of Wannier centers of class II (I), see factor 2 ($\mathbb{n}_{W_{n,\mathbf{R}}}^{(\lambda)}$) inside the first (second) summation which accounts for fully (partially) occupation numbers of each Wannier centers individually so that $\mathbb{J} = \mathbb{J}^{(\mathrm{II})} + \mathbb{J}^{(\mathrm{I})}$. The $\mathbb{J}^{(\mathrm{I})}$, as the upper limit of the second sum in Eq. (52), refers to the Wannier center of class I. However, the occupation numbers $\mathbb{n}_{W_{n,\mathbf{R}}^{(\lambda)}}$ used in the second term of Eq. (52) are so determined subsequently in step three that the second term, $e/\Omega^{(\lambda)} \sum_{n=1}^{\mathbb{J}^{(\mathrm{I})}} \mathbb{n}_{W_{n,\mathbf{R}}^{(\lambda)}} \langle \mathbf{r}\rangle_{W_{n,\mathbf{R}}^{(\lambda)}}$ calculated subsequently in step four, itself refers to the polarization of class I^* and yields the desired $\mathbf{P}_{el}^{(\lambda),(\mathrm{I}^*)}$. Now, in order to complete the calculation of $\mathbf{P}^{(\lambda)}$ from Eqs. (51) and (52), we perform the following steps.

In the first step, we perform a regular self-consistent DFT calculation for the structure λ. Then, we restrict the energies to the energy interval II, as shown in Fig. 1 of the main text. Now, we apply self-consistently the standard maximally localized Wannier functions procedure, considering Eqs. (22) to (32), on the fully occupied valence composite bands of class II for structure λ, see Fig. 1 of the main text. This procedure is performed over the Bloch states calculated by WIEN2k package [26, 27] to obtain maximally localized Wannier functions and their centers of charges using Wannier90 code [11, 12, 13, 14, 15] and WIEN2WANNIER interface [16]. At this stage, we use Eq. (13) (or equivalently the first term of Eq. (51)) to calculate $\mathbf{P}_{ion}^{(\lambda)}$ entirely while we use the first term of Eq. (52) to calculate partial electronic polarization $\mathbf{P}_{el}^{(\lambda),(\mathrm{II})}$. The latter electronic polarization is partial because it needs to be completed by including contributions of the Wannier centers of class I, i.e. $\mathbf{P}_{el}^{(\lambda),(\mathrm{I})}$ as the second term of Eq. (52).

In the second step, we restrict the energies to the energy interval I, see Fig. 1 of the main text. Then, we apply self-consistently the generalized maximally localized Wannier functions procedure constructing partly occupied Wannier functions on the composite bands of class I including valence and conduction bands for structure λ, see Fig. 1 of the main text. By this way, we calculate the positions of the Wannier centers of the structure λ as $\int \mathbf{r}|W_{n,\mathbf{R}}^{(\lambda)}(\mathbf{r})|^2 d\mathbf{r} = \langle \mathbf{r}\rangle_{W_{n,\mathbf{R}}^{(\lambda)}} = (\langle x\rangle_{W_{n,\mathbf{R}}^{(\lambda)}}, \langle y\rangle_{W_{n,\mathbf{R}}^{(\lambda)}}, \langle z\rangle_{W_{n,\mathbf{R}}^{(\lambda)}})$ for Wannier centers $n = 1$ to $\mathbb{J}^{(\mathrm{I})}$. At this stage, we cannot calculate the remaining electronic polarization by Eq. (34), because in this equation the occupation numbers of all the Wannier centers are assumed to be 2, while the class I contains partially occupied Wannier centers. We cannot also use our generalized formulae Eqs. (11) to (14) or more specifically the second term of Eq. (52), because $\mathbb{n}_{W_{n,\mathbf{R}}^{(\lambda)}}$ still are unknown. Therefore, the main task of the next step is to determine the unknown occupation numbers $\mathbb{n}_{W_{n,\mathbf{R}}^{(\lambda)}}$ for the Wannier centers of class I. In the third step, we determine $\mathbb{n}_{W_{n,\mathbf{R}}^{(\lambda)}}$ so that the polarization calculated in the following fourth step gives the polarization $\mathbf{P}_{el}^{(\lambda),(\mathrm{I}^*)}$ which is related to the desired class I^*. To this end, we first individually project the DOS on each of the maximally localized Wannier centers for $n = 1$ to $\mathbb{J}^{(\mathrm{I})}$, see Fig. 3 (a) of the main text. For instance, the projected DOS, as shown in Fig. 3 (a) of the main text, is obtained by projecting the calculated DOS on one of the maximally localized Wannier centers which is shown in in Fig. 3 (b) of the main text. Then, we integrate each of the projected DOSs up to the Fermi level. By this, we obtain individually the areas under each of the DOSs projected on the maximally localized Wannier centers up to the Fermi level, e.g. see the filled area under the DOS shown in Fig. 3 (a) of the main text which gives the occupancy of the corresponding center of charge. The values of these areas are the desired occupation numbers $\mathbb{n}_{W_{n,\mathbf{R}}^{(\lambda)}}$.

In the fourth step, we multiply each of the Wannier centers $\langle \mathbf{r}\rangle_{W_{n,\mathbf{R}}^{(\lambda)}}$ obtained in the second step by their corresponding occupation numbers $\mathbb{n}_{W_{n,\mathbf{R}}^{(\lambda)}}$ obtained in the third step. By this way, $\mathbb{n}_{W_{n,\mathbf{R}}^{(\lambda)}} \langle \mathbf{r}\rangle_{W_{n,\mathbf{R}}^{(\lambda)}}$ are obtained. By substituting $\mathbb{n}_{W_{n,\mathbf{R}}^{(\lambda)}} \langle \mathbf{r}\rangle_{W_{n,\mathbf{R}}^{(\lambda)}}$ into the second term of Eq. (52), $\mathbf{P}_{el}^{(\lambda),(\mathrm{I}^*)}$ are calculated. Now, by adding $\mathbf{P}_{el}^{(\lambda),(\mathrm{I}^*)}$ calculated in this step to $\mathbf{P}_{el}^{(\lambda),(\mathrm{II})}$ calculated in the first step, we obtain the electronic polarization $\mathbf{P}_{el}^{(\lambda)}$ for structure λ using the generalized Eq. (52). Let us close this step by indicating a practical note. To this end, let us consider a system whose its polarization direction is pointed along only a single direc-

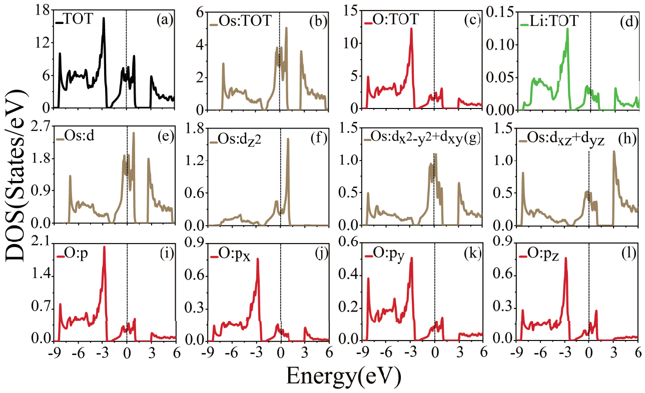

Figure SM2. Total and partial DOSs generated by the PBE-GGA DFT calculations for $LiOsO_3$ in its polar NCS rhombohedral structure.

tion. Such a system resemblances the case under study whose polarization direction is oriented along the c axis of the hexagonal supercell. In this case, it is enough to consider only $\mathbb{n}_{W_{n,\mathbf{R}}^{(\lambda)}} \langle z \rangle_{W_{n,\mathbf{R}}^{(\lambda)}}$ rather than $(\mathbb{n}_{W_{n,\mathbf{R}}^{(\lambda)}} \langle x \rangle_{W_{n,\mathbf{R}}^{(\lambda)}}, \mathbb{n}_{W_{n,\mathbf{R}}^{(\lambda)}} \langle y \rangle_{W_{n,\mathbf{R}}^{(\lambda)}}, \mathbb{n}_{W_{n,\mathbf{R}}^{(\lambda)}} \langle z \rangle_{W_{n,\mathbf{R}}^{(\lambda)}})$.

In the fifth step, based on Eq. (51), we add the ionic part of polarization $\mathbf{P}_{ion}^{(\lambda)}$ for structure λ, as obtained in the first step, to the electronic part of polarization $\mathbf{P}_{el}^{(\lambda)}$ for structure λ, as obtained in the fourth step. By this, we obtain the total electric polarization $\mathbf{P}^{(\lambda)}$ for structure λ. In analogous to the mBp approach of polarization discussed in Sec. 3.1, here, all the steps discussed above are also similarly performed for structures "$\lambda = 0$" and "$\lambda = 1$" individually. This leads to the electric polarization vectors $\mathbf{P}^{(\lambda=0)}$ for the structure "$\lambda = 0$" and $\mathbf{P}^{(\lambda=0)}$ for the structure "$\lambda = 1$". Hence, the spontaneous polarization $\Delta\mathbf{P}$ can be ultimately calculated as $\Delta\mathbf{P} = \mathbf{P}^{(\lambda=1)} - \mathbf{P}^{(\lambda=0)}$, using the modern theory of polarization [1, 2, 3, 4, 5, 6, 7, 8, 9].

It is worth mentioning that in the above procedure checking the accuracy of the maximally localized Wannier functions produced, as discussed in Sec. 1.2, plays a key role which can be verified as follows. To this end, for convenience, let us here consider the rhombohedral unit cell of the polar phase of the $LiOsO_3$ rather than its hexagonal supercell. The former (latter) unit (super) cell contains less (more) atoms produce 6 (18) bands in class I which results in (6) (18) Wannier centers. The less number of bands of the rhombohedral unit cell allows us to present the discussion more efficiently without loss of generality. The following validation procedure is similarly performed for the hexagonal supercells of the non-polar CS and polar NCS phases, not presented here. In Fig. 3 (c) of the main text the original band structure of the polar phase of the rhombohedral $LiOsO_3$ generated directly from a PBE-GGA DFT calculation, as shown by thin black bands, are compared with the Wannier-interpolated bands, as shown by thick blue bands, obtained from the subspace selected by an initially unconstrained projection onto atomic Os:d_{z^2}, and Os:$d_{x^2-y^2}$, as well as Os:d_{xy} orbitals. The comparison shows that the bands calculated by DFT are successfully regenerated by the Wannier-interpolated bands, see the coincidence of the DFT and Wannier bands in Fig. 3 (b) of the main text. This confirms that the initial guess, as discussed in Sec. 1.2, was suitable enough. The orbitals are selected by shedding light into the DOSs shown in Figs. SM2(a) to (l). In region I, which according to Fig. 1 of the main text is an energy interval around the Fermi level, contributions of Os:d_{z^2}, and Os:$d_{x^2-y^2}$+d_{xy} orbitals are larger than the other orbitals nearby the Fermi level. This can be verified by comparing Figs. SM2(f), (g), and (h), as the partial DOSs of Os atom, with the partial DOSs of Li and O atoms shown in Figs. SM2(d), (j), (k), and (l). Since Os:$d_{x^2-y^2}$ DOS is mixed to Os:d_{xy} DOS due to the high symmetry of the crystal, we first selected Os:d_{z^2}, and Os:$d_{x^2-y^2}$, as well as d_{xy} orbitals and produced the Wannier-interpolated bands. Then, keeping the Os:d_{z^2}, and Os:$d_{x^2-y^2}$ orbitals, we replaced d_{xy} with d_{xz} and reproduced the Wannier-interpolated bands, not shown here. By this replacement, we did not observe sensible changes in the Wannier-interpolated bands. We also replaced d_{xy} with d_{yz} and did not find considerable differences. From these observations, we concluded that d_{xy}, and d_{xz}, as well as d_{yz} almost play the same role and do not change the bands seriously. Furthermore, we also checked other partial orbitals of the Li atoms and found poor Wannier-interpolated bands which did not well coincide with the DFT bands. The poor bands originated from the tiny contribution of the total Li-DOS, see Fig. SM2(d). We also examined various combinations of the partial DOSs of oxygen atom with partial DOSs of Os atoms. The latter combinations could not provide acceptable results. Therefore, we can conclude that the Os:d_{z^2}, and Os:$d_{x^2-y^2}$, as well as Os:d_{xy} orbitals can provide a better initial guess and as a result better Wannier-interpolated bands, see Fig. 3 (b) of the main text and Fig. SM2.

In Fig. SM3, we have individually projected the band structure of the $LiOsO_3$ in its polar NCS rhombohedral phase on the 6 Wannier centers related to the energy interval I, shown in Fig. 1(a) of the main text. The amount of contributions of the bands in the 6 Wannier centers are given by the color bar located on the rightward side of Fig. SM3. The energy interval I

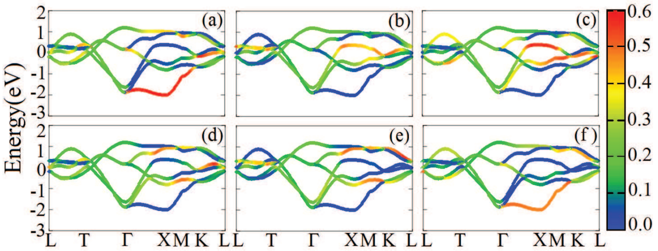

Figure SM3. Bands projected on the Wannier center number (a) one ($m = 1$), (b) two ($m = 2$), (c) three ($m = 3$), (d) four ($m = 4$), (e) five ($m = 5$), and (f) six ($m = 6$) associated to the energy interval I, see six bands shown in region I of Fig. 1 in the main text. The color bar indicates the amount of contributions of the bands in the Wannier centers. The initial DFT calculations were performed by the PBE-GGA DFT for the $LiOsO_3$ in its polar NCS rhombohedral structure. Fermi level is set to zero.

shows an isolated group of six bands including valence and low-lying conduction bands, see Fig. 1 (a) of the main text. The number of Wannier centers $\mathbb{J}$ is identical to the number of bands $\mathbb{M}$, e.g. $\mathbb{J}^{(\mathrm{I})} = \mathbb{M}^{(\mathrm{I})} = 6$ (18) in the energy interval I for the polar NCS rhombohedral (hexagonal) phase of the $LiOsO_3$, see Fig. 1 (a) of the main text and Figs. SM3(a) to (f). However, in contrast to the prediction made by Eq. (15), as the Wannier functions presented for a group of isolated bands, the results show that the bands are mixed, see Figs. SM3(a) to (f). The mixture of the bands is in agreement with the predication made by Eq. (22), as the Wannier functions presented for an isolated group of bands. This reconfirms that there may not be a one to one correspondence between a single localized Wannier function $W^{(\lambda)}_{n,\mathbf{R}}(\mathbf{r})$ labeled by subscript index n and a single band labeled by index m, see Figs. SM3(a) to (f) and the discussion presented for Eq. (22) and also compare it with that presented for Eq. (15). Taking the mixture of the bands and coexistence of the valence and low-lying conduction states in the energy interval I into account, we can state that the converged maximally localized Wannier functions are a combination of valence and conduction states. Hence, Wannier functions may not be decomposed into the valence and conduction states. This implies that the centers of a set of maximally localized Wannier functions constructed from valence states only may not be comparable with the centers of another set of maximally localized Wannier functions constructed from both valence and conduction states. The valence (low-lying conduction) states commonly correspond to the bonding-like (anti-bonding-like) Wannier functions whose centers can be centers of bonds (atomic positions). The centers of bonds which can be close to the Wannier centers originating from the valence states only differ from atomic positions which can close to the Wanner centers origination from valence and low-lying conduction states. These imply that the centers of maximally Wannier functions can depend on the states selected so that the maximally localized Wannier functions cannot be only attributed to the valence states if valence plus low-lying conduction states are used for constructing the maximally localized Wannier functions. Figs. SM3(a) and (f) show that contributions of the bands into the band structure projected on the Wannier centers 1 and 6 are comparable. The bands also almost equivalently contribute into the band structures projected on the Wannier centers 2 and 3, see Figs. SM3(b) and(c). The same contributions of the bands can be approximately observed into the band structure projected on the Wannier centers 4 and 5, see Figs. SM3(d) and(e). The source of these one to one correspondences between contributions of the bands into the Wannier centers 1 and 6, 2 and 3, as well as 4 and 5 originates from the symmetries of the two Os^{5+} ions in the rhombohedral unit cell of the $LiOsO_3$ crystal. The d-DOS of each Os^{5+} ion, as shown in Fig. SM2(e), splits due to the symmetry of LiOsO into 3 partial DOSs, i.e. Os:d_{z^2}, as shown in Fig. SM2(f), and Os:$d_{x^2-y^2}$+d_{xy}, Fig. SM2(g), as well as Os:d_{yz}, Fig. SM2(h). There are two (six) equivalent Os^{5+} ions in the rhombohedral (hexagonal) unit (super) cell of $LiOsO_3$. Therefore, there are only 3 nonequivalent Wannier centers among the $6 = 2 \times 3$ ($18 = 6 \times 3$) Wannier centers for the 2 (6) Os^{5+} ions of the rhombohedral (hexagonal) unit (super) cell of $LiOsO_3$. Therefore, the one to one correspondences stems from the equivalencies of the two Os^{5+} ions in the rhombohedral unit ell of $LiOsO_3$.

3.3 SEP direction in $LiOsO_3$ FE-LM

Here, let us show that the former R$\bar{3}$c structure consistent with its metallic behavior can be only taken into account as the non-polar phase which is defined and indicated by "$\lambda = 0$" as the initial structure of the adiabatic phase transition. To this end, we notice that in the CS R$\bar{3}$c structure, the Os^{5+} ions are positioned at the center of the OsO_6 octahedra. In an OsO_6 octahedron, an Os^{5+} ion is surrounded by six O^{2-} ions, as labeled by 1 to 6 in Fig. SM4, with an equal Os-O bond length in the R$\bar{3}$c structure shown in Fig. SM4(a). In order to quantitatively verify the above qualitative observation, the atomic positions and bond lengths between the relevant atoms are calculated for both the R$\bar{3}$c and R3c phases and the results are tabulated in Table SM1. The calculated bond lengths between Os_2 atom and every O_i atoms ($d_{\mathrm{Os}_2-\mathrm{O}_i}$) for $i = 1$ to 6, as tabulated in Table SM1, are all equal to 1.969 Å in the R$\bar{3}$c phase, *viz.* $d_{\mathrm{Os}_2-\mathrm{O}_i} = |\mathbf{r}_{\mathrm{Os}_2} - \mathbf{r}_{\mathrm{O}_i}| = \sqrt{(x_{\mathrm{Os}_2} - x_{\mathrm{O}_i})^2 + (y_{\mathrm{Os}_2} - y_{\mathrm{O}_i})^2 + (z_{\mathrm{Os}_2} - z_{\mathrm{O}_i})^2} = 1.969$ Å for

$i = 1$ to 6. The calculated bond length is in agreement with the value of 1.94 Å reported by Qiushi Yao *et al.* [65]. Furthermore, we have calculated the center of mass vector of the six oxygen atoms in Å, $\mathbf{R}^{CM}_{\{O_i\}}$(Å), located on the vertexes of the OsO_6 octahedron, as shown in Fig. SM4(a). The subscript symbol $\{O_i\}$ used in $\mathbf{R}^{CM}_{\{O_i\}}$ stands for the O_1 to O_6 of the OsO_6. The results, as tabulated in Table SM1 for the CS R$\bar{3}$c phase, show that $\mathbf{R}^{CM}_{\{O_i\}}$(Å)$= \sum_{i=1}^{6} m_{O_i}\mathbf{r}_{O_i}/\sum_{i=1}^{6} m_{O_i} = \sum_{i=1}^{6}\mathbf{r}_{O_i}/6 = (-2.568, 4.448, 6.631)$, where the oxygen masses are assumed to be equal with each other and hence canceled out from the numerator and denominator of the fraction $\mathbf{R}^{CM}_{\{O_i\}}$. The results show that the latter center of mass coincides with the position of Os_2 atom in the CS R$\bar{3}$c phase, *viz.* $\mathbf{r}_{Os_2}$(Å)$= (x_{Os_2}, y_{Os_2}, z_{Os_2}) = \mathbf{R}^{CM}_{\{O_i\}}$(Å) $= (-2.568, 4.448, 6.631)$, see Table SM1. This quantitatively confirms that the Os_2 atom is located at the center of the octahedron in the CS R$\bar{3}$c phase. Furthermore, the calculated bond lengths, as tabulated in Table SM1, also show that in the CS R$\bar{3}$c structure, the Li^+ ion is symmetrically positioned at the middle of the two adjacent Os^{5+} ions, *viz.* $d_{Li-Os_1} = d_{Li-Os_2} = 3.316$ Å, along the c-axis, *viz.* the x and y components of Li^+ ion are equal to the x and y components of the Os_1 and Os_2 atoms while their z components are different. Therefore, from the symmetric positions optimized for Os^{5+} and Li^+ ions, the CS R$\bar{3}$c can be considered as the non-polar phase needed for the SEP calculations. We return to this structure in "SEP of $LiOsO_3$: mBp approach of electric polarization" and "SEP of $LiOsO_3$: mWf approach of electric polarization" of the main text, where the SEPs are quantitatively calculated by two different methods using the CS R$\bar{3}$c structure as the initial non-polar structure labeled by "$\lambda = 0$".

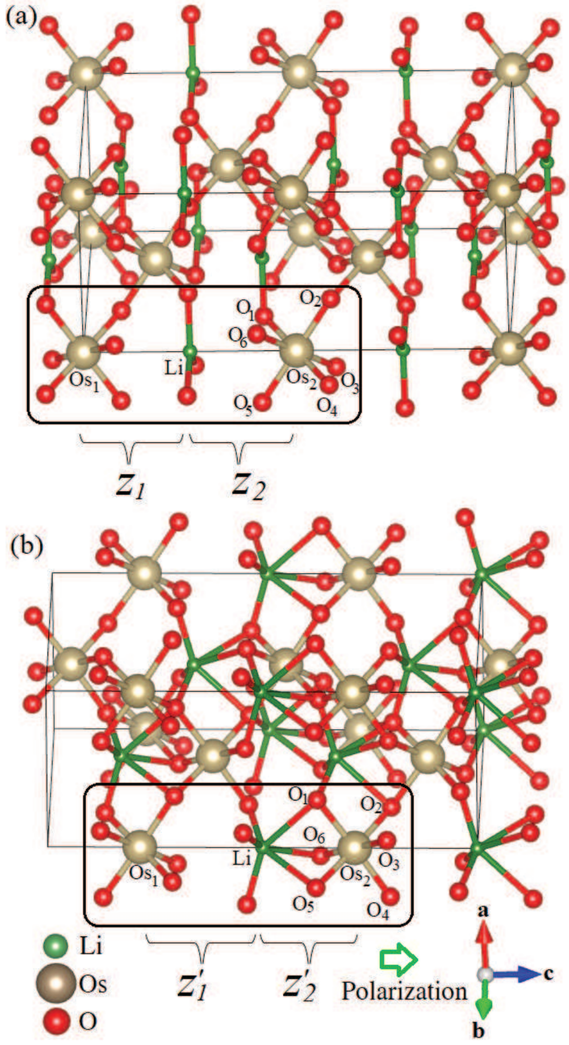

Figure SM4. The centosymmetric (R$\bar{3}$c) (a) and polar (R3c) (b) structures of $LiOsO_3$ including polar displacements and spontaneous polarization direction.

Now, let us turn our attention to the NCS R3c phase. We below show that this phase in spite of its metallic character can form the polar structure, as defined and indicated by "$\lambda = 1$" for the final structure of the adiabatic phase transition in

Table SM1. Atomic positions of the Li, Os_1, Os_2, and six O_i ($i = 1$ to 6) atoms for the non-polar CS R$\bar{3}$c structure, as shown in Fig. SM4(a), and polar NCS R3c structure, as shown in the Fig. SM4(b). The corresponding bond lengths and the centers of masses vectors are also given for the two (six) Os_1 and Os_2 (O_1, O_2, ..., O_6) atoms in both the phases. All the data are given in Å in this table.

Atomic Position(Å)	R$\bar{3}$c	R3c
(x_{Li}, y_{Li}, z_{Li})	$(-2.568, 4.448, 3.316)$	$(-2.538, 4.397, 6.704)$
$(x_{Os_1}, y_{Os_1}, z_{Os_1})$	$(-2.568, 4.448, 0.000)$	$(-2.538, 4.397, 2.873)$
$(x_{Os_2}, y_{Os_2}, z_{Os_2})$	$(-2.568, 4.448, 6.631)$	$(-2.538, 4.397, 9.579)$
$(x_{O_1}, y_{O_1}, z_{O_1})$	$(-1.622, 5.775, 5.526)$	$(-0.939, 4.527, 8.429)$
$(x_{O_2}, y_{O_2}, z_{O_2})$	$(-0.946, 4.292, 7.737)$	$(-1.852, 5.878, 10.664)$
$(x_{O_3}, y_{O_3}, z_{O_3})$	$(-3.245, 5.931, 7.737)$	$(-1.599, 3.061, 10.664)$
$(x_{O_4}, y_{O_4}, z_{O_4})$	$(-3.514, 3.121, 7.737)$	$(-4.164, 4.250, 10.664)$
$(x_{O_5}, y_{O_5}, z_{O_5})$	$(-4.190, 4.603, 5.526)$	$(-3.225, 2.947, 8.429)$
$(x_{O_6}, y_{O_6}, z_{O_6})$	$(-1.891, 2.965, 5.526)$	$(-3.450, 5.716, 8.429)$
**Bond Length(Å)**	**R$\bar{3}$c**	**R3c**
d_{Li-Os_1}	3.316	3.831
d_{Li-Os_2}	3.316	2.875
$d_{O_1-Os_2}$	1.969	1.974
$d_{O_2-Os_2}$	1.969	1.960
$d_{O_3-Os_2}$	1.969	1.960
$d_{O_4-Os_2}$	1.969	1.960
$d_{O_5-Os_2}$	1.969	1.974
$d_{O_6-Os_2}$	1.969	1.974
**Center of Mass(Å)**	**R$\bar{3}$c**	**R3c**
$\mathbf{R}^{CM}_{\{Os_i\}} = \frac{\sum_{i=1}^{2} \mathbf{r}_{Os_i}}{2}$	$(-2.568, 4.448, 3.316)$	$(-2.538, 4.397, 6.226)$
$\mathbf{R}^{CM}_{\{O_i\}} = \frac{\sum_{i=1}^{6} \mathbf{r}_{O_i}}{6}$	$(-2.568, 4.448, 6.631)$	$(-2.538, 4.397, 9.547)$

Sec. 1. To this end, in contrast to the CS R$\bar{3}$c structure, as shown in Fig. SM4(a), we notice that the Os_2 atom a little bit becomes off-centered from the center of the OsO_6 octahedron by 0.032 Å in the NCS R3c, as shown in Fig. SM4(b). The value of this eccentricity can be verified by the data presented in Table SM1 as: $\mathbf{r}_{Os_2}$(Å)$= (-2.538, 4.397, 9.579) \neq \mathbf{R}^{CM}_{\{O_i\}}$(Å) $= (-2.538, 4.397, 9.547)$ and hence $\left|\mathbf{r}_{Os_2} - \mathbf{R}^{CM}_{\{O_i\}}\right| = \sqrt{0^2 + 0^2 + (9.579 - 9.547)^2} = |9.579 - 9.547| = 0.032$ Å $\neq 0$. This value is in agreement with the value of 0.04 Å reported by Yao *et al.* [65]. Although this value is not too large, it is not also zero and hence causes the Os_2 atom to lose its symmetric position. Thus, the Os_2 atom becomes off-centered due to the movements of the six oxygen atoms towards outwards in the NCS R3c phase. The Os_2 atom becomes eccentric along the c-axis in the NCS R3c structure. This is along the c-axis because the R3c positions, as given in Table SM1, show that the x and y components of the Os_2 atom are equal to the x and y components of the center of mass of the six oxygen atoms of the OsO_6 and only their z components differ from each other, *viz.* $x_{Os_2} = R^{CM}_{x\{O_i\}}$ and $y_{Os_2} = R^{CM}_{y\{O_i\}}$ but $z_{Os_2} \neq R^{CM}_{z\{O_i\}}$. We have also noticed that the six Os-O bond lengths of the OsO_6 octahedron in the NCS R3c phase no longer remain either equal to those in the CS R$\bar{3}$c phase or equal to each other, see Table SM1. The three $d_{O_1-Os_2}$ and $d_{O_5-Os_2}$ as well as $d_{O_6-Os_2}$ bond lengths of the OsO_6 octahedra are expanded from 1.969 Å in the CS R$\bar{3}$c phase to 1.974 Å in the NCS R3c, while conversely the other remaining three $d_{O_2-Os_2}$ and $d_{O_3-Os_2}$ as well as $d_{O_4-Os_2}$ bond lengths of the OsO_6 octahedron are contracted from 1.969 Å in the CS R$\bar{3}$c phase to 1.960 Å in the NCS R3c phase. The results show that the expanded (contracted) bond lengths, i.e. $d_{O_1-Os_2}$ and $d_{O_5-Os_2}$ as well as $d_{O_6-Os_2}$ ($d_{O_2-Os_2}$ and $d_{O_3-Os_2}$ as well as $d_{O_4-Os_2}$), are assigned to those three oxygen atoms, i.e. O_1 and O_5 as well as O_6 (O_2 and O_3 as well as O_4), which are closer (further away) to (from) their corresponding nearest-neighbor Li^+ ions, see Fig. SM4(b) and Table SM1. The Li^+ ion also becomes eccentric from the centers of their adjacent Os^{5+} ions. These structural configurations are all found consistent with those reported in Ref. [84]. In the bond Os_1-Li-Os_2, the bond length

$d_{\mathrm{Li-Os_1}}$ is denoted by z_1 while the bond length $d_{\mathrm{Li-Os_2}}$ is denoted by z_2 in the CS R$\bar{3}$c structure, as shown in Fig. SM4(a). The bond length z_1 (z_2) is changed to z'_1 (z'_2), as denoted in Fig. SM4(b), by the phase transition from the CS to the NCS phase. The changes of the bond lengths originate from the eccentricities of the Os^{5+} and Li^{+} ions due to the phase transition from the R$\bar{3}$c to the R3c phase. We have then calculated the $z1 = z_2 = 3.316$ Å and thence found the z_1/z_2 ratio to be unity for the CS R$\bar{3}$c structure, *viz.* $z_1/z_2 = 3.316/3.316 = 1.000$, as expected for this no-polar phase, see Fig. SM4(a) and Table SM1. Qiushi Yao *et al.* [65], using PW91-GGA functional as implemented in the pseudopotential-based VASP code [57, 58, 59, 60, 61], have shown that the Li^{+} ions are displaced due to the forces exerted on them by the strong Os-O bonds originating from the eccentric Os^{5+} ions in the NCS R3c. For the NCS R3c structure, we have also calculated the $z'_1 = 3.831$ Å$> z'_2 = 2.875$ Å and as a consequence found the z'_1/z'_2 ratio to be 1.332, *viz.* $z'_1/z'_2 = 3.831/2.875 = 1.332$ which is deviated from the unity, see Fig. SM4(b) and compare it with Fig. SM4(a). This shows that the Li^{+} ion is displaced asymmetrically. Therefore, the Os_1—Li and Li-Os_2 bonds in Os_1—Li-Os_2 are made asymmetric due to the phase transition from R$\bar{3}$c to R3c. The distance between the two Os_1 and Os_2 atoms in the Os_1-Li-Os_2 bond is expanded from $z_1 + z_2 = 3.316 + 3.316 = 6.632$ Å in the CS phase to $z'_1 + z'_2 = 3.831 + 2.875 = 6.706$ Å in the NCS phase. The inversion symmetry is lost due to the deviation of z'_1/z'_2 ratio from unity originating from the eccentricities of the Li^{+} and Os^{5+} ions along the c-axis. The Li^{+} ion is moved away by $(z'_1 + z'_2)/2 - z'_2 = (z'_1 - z'_2)/2 = (3.831 - 2.875)/2 = 0.478$ Å from the center of the Os-Li-Os bond along the c-axis of the hexagonal structure. This value is in agreement with the value of 0.47 Å reported by Liu *et al.* [56] as well as the value of 0.4 Å reported by Yao *et al.* [65]. The value of 0.478 Å, as the off-centered displacement of the Li atom from the center of mass of the Os_1 and Os_12 atoms configured at the ends of the Os_1-Li-Os_2 configuration, is much larger than the small value of 0.032 Å, as the eccentricity of the Os_6 atom from the center of mass of the six oxygen atoms configured at the vertexes of the OsO_2 configuration. The Li displacement can be also more systematically obtained by the atomic positions given in Table SM1 as: $\mathbf{r}_{\mathrm{Li}}(\text{Å}) = (-2.538, 4.397, 6.704) \neq \mathbf{R}^{\mathrm{CM}}_{\{\mathrm{Os}_i\}}(\text{Å}) = (-2.538, 4.397, 6.226)$ and hence $\left|\mathbf{r}_{\mathrm{Li}} - \mathbf{R}^{\mathrm{CM}}_{\{\mathrm{Os}_i\}}\right| = \sqrt{0^2 + 0^2 + (6.704 - 6.226)^2} = |6.704 - 6.226| = 0.478 \text{ Å} \gg 0.032$ Å. By this way it can be also assured that the Li displacement is performed along the c-axis of the hexagonal NCS R3c structure, again since x and y but z components are equal, see Table SM1. From the eccentricities of the Li and Os_2 atoms discussed above, in agreement with [65], the NCS R3c phase can be considered as the polar phase required for the SEP calculations. Therefore, from the directions of the displacements determined above, the spontaneous polarization vector is predicted to orient along the c-axis. The orientation of the SEP predicted in this section is subsequently verified by considering both the non-polar CS R$\bar{3}$c and polar CS R3c phases in "SEP of $LiOsO_3$: mBp approach of electric polarization" and "SEP of $LiOsO_3$: mWf approach of electric polarization" of the main text using two different methods quantitatively, as well.

4 COMPUTATIONAL DETAILS

The first-principal calculations are performed in the framework of the DFT [51, 52], excluding and including (if necessary) spin- and/or orbital-polarization, as well as spin-orbit coupling, using the augmented planewaves plus local orbitals (APW+lo) method [28, 29, 30], employing LDA [85], PBE-GGA [35], LDA+U, and GGA+U [36, 37, 38, 39] functionals, as implemented in the WIEN2k package [26, 27, 86]. The Berry phase calculations are performed by the BerryPI code [21], as included in the WIEN2k package [26, 27, 86]. The Wannier calculations are performed by the Wannier90 code [11, 12, 13, 14, 15], as interfaced to the WIEN2k package [26, 27, 86] by the WIEN2WANNIER code [16]. The dynamical stability calculations were performed by the Phonopy package [87]. For the FE-LM $LiOsO_3$, a $9 \times 9 \times 2$ k-mesh is used for the energy window II, including fully occupied states only, while a much denser $24 \times 24 \times 8$ k-mesh is used for the energy window I, including valence and conduction states, see Fig. 1 of the main text where these energy windows are shown. For the other normal ferroelectrics a $9 \times 9 \times 2$ k-mesh and for the phonon calculation a $7 \times 7 \times 7$ k-mesh are used. The wavefunctions in the interstitial region were expanded in planewaves with a cutoff determined by $R_{\mathrm{MT}}K_{\mathrm{max}} = 7$. The periodic charge density and potential were Fourier expanded up to $G_{\mathrm{max}} = 12$ Bohr^{-1}. We adjusted the value of the mixing parameter of the charge density to 0.1, and used Broyden's scheme. The valence electrons are separated from the core electrons by setting separation energy to -9.0 Ry, resulting in 306 valence electrons distributed in $1s^2 2s^2$ orbitals of Li atoms, $5s^2 5p^6 6s^2 4f^{14} 5d^6$ orbitals of Os atoms, and $2s^2 2p^4$ orbitals of O atoms. The radii of the Muffin-Tin spheres were chosen to be 1.62, 2.03, and 1.66 Bohr for Li, Os, and O atoms, respectively. Internal parameters of the atomic positions were fully relaxed to make forces exerted on atoms less than 0.5 mRy/Bohr, and the lattice parameters were also simultaneously optimized. The forces were converged to less than 0.00001 eV/Å, in the phonon calculations performed, using the frozen-phonon method, by constructing a $2 \times 2 \times 2$ supercell containing 80 atoms, from the rhombohedral unit cell of the $LiOsO_3$.

$LiOsO_3$ is a ferromagnetic-like metal. Thus, the available methods of polarizations, i.e. Berry phase and Wannier functions, in their standard forms, are inapplicable for calculating spontaneous polarization of the $LiOsO_3$, see Sec. 3. Therefore, these methods of polarizations were modified in this work. Details of the modifications applied on the standard Berry phase (Wannier functions) method of polarization were discussed in Sec. 3.1 (Sec. 3.2).

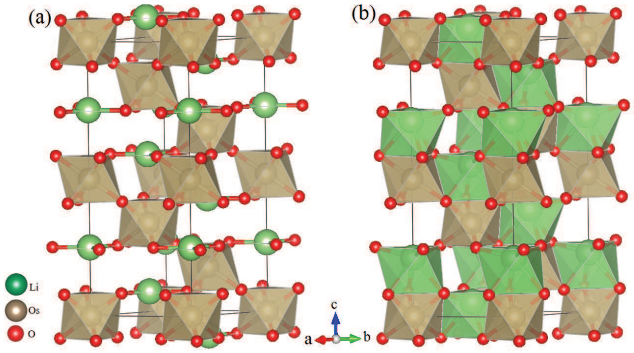

Figure SM5. The hexagonal (a) CS (non-polar) R$\bar{3}$c and (b) NCS (polar) R3c supercells of $LiOsO_3$. These hexagonal superstructures containing 30 atoms are obtained by transforming their corresponding rhombohedral structures containing 10 atoms.

5 CRYSTAL STRUCTURES

5.1 Rhombohedral Structures

$LiOsO_3$ is a non-magnetic (NM) metal which crystallizes in the rhombohedral system with the non-polar CS R$\bar{3}$c structure at 300 K and the polar NCS R3c structure at 10 K [53, 54, 55]. The non-polar CS R$\bar{3}$c (polar NCS R3c) structure constitutes the initial (final) structure "$\lambda = 0$" ("$\lambda = 1$") in the adiabatic phase transition. Let us below provide quantitatively essential information needed for constructing intermediate structures discussed in Sec. 5.4.

The space group number of the initial rhombohedral CS R$\bar{3}$c structure "$\lambda = 0$" is 167 with the lattice parameters of $a = b = 5.1360 \neq c = 13.2626$ Å and angles of $\alpha = \beta = 90 \neq \gamma = 120$ degrees. The conventional unit cell of the rhombohedral crystal system contains 10 atoms. This structure consists of 3 nonequivalent atoms, including Li with multiplicity 2, and Os with multiplicity 2, as well as O with multiplicity 6. This is in agreement with the information given in the supplementary materials of Ref. [66], see also pages 406 and 407 of Ref. [88]. The basis vectors (fractional atomic positions) are $(1/4, 1/4, 1/4)$ for one of the two Li atoms, and $(1/2, 1/2, 1/2)$ for one of the two Os atoms, as well as $(1/4, x, 1/2 - x) = (1/4, 0.8817, -0.3817) \equiv (1/4, 0.8817, -0.3817 + 1) = (1/4, 0.8817, 0.6183)$ for one of the six O atoms. In the latter position x is an internal parameter, see pages 406-407 Ref. [88]. Here, the single internal parameter x is optimized to be 0.6317. The negative value of -0.3817 is equivalently translated to the original cell by adding a unit lattice vector along the c-axis.

The space group number of the polar NCS R3c structure "$\lambda = 1$" is 161 with the lattice parameters $a = b = 5.0768 \neq c = 13.4118$ Å and angles $\alpha = \beta = 90 \neq \gamma = 120$ degrees. In analogous to the CS R$\bar{3}$c structure, the NCS R3c structure also contains the above 3 nonequivalent atoms with the same multiplicities. The basis vectors of the NCS R3c structure, which differ from those of the CS R$\bar{3}$c structure, are $(z1, z1, z1) = (0.4998, 0.4998, 0.4998)$ for one of the two Li atoms, and $(z2, z2, z2) = (0.7142, 0.7142, 0.7142)$ for one of the two Os atoms, as well as $(x3, y3, z3) = (0.4583, 0.8283, 0.0989)$ for one of the six O atoms. In this structure there are five internal parameters, see pages 359 and 360 of Ref. [88]. Here, the 5 internal parameters $z1$, $z2$, $x3$, $y3$, and $z3$ are optimized to be 0.4998, 0.7142, 0.4583, 0.8283, and 0.0989, respectively.

The basis vectors of the remaining 7 equivalent atoms can be found for both the NCS R3c (#161) and CS R$\bar{3}$c (#167) space groups individually by considering their corresponding symmetry and permutation operators, see pages 359-360 and 406-407 of Ref.[88]. Thus, we concentrate on the above 3 nonequivalent atoms only for each of the structures made in Sec. 5.4 where the intermediate structures are constructed for choosing the best branch of the adiabatic transition.

5.2 Hexagonal structures

For convenience, we have, by making a supercell, transformed the crystal system of the compound under consideration. The transformation is performed from the rhombohedral (trigonal) to the hexagonal crystal system shown in Fig. SM5. The hexagonal structure contains 30 atoms per its corresponding conventional cell: 6 equivalent Li atoms, 6 equivalent Os atoms, and 18 equivalent O atoms, see Figs. SM5(a) and (b). This is also consistent with the information given in the supplementary materials of Ref. [66]. The lattice parameters a in Å and the corresponding ratios c/a optimized by PBE-GGA for both the CS and NCS phases in their NM phase in agreement with the experimental data are presented in Table SM2. Table SM2 also includes more results which are discussed in Sec. 5.6.

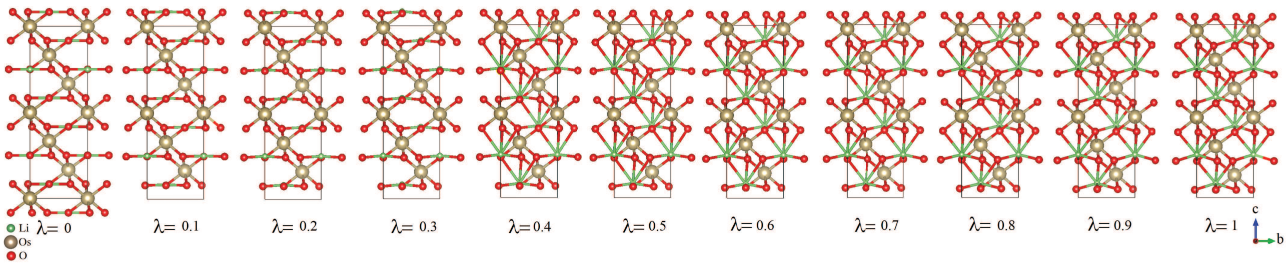

Figure SM6. Eleven hexagonal structures of $LiOsO_3$ including the non-polar CS R$\bar{3}$c structure indicated by "$\lambda = 0$", and 9 intermediate structures constructed by different distortions indicated by $\lambda = 0.1, 0.2, ..., 0.9$, as well as the polar NCS R3c structure indicated by "$\lambda = 1$" along the path from the non-polar CS R$\bar{3}$c ("$\lambda = 0$") to the polar NCS R3c ("$\lambda = 1$") phase. Here, Li, Os, and O atoms are shown by green, gray and red colors, respectively. Crystal coordinate system is also shown.

5.3 Rhombohedral versus hexagonal structures

Although the number of atoms and whence the cost of calculations are increased by our transformation, the hexagonal crystal system can be more convenient than the rhombohedral crystal system for the SEP calculations. This were so, because for the case of hexagonal structure, the direction of the polarization vector would be oriented only along one single direction, i.e. the c-axis of the hexagonal crystal system, as discussed in "SEP direction in $LiOsO_3$ FE-LM" of the main text. The latter suitable orientation was not the case for the rhombohedral lattice system, where the polarization vector had undesirable nonzero components along all the three axes of the rhombohedral structure. Thus, apart from Secs. 3 and 3.2, all the calculations and results in this work are obtained using the hexagonal crystal system in both its non-polar CS R$\bar{3}$c and polar NCS R3c space groups.

In Sec. 3, where only a schematic band structure of a metal is enough to present the fundamental aspects of the methodology, the band structure of the rhombohedral crystal in its polar NCS R3c space group is used. In contrast to the SEP calculations, for the methodology discussion, as presented in Sec. 3, the rhombohedral (trigonal) crystal system is more suitable than the hexagonal crystal system. The number of atoms of the rhombohedral unit cell is much less than the hexagonal supercell, as aforementioned above. Thus, the number of bands in the band structure of the rhombohedral unit cell are less than those of the corresponding hexagonal supercell. Hence, the less (large) number of bands can be less (more) mixed up and entangled to each other in the rhombohedral (hexagonal) structure. This can make the bands of the rhombohedral (hexagonal) structure more ordered and tidy (disordered and higgledy-piggledy). Although without loss of generality the methodology can be even discussed using a schematic band structure in Sec. 3, it is preferred to use the accurately calculated band structure of the ferroelectric-like compound under study instead of the schematic band structure. Therefore, for convenience in Sec. 3, the band structure of the rhombohedral crystal system in its polar NCS R3c space group, as shown in Fig. 1 of the main text, is considered as a metallic band sample for the $LiOsO_3$. This band structure shown in Fig. 1 of the main text is calculated using the PBE-GGA functional [35]. In fact, this band structure are not very higgledy-piggledy, as can be seen in Fig. 1 of the main text, and thereby it can well play the role of a schematic band structure of a typical metal in Sec. 3.

In Sec. 3.2, where the mWf method of polarization is discussed, for convenience we have used Figs. 3 of the main text, SM2, and SM3 which are also calculated by considering the rhombohedral structure to present the discussion more compactly. The number of DOSs and band structures of the hexagonal structure are quantitatively 3 times more than that of the rhombohedral structure without providing more qualitative information concerning the discussion presented in Sec. 3.2.

5.4 Nine intermediate structures

The adiabatic transition of $LiOsO_3$ starts from the non-polar CS R$\bar{3}$c structure, "$\lambda = 0$", and terminates to the polar NCS R3c structure, "$\lambda = 1$". Endpoints of the path indicated by "$\lambda = 0$" and "$\lambda = 1$" are not always sufficient to ensure that the branch is chosen correctly. To this end, in addition to the initial structure "$\lambda = 0$" and final structure "$\lambda = 1$", we have considered 9 intermediate structures. These intermediate structures are indicated by $\lambda = 0.1, 0.2, ..., 0.9$. The initial, and 9 intermediate, as well as final structures are shown in Fig. SM6 from left to right, respectively. These structures are constructed as follows.

We intend by distortions reach to the "$\lambda = 1$" structure from the "$\lambda = 0$" structure. However, in the initial structure "$\lambda = 0$", Li and O atoms are fixed by symmetry and only oxygen atoms can conditionally move due to the existence of the single internal parameter x in only two components of the positions of the O atoms, see Sec. 5.1. In the positions of the Li and Os atoms in the structure "$\lambda = 0$", there are not any internal parameters. Therefore, the Li and Os atoms are fixed by the symmetry of the non-polar CS R$\bar{3}$c structure and thereby they cannot move from their symmetrical positions. Fortunately, we notice that the structure "$\lambda = 1$" has 5 internal parameters so that all the 10 atoms in this structure can be displaced from its relaxed positions, see pages 359 and 360 of Ref. [88]. Therefore, instead of the non-polar CS struc-

ture, we apply the distortions on the polar NCS structure and gradually go from the structure "$\lambda = 0.1$" to the structure "$\lambda = 0.9$" step by step using the vector steps $\boldsymbol{\lambda}_{0.1}$ defined subsequently taking the following freedoms dictated by the symmetry of the structure into account. There are three different internal parameters $x1$, $y1$, and $z1$ in the position of the O atom, $(x1, y1, z1)$, in the polar NCS R3c structure, see Sec. 5.1. Similarly, these 3 different internal parameters symmetrically can be seen in all the components of the positions of the remaining 5 equivalent oxygen atoms, see pages 359-360 of Ref. [88]. Thus, the O atoms can be freely moved from its relaxed positions in all the 3 spatial directions arbitrarily in the polar NCS R3c structure without breaking the symmetry of the structure. We also notice that, however, there is only one internal parameter $z1$ for the Li atom as $(z1, z1, z1)$ in the polar NCS R3c structure, see Sec. 5.1. Similarly, this single internal parameter can be symmetrically seen in all the components of the position of the remaining equivalent Li atom as $(1/2 + z1, 1/2 + z1, 1/2 + z1)$, see pages 359-360 of Ref. [88]. Hence, both of the Li atoms can be moved only along $(1, 1, 1)$ direction, in the polar NCS R3c structure without breaking the symmetry of the structure. This implies that the Li atoms are not allowed by the symmetry of the polar NCS R3c structure to be arbitrarily moved along any other directions than $(1, 1, 1)$ direction. In analogous to the Li atoms, the Os atoms are also free to move along the $(1, 1, 1)$ direction only, in the polar NCS R3c structure due to the single internal parameter $z2$ indicated in Sec. 5.1, see also pages 359-360 of Ref. [88]. Now, before applying the distortions, we first note that the fractional Li position in the non-polar CS structure, $(1/4, 1/4, 1/4)$, is far from that in the polar NCS structure, $(0.4998, 0.4998, 0.4998)$, *viz.* $|(0.4998, 0.4998, 0.4998) - (1/4, 1/4, 1/4)| = |(0.2498, 0.2498, 0.2498)| = 0.2498\sqrt{3} \approx 0.4327$, see Sec. 5.1. Similarly, Os and O atoms in the non-polar CS structure are far from their corresponding Os and O atoms in the polar NCS structure, see the positions of Os and O atoms in Sec. 5.1. We have noticed that these large distances can cause practical problems to occur in the unwrapping procedure. In practice, it may be more suitable to take a shorter route via "$\lambda = 0$" to "$\lambda = 1$" during unwrapping procedure of the polarization in an adiabatic transition. Due to the freedom of the structure "$\lambda = 1$", we shift, without loss of generality, all the atoms by a constant vector of $(1/4, 1/4, 1/4) = 1/4(1, 1, 1)$ so that the new basis vectors of the polar NCS R3c structure becomes $(z1 - 1/4, z1 - 1/4, z1 - 1/4) = (0.4998 - 1/4, 0.4998 - 1/4, 0.4998 - 1/4) = (0.2498, 0.2498, 0.2498)$ for Li, and $(z2 - 1/4, z2 - 1/4, z2 - 1/4) = (0.7142 - 1/4, 0.7142 - 1/4, 0.7142 - 1/4) = (0.4642, 0.4642, 0.4642)$ for Os, as well as $(x3 - 1/4, y3 - 1/4, z3 - 1/4) = (0.4583 - 1/4, 0.8283 - 1/4, 0.0989 - 1/4) = (0.2083, 0.5783, -0.1511) \equiv (0.2083, 0.5783, -0.1511 + 1) = (0.2083, 0.5783, 0.8489)$ for O. We have checked by calculating the polarization using the new structure including shifted atoms and found exactly the same results compared to the results calculated by the original structure. This confirms that the results remain unchanged under the imposed shifts along the $(1, 1, 1)$ direction and consequently validates the shifts of the atoms. The new shifted position of the Li atom in the polar NCS structure, $(0.2498, 0.2498, 0.2498)$, is now made much closer to the position of the corresponding Li atom in the non-polar CS structure, $(1/4, 1/4, 1/4)$, *viz.* $(1/4, 1/4, 1/4) - (0.2498, 0.2498, 0.2498) = (0.0002, 0.0002, 0.0002)$. Similarly, for the Os atom we also see that the new position in the polar NCS structure after shifting $(0.4642, 0.4642, 0.4642)$ becomes closer to the corresponding position of the Os atom in the non-polar CS structure $(1/2, 1/2, 1/2)$, *viz.* $(1/2, 1/2, 1/2) - (0.4642, 0.4642, 0.4642) = (0.0358, 0.0358, 0.0358)$. However, the new shifted position of the O atom in the polar NCS structure, $(0.2083, 0.5783, 0.8489)$, does not still become sufficiently close to the corresponding O atom located at $(1/4, 0.8817, 0.6183)$, *viz.* $(1/4, 0.8817, 0.6183) - (0.2083, 0.5783, 0.8489) = (0.0417, 0.3034, -0.2306)$. By comparing $(1/4, 0.8817, 0.6183)$ with $(0.2083, 0.5783, 0.8489)$, we notice that if the y and z components in $(0.2083, 0.5783, 0.8489)$ are exchanged a newer position of $(0.2083, 0.8489, 0.5783)$ is obtained which is made close to the position of $(1/4, 0.8817, 0.6183)$, *viz.* $(1/4, 0.8817, 0.6183) - (0.2083, 0.8489, 0.5783) = (0.0417, 0.0328, 0.040)$. Fortunately, the exchange of the y and z components of the oxygen atom is permitted by the symmetry of the polar NCS R3c space group. This exchange does not violate the symmetry rules, because the position of the oxygen atom is $(x3, y3, z3)$ which contains 3 different internal parameters, as aforementioned above. Therefore, the above exchange of the components can be interpreted as a permitted translation in the real spatial space. To this end, the exchanged position $(0.2083, 0.8489, 0.5783)$ can be equivalently reproduced by moving the O atom from its original position of $(0.2083, 0.5783, 0.8489)$ using a translation vector of $(0, 0.2706, -0.2706)$ as $(0.2083, 0.5783, 0.8489) + (0, 0.2706, -0.2706) = (0.2083, 0.8489, 0.5783)$. Hence, $(0.2083, 0.5783, 0.8489)$ can be exchanged to $(0.2083, 0.8489, 0.5783)$ by $(0, 0.2706, -0.2706)$, *viz.* $(0.2083, 0.5783, 0.8489) \xrightarrow{(0,0.2706,-0.2706)} (0.2083, 0.8489, 0.5783)$. This exchange can be interpreted as a translation along $(0, 1, -1)$ direction with the magnitude of $0.2706\sqrt{0^2 + 1^2 + (-1)^2} = 0.2706\sqrt{2}$ which is permitted by the polar NCS R3c structure for the O atom due to the freedom included in its position $(x3, y3, z3)$ originating from the three different internal parameters discussed above. In summary, the positions of Li, and Os, as well as O can be represented as $(1/4, 1/4, 1/4)$, and $(1/2, 1/2, 1/2)$, as well as $(1/4, 0.8817, 0.6183)$ in "$\lambda = 0$" and $(0.2498, 0.2498, 0.2498)$, and $(0.4642, 0.4642, 0.4642)$, as well as $(0.2083, 0.8489, 0.5783)$ in "$\lambda = 1$", respectively.

Now, we are in a position to construct the structure "$\lambda = 0.1$". To this end, we define a step as $\lambda_{0.1}^{\mathrm{Li}_x} = (Li_x^{(\lambda=1)} - Li_x^{(\lambda=0)})/10 = (0.2498 - 1/4)/10 = -0.0002/10 = -0.00002$ for the variation of the position of Li atom along the x direction. The x, y, and z components of the Li atom are equal to each other in both the structures "$\lambda = 0$" and "$\lambda = 1$".

Then, we can see that $\lambda_{0.1}^{\mathrm{Li}_x} = \lambda_{0.1}^{\mathrm{Li}_y} = \lambda_{0.1}^{\mathrm{Li}_z} = -0.00002$. Thus, the position of the Li atom in the structure "$\lambda = 0.1$" can be obtained as $(1/4, 1/4, 1/4) + \boldsymbol{\lambda}_{0.1}^{\mathrm{Li}} = (1/4, 1/4, 1/4) + (\lambda_{0.1}^{\mathrm{Li}_x}, \lambda_{0.1}^{\mathrm{Li}_y}, \lambda_{0.1}^{\mathrm{Li}_z}) = (1/4 - 0.00002, 1/4 - 0.00002, 1/4 - 0.00002) = (0.2499, 0.2499, 0.2499)$. The position of the other Li atom can be obtained by symmetry of the structure. Similarly, we define a different step as $\lambda_{0.1}^{\mathrm{Os}_x} = (Os_x^{(\lambda=1)} - Os_x^{(\lambda=0)})/10 = (0.4642 - 1/2)/10 = -0.0358/10 = -0.00358$ for the variation of the position of Os atom along the x direction. The x, y, and z components of the Os atom are also equal to each other in both the structures "$\lambda = 0$" and "$\lambda = 1$". Then, we can see that $\lambda_{0.1}^{\mathrm{Os}_x} = \lambda_{0.1}^{\mathrm{Os}_y} = \lambda_{0.1}^{\mathrm{Os}_z} = -0.00358$. Thus, the position of the Os atom in the structure "$\lambda = 0.1$" can be obtained as $(1/2, 1/2, 1/2) + \boldsymbol{\lambda}_{0.1}^{\mathrm{Os}} = (1/2, 1/2, 1/2) + (\lambda_{0.1}^{\mathrm{Os}_x}, \lambda_{0.1}^{\mathrm{Os}_y}, \lambda_{0.1}^{\mathrm{Os}_z}) = (1/2 - 0.00358, 1/2 - 0.00358, 1/2 - 0.00358) = (0.4964, 0.4964, 0.4964)$. The position of the other Os atom can be also obtained by the symmetry of the structure. Similarly, we define another different step as $\lambda_{0.1}^{\mathrm{O}_x} = (O_x^{(\lambda=1)} - O_x^{(\lambda=0)})/10 = (0.2083 - 1/4)/10 = -0.0417/10 = -0.00417$ for the variation of the position of O atom along the x direction. In contrast to the Li and Os atoms, the x, y, and z components of the O atom differ from each other in both the structures "$\lambda = 0$" and "$\lambda = 1$". Therefore, we should define the steps along y and z directions individually for the O atom. For the O atom, the steps are defined to be $\lambda_{0.1}^{\mathrm{O}_y} = (O_y^{(\lambda=1)} - O_y^{(\lambda=0)})/10 = (0.8489 - 0.8817)/10 = -0.0328/10 = -0.00328$ along the y direction and $\lambda_{0.1}^{\mathrm{O}_z} = (O_z^{(\lambda=1)} - O_z^{(\lambda=0)})/10 = (0.5783 - 0.6183)/10 = -0.04/10 = -0.004$ along the z direction. Thus, the position of the O atom in the structure "$\lambda = 0.1$" can be obtained as $(1/4, 0.8817, 0.6183) + \boldsymbol{\lambda}_{0.1}^{\mathrm{O}} = (1/4, 0.8817, 0.6183) + (\lambda_{0.1}^{\mathrm{O}_x}, \lambda_{0.1}^{\mathrm{O}_y}, \lambda_{0.1}^{\mathrm{O}_z}) = (1/4 - 0.00417, 0.8817 - 0.00328, 0.6183 - 0.004) = (0.2458, 0.8784, 0.6143)$. The positions of the other 5 O atoms can be obtained by the symmetry of the structure, as well. Now, we consider the destination polar NCS R3c "$\lambda = 1$" as a sample structure file and construct the structure "$\lambda = 0.1$" by replacing the atomic positions of the destination polar NCS R3c structure with the new positions obtained above for the structure "$\lambda = 0.1$" one by one, *viz* we remove the atomic positions of the sample polar NCS R3c structure and then insert $(0.2499, 0.2499, 0.2499)$ for the Li atom, and $(0.4964, 0.4964, 0.4964)$ for the Os atom, as well as $(0.2458, 0.8784, 0.6143)$ for the O atom into the sample polar NCS R3c structure. Then, we by taking the symmetry of the structure into account refresh all the other remaining atomic Li, Os, and O positions. Ultimately, we create a hexagonal supercell from the rhombohedral structure "$\lambda = 0.1$" constructed above, as discussed in Sec. 5.2. The created hexagonal supercell which constitutes the desired intermediate structure "$\lambda = 0.1$" is shown in Fig. SM6.

For constructing the other intermediate structures "$\lambda = 0.2$"", "$\lambda = 0.3$", and ..., "$\lambda = 0.9$", it is enough to increase the vector steps $\boldsymbol{\lambda}_{0.1}$ obtained above for the Li, Os, and O atoms step by step multiplying all the above vector steps $\boldsymbol{\lambda}_{0.1}$ by 2, 3, and ..., 9 and repeat the above procedure 8 times more. All the structures constructed following the above procedure are shown in Fig. SM6. These structures are used in "Uniquifying of spontaneous polarization of $LiOsO_3$ by finding the best branch" of the main text to choose the best branch to overcome the uncertainty problem in the Berry phases and polarizations.

5.5 G-Type antiferromagnetic structure

The $LiOsO_3$ compound contains Os atom. The ground state electronic configuration of Os atom is $[Xe]$: $4f^{14}5d^6 6s^2$. Thus, due to the half-filled 5d shell, it may be expected, at least at low temperatures, to observe a magnetic ordering for $LiOsO_3$, like its other osmium oxides family such as $NaOsO_3$, $Cd_2Os_2O_7$, $BiFeO_3$ and Ba_2YOsO_7 [89, 90, 91, 92, 93]. However, this osmium based material, unlike its family [89, 90, 91, 92, 93], has not been magnetically ordered even at low temperatures down to around 2 K [54, 70]. $LiOsO_3$, due to its crystal structure, has more compact Os-O-Os bounds, compared to the other osmium perovskites. This compact bounds create more stronger Os:5d-O:2p hybridization, leading to a higher effective hopping between Os orbitals. This, in turn, leads to a wider 5d orbitals and thence a more preferable non-magnetic state in $LiOsO_3$, than the narrower 5d orbitals in the other normal osmium perovskites [62]. The non-polar CS state undergoes a continuous structural phase transition at 140 K to the polar NCS state [54]. All these imply that the crystal structure of the NM $LiOsO_3$ compound can stay in the non-polar CS R$\bar{3}$c (polar NCS R3c) phase at the wide temperature interval (140, 300 K] ([2, 140 K]), provided that the other thermodynamic and mechanical conditions can remain unchanged. However, Danilo Puggioni et al. [94], using DFT+DMFT calculations, predicted that the $LiOsO_3$ could be close to the Mott transition at U = 1.25 eV and $J_H = 0.1875$ eV or U = 0.7 eV and $J_H = 0.21$ eV, see Figs. 1(a) and (b) of Ref. [94] where these data were extracted from. Therefore, we have configured the G-type AFM ordering for the compound in question, as well. For the G-AFM configuration, we have used the hexagonal structure, containing 6 Os atoms in its unit cell, where the spins of the nearest-neighbor Os^{5+} ions are oppositely ordered [62], for more information see also Fig. 1 of Ref. [95] for the G-AFM $BiFeO_3$ with the same magnetic structure as the G-AFM $LiOsO_3$.

5.6 Structural and electronic properties of $LiOsO_3$

Here, for the G-AFM configuration, we have used the hexagonal structure containing 30 atoms, as discussed in Sec. 5.2, see also Ref. [95], and for the NM configuration, we have used the rhombohedral structure containing 10 atoms, as discussed in Sec. 5.1. We have calculated the lattice parameter a in Å and the ratio c/a as well as the energy bandgap in eV for the polar and non-polar phases of $LiOsO_3$ using GGA, GGA+U, GGA+U+SO, LDA+U and LDA+U+SO approaches for the G-AFM

Table SM2. Calculated lattice parameter (a) in Å, tetragonality c/a and the energy bandgap (GAP) in eV using the PBE-GGA, GGA+U, and LDA+U functionals, including spin polarization (SP), magnetic ordering (MO), and spin-orbit coupling (SOC). J_H is the Hund's exchange term applied on the d orbitals of the Os^{5+} ions in the unit of the U parameter. The data in () are calculated in the presence of SOC. The experimental and the other theoretical data are given for comparison. We have used $a = 2a_r \cos[(\pi - \alpha_r)/2]$ and $c = 3[a_r^2 - a^2/3]^{1/2}$ relations, as reported in chapter 4, subchapter 4.3, page 40 of Ref. [26], to convert the primitive rhombohedral lattice parameter a_r and the rhombohedral angel α_r to the conventional hexagonal lattice parameters a and c, respectively. The optimized lattice parameters reported in Ref. [67] are divided by 2, due to their $2 \times 2 \times 2$ structure used. The results calculated in the present work are denoted by *.

Phase	Scheme	U(eV)	J_H	SP	SOC	MO	GAP(eV)	a(Å)	c/a	Ref.
R3c	PBE-GGA	-	-	No	No	NM	0.00	5.0768	2.6418	*
	LDA	0.4	0	Yes	No(Yes)	G-AFM	0.00(0.00)	4.9885 (4.9848)	2.6285(2.6455)	*
	LDA	0.8	0	Yes	No(Yes)	G-AFM	0.00(0.00)	4.9866 (4.9856)	2.6346(2.6459)	*
	LDA	1.2	0	Yes	No(Yes)	G-AFM	0.00(0.00)	4.9871(4.9871)	2.6371(2.6453)	*
	LDA	1.6	0	Yes	No(Yes)	G-AFM	0.02(0.00)	4.9882(4.9862)	2.6387(2.6485)	*
	LDA	2.0	0	Yes	No(Yes)	G-AFM	0.12(0.00)	4.9906(4.9928)	2.6379(2.6429)	*
	LDA	2.4	0	Yes	No(Yes)	G-AFM	0.22(0.07)	4.9901(4.9954)	2.6419(2.6424)	*
	LDA	0.4	0.15U	Yes	(Yes)	G-AFM	(0.00)	(4.9846)	(2.6457)	*
	LDA	0.8	0.15U	Yes	(Yes)	G-AFM	(0.00)	(4.9860)	(2.6451)	*
	LDA	1.2	0.15U	Yes	(Yes)	G-AFM	(0.00)	(4.9866)	(2.6457)	*
	LDA	1.6	0.15U	Yes	(Yes)	G-AFM	(0.00)	(5.0015)	(2.6312)	*
	LDA	2.0	0.15U	Yes	(Yes)	G-AFM	(0.01)	(4.9928)	(2.6420)	*
	LDA	2.4	0.15U	Yes	(Yes)	G-AFM	(0.04)	(4.9939)	(2.6434)	*
	PBE-GGA	0.4	0	Yes	No(Yes)	G-AFM	0.07(0.00)	5.0755(5.0796)	2.6420(2.6435)	*
	PBE-GGA	0.8	0	Yes	No(Yes)	G-AFM	0.17(0.00)	5.0769(5.0812)	2.6430(2.6444)	*
	PBE-GGA	1.2	0	Yes	No(Yes)	G-AFM	0.27(0.00)	5.0786(5.0854)	2.6434(2.6414)	*
	PBE-GGA	1.6	0	Yes	No(Yes)	G-AFM	0.38(0.15)	5.0799(5.0867)	2.6446(2.6426)	*
	PBE-GGA	2.0	0	Yes	No(Yes)	G-AFM	0.48(0.22)	5.0812(5.0889)	2.6455(2.6423)	*
	PBE-GGA	2.4	0	Yes	No(Yes)	G-AFM	0.58(0.29)	5.0821(5.0905)	2.6470(2.6427)	*
	PBE-GGA	0.4	0	Yes	No(Yes)	NM	0.00(0.00)	5.0707(5.0751)	2.6373(2.6473)	*
	PBE-GGA	0.8	0	Yes	No(Yes)	NM	0.00(0.00)	5.0710(5.0758)	2.6375(2.6483)	*
	PBE-GGA	1.2	0	Yes	No(Yes)	NM	0.00(0.00)	5.0707(5.0762)	2.6385(2.6519)	*
	PBE-GGA	1.6	0	Yes	No(Yes)	NM	0.00(0.00)	5.0709(5.0706)	2.6389(2.6606)	*
	PBE-GGA	2.0	0	Yes	No(Yes)	NM	0.00(0.00)	5.0709(5.0713)	2.6396(2.6620)	*
	PBE-GGA	2.4	0	Yes	No(Yes)	NM	0.00(0.00)	5.0713(5.0863)	2.6398(2.6498)	*
	LDA	0.4	0	Yes	No(Yes)	NM	0.00(0.00)	4.9843(4.9908)	2.6330(2.6340)	*
	LDA	0.8	0	Yes	No(Yes)	NM	0.00(0.00)	4.9847(4.9897)	2.6332(2.6363)	*
	LDA	1.2	0	Yes	No(Yes)	NM	0.00(0.00)	4.9846(4.9889)	2.6341(2.6382)	*
	LDA	1.6	0	Yes	No(Yes)	NM	0.00(0.00)	4.9846(4.9886)	2.6349(2.6391)	*
	LDA	2.0	0	Yes	No(Yes)	NM	0.00(0.00)	4.9849(4.9899)	2.6353(2.6380)	*
	LDA	2.4	0	Yes	No(Yes)	NM	0.00(0.00)	4.9846(5.0045)	2.6366(2.6239)	*
	PBE-GGA	-	-	No	No	NM	0.00	5.0930	2.6267	[64]
	LDA	2.0	0	Yes	No	NM	0.00	4.9785	2.6423	[67]
	LDA	2.0	0	Yes	No	FM	0.00	4.9711	2.6677	[67]
	LDA	2.0	0	Yes	No	G-AFM	0.12	4.9900	2.6433	[67]
	LDA	2.0	0	Yes	(Yes)	G-AFM	0.21	5.0460[a]	2.6237[a]	[96]
	PBE-GGA	2.0	0	Yes	(Yes)	G-AFM	0.42	5.0460[a]	2.6237[a]	[96]
	PBSOL	2.0	0	Yes	(Yes)	G-AFM	0.34	5.0460[a]	2.6237[a]	[96]
	Exp.	-	-	No	No	NM	0.00	5.0460	2.6237	[54]
$R\bar{3}c$	PBE	-	-	No	No	NM	0.00	5.1360	2.5824	*
	LDA	0.4	0	Yes	No(Yes)	G-AFM	0.00(0.00)	5.0589(5.0551)	2.5499(2.5677)	*
	LDA	0.8	0	Yes	No(Yes)	G-AFM	0.00(0.00)	5.0569(5.0568)	2.5562(2.5668)	*
	LDA	1.2	0	Yes	No(Yes)	G-AFM	0.00(0.00)	5.0545(5.0566)	2.5634(2.5686)	*
	LDA	1.6	0	Yes	No(Yes)	G-AFM	0.04(0.00)	5.0569(5.0531)	2.5631(2.5748)	*
	LDA	2.0	0	Yes	No(Yes)	G-AFM	0.14(0.003)	5.0571(5.0607)	2.5658(2.5691)	*
	LDA	2.4	0	Yes	No(Yes)	G-AFM	0.24(0.10)	5.0595(5.0641)	2.5652(2.5673)	*
	LDA	0.4	0.15U	Yes	(Yes)	G-AFM	(0.00)	(5.0559)	(2.5664)	*
	LDA	0.8	0.15U	Yes	(Yes)	G-AFM	(0.00)	(5.0561)	(2.5675)	*
	LDA	1.2	0.15U	Yes	(Yes)	G-AFM	(0.00)	(5.0571)	(2.5674)	*
	LDA	1.6	0.15U	Yes	(Yes)	G-AFM	(0.00)	(5.0538)	(2.5740)	*
	LDA	2.0	0.15U	Yes	(Yes)	G-AFM	(0.00)	(5.0659)	(2.5601)	*
	LDA	2.4	0.15U	Yes	(Yes)	G-AFM	(0.07)	(5.0619)	(2.5694)	*
	PBE-GGA	0.4	0	Yes	No(Yes)	G-AFM	0.08(0.00)	5.1374 (5.1498)	2.5769(2.5756)	*
	PBE-GGA	0.8	0	Yes	No(Yes)	G-AFM	0.19(0.00)	5.1395(5.1553)	2.5769(2.5708)	*
	PBE-GGA	1.2	0	Yes	No(Yes)	G-AFM	0.29(0.13)	5.1401(5.1550)	2.5790(2.5744)	*
	PBE-GGA	1.6	0	Yes	No(Yes)	G-AFM	0.39(018)	5.1412(5.1561)	2.5805(2.5768)	*
	PBE-GGA	2.0	0	Yes	No(Yes)	G-AFM	0.50(0.25)	5.1417(5.1555)	2.5827(2.5806)	*
	PBE-GGA	2.4	0	Yes	No(Yes)	G-AFM	0.60(0.31)	5.1416(5.1471)	2.5856(2.5868)	*
	PBE-GGA	0.4	0	Yes	No(Yes)	NM	0.00(0.00)	5.1357(5.1333)	2.5671(2.5862)	*
	PBE-GGA	0.8	0	Yes	No(Yes)	NM	0.00(0.00)	5.1360(5.1348)	2.5674(2.5864)	*
	PBE-GGA	1.2	0	Yes	No(Yes)	NM	0.00(0.00)	5.1357(5.1356)	2.5686(2.5871)	*
	PBE-GGA	1.6	0	Yes	No(Yes)	NM	0.00(0.00)	5.1354(5.1351)	2.5699(2.5897)	*
	PBE-GGA	2.0	0	Yes	No(Yes)	NM	0.00(0.00)	5.1357(5.1354)	2.5701(2.5915)	*
	PBE-GGA	2.4	0	Yes	No(Yes)	NM	0.00(0.00)	5.1358(5.1361)	2.5709(2.5926)	*
	LDA	0.4	0	Yes	No(Yes)	NM	0.00(0.00)	5.0575(5.0526)	2.5499(2.5710)	*
	LDA	0.8	0	Yes	No(Yes)	NM	0.00(0.00)	5.0578(5.0532)	2.5503(2.5707)	*
	LDA	1.2	0	Yes	No(Yes)	NM	0.00(0.00)	5.0578(5.0519)	2.5511(2.5738)	*
	LDA	1.6	0	Yes	No(Yes)	NM	0.00(0.00)	5.0580(5.0518)	2.5517(2.5752)	*
	LDA	2.0	0	Yes	No(Yes)	NM	0.00(0.00)	5.0579(5.0541)	2.5527(2.5761)	*
	LDA	2.4	0	Yes	No(Yes)	NM	0.00(0.00)	5.0575(5.0525)	2.5541(2.5807)	*
	PBE-GGA	-	-	No	No	NM	0.00	5.1450	2.5675	[64]
	Exp.	-	-	No	No	NM	0.00	5.0640	2.6088	[54]

[a] Experimental lattice parameters, as reported in Ref. [54], were used for the calculations.

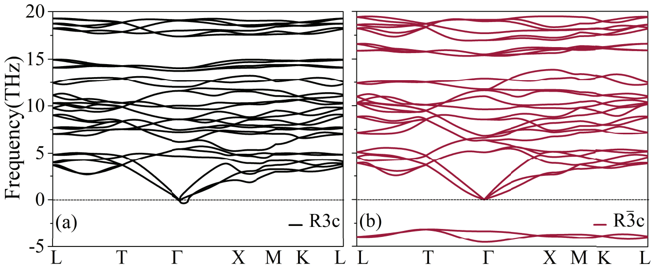

Figure SM7. Phonon dispersion curves calculated for (a) polar NCS R3c phase, and (b) non-polar CS R$\bar{3}$c phase of $LiOsO_3$. The DFT [51, 52] calculations are performed using PBE-GGA [35]. The phonon calculations are performed using frozen-phonon method as implemented in the Phonopy package [87] by constructing a supercell containing 80 atoms from the rhombohedral unit cell of $LiOsO_3$.

and NM configurations. In addition, the effect of Hund's exchange term J_H on the lattice parameters and energy bandgap are considered for the spin-polarized LDA+U approach. By starting from the experimental lattice parameters, the theoretical lattice parameters and atomic positions of $LiOsO_3$ are fully optimized and relaxed with the criterion of 0.5 mRy/Bohr on the exerted forces. The calculated results together with the available experimental and theoretical data are tabulated in Table SM2 for both the NCS (R3c) and CS (R$\bar{3}$c) structures.

The results, consistent with the available experimental and theoretical data presented in Table SM2, show that the GGA overestimates and LDA underestimates the lattice constant, in agreement with the well-known underbinding and overbinding problems of these functionals, respectively. The calculated results for the lattice parameter a show that the GGA+U (LDA+U+SO) with $U = 0.4$ eV ($U = 2$ eV and $J_H = 0.15U$) for the NM (G-AFM) state of the R3c phase are in better agreement with the experimental data. The results show that a increases as U increases in most of the approximations. The GGA results show that the spin-orbit coupling (SOC) causes to increase the lattice constant. The spin-polarized LDA+U results show that Hund's exchange parameter J_H may not considerably affect the lattice constant.

The c/a ratio can be better reproduced by the LDA+U with smaller U without SOC in R3c phase, but it is slightly overestimated by PBE-GGA compared to the experimental value of 2.6237 reported in Ref.[54]. This overestimation, as discussed in Ref. [92] for the perovskite ferroelectrics, is closely related to the off-center displacements of the Os^{5+} ions. As discussed in Sec. 3.3, the Os^{5+} ions are off-centered due to nonequivalent Os-O and Os-Li bonds in the R3c phase. LDA tends to homogenize the electron density. Thus, LDA can decrease the difference between the shorter and longer bonds in both the O-Os-O and Li-Os-Li. In contrast to the LDA, however, the PBE-GGA predicts stronger bonding and thereby increases the difference between bonds along the distortion direction. This leads to a bigger c/a ratio in the polarization direction of the R3c structure compared to the ratio predicted by LDA and experimental value. In the R$\bar{3}$c phase, as discussed in Sec. 3.3, Os^{5+} ions are not off-centered in both the O-Os-O and Li-Os-Li bonds. Therefore, LDA and PBE-GGA both lead to homogeneous bonds, however, the LDA more underestimates the c/a due its functional properties. Thus, the PBE-GGA can better reproduce the c/a ratio for the R$\bar{3}$c phase. In the metallic states, SOC causes to increase the c/a ratio compared to the related calculation in the absence of SOC. In the G-AFM configuration, the system can become an insulator by GGA+U with a suitable U. By the spin-polarized LDA approach without SOC, the bandgap is opened at $U_{eff} = U - J_H = U - 0 = U = 1.6$ eV, but by the LDA+U+SO, the bandgap is opened at $U = 2$ eV. By the LDA+U+SO including the Hund's exchange J_H, the bandgap is opened at $U = 2$ eV, where $U_{eff} = 1.7$ eV and $J_H = 0.3$ eV. Consistent with the latter result, in Ref. [67], by the LDA+U approach the metal-insulator transition is reported to occur for $U_{eff} > 1.75$ eV. The data extracted from Fig. 2 of Ref. [96] show that this transition by LDA+U+SO approach occurs for $U_{eff} > 1.6$ eV. By the GGA+U approach, the metal-insulator transition occurs at very small U about 0.2 eV. The GGA+U+SO causes to observe this transition at $U_{eff} = 1.6$ eV. The data extracted from Fig. 2 of Ref.[96], shows that this transition by GGA+U occurs for $U_{eff} = 0$ and by GGA+U+SO approach occurs for $U_{eff} = 1.6$ eV. Our energy bandgap calculated by the LDA+U with $U_{eff} = 2$ eV is in agreement with the results reported in Ref. [67], see Table SM2. The energy bandgaps reported in Ref. [96] calculated by the GGA+U+SO with $U = 2$ eV and LDA+U+SO with $U = 2$ eV are also consistent with our results calculated by the same functionals, see Table SM2. The last point is that the metal-insulator transition of the non-polar structure is almost similar to the polar structure.

6 DYNAMICAL STABILITY (INSTABILITY) OF POLAR NCS R3c (NON-POLAR CS R$\bar{3}$c) PHASE IN $LiOsO_3$

We have checked the dynamical stability of the non-polar CS R$\bar{3}$c and polar NCS R3c phases by calculating their phonon spectra using frozen phonon approach employing Phonopy package [87]. To this end, we construct a $(2 \times 2 \times 2)$-supercell

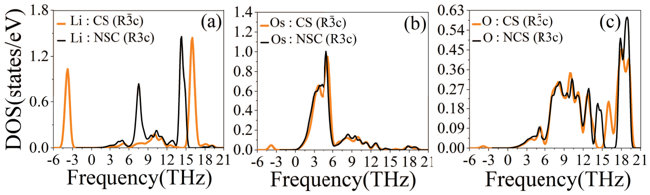

Figure SM8. The partial phonon DOSs calculated individually for (a) Li, and (b) Os, as well as (c) O atoms in both the polar NCS R3c and non-polar CS R$\bar{3}$c phases of $LiOsO_3$. The DFT [51, 52] calculations are performed using PBE-GGA [35]. The phonon calculations are performed using frozen-phonon method as implemented in the Phonopy package [87] by constructing a supercell containing 80 atoms from the rhombohedral unit cell of $LiOsO_3$.

containing 80 atoms from the rhombohedral structure containing 10 atoms for the non-polar CS R$\bar{3}$c phase. Similarly, we have made another supercell for the polar NCS R3c phase. Then, following the theory of lattice dynamics [97, 98], we calculate the forces exerted on every atom in both of the CS and NCS phases. To this end, first an atom is displaced, and the forces exerted on every atom are calculated within DFT [51, 52] using the Hellman-Feynman theorem [99, 100, 101, 102]. Then, we construct the dynamical matrices, $D^{kk'}_{\mu\nu}(\boldsymbol{q}_j)$, for both of the CS and NCS phases individually, where μ & ν indexes indicate the Cartesian directions of the forces exerted on the atoms and vary from 1 to 3, and k & k' indexes numerate the number of atoms in the unit cell. By this way, which is the so-called frozen-phonon method, we calculate the normal modes, $\omega(\boldsymbol{q}_j)$, at any wavevector $\boldsymbol{q}_j$ for the maximum 3-acoustical and $(3N_p - 3)$-optical branches j, $j = 1,2,3;4,\ldots,3N_p$, by finding the roots of the eigenvalues, $\omega^2(\boldsymbol{q}_j)$, of the dynamical matrices, *viz.* $\det[D^{kk'}_{\mu\nu}(\boldsymbol{q}_j) - \delta_{\mu\nu}\delta_{kk'}\omega^2(\boldsymbol{q}_j)] = 0$, for both the polar and non-polar phases. The dynamical matrix is Hermitian and whence its eigenvalues are real, *viz.* $[D(\boldsymbol{q}_j)]^\dagger = [D^{kk'}_{\nu\mu}(\boldsymbol{q}_j)]^* = D^{kk'}_{\mu\nu}(\boldsymbol{q}_j) \Rightarrow [\omega^2(\boldsymbol{q}_j)]^* = \omega^2(\boldsymbol{q}_j)$. Therefore, the normal modes $\omega(\boldsymbol{q}_j)$ should be either purely real numbers or purely imaginary numbers. However, if $\omega(\boldsymbol{q}_j)$ are purely imaginary numbers, i.e. $[\omega(\boldsymbol{q}_j)]^* = -\omega(\boldsymbol{q}_j)$, the amplitude of the phonon wave after a long time can exponentially diverge to infinity which is not physically acceptable. Thus, if $[\omega(\boldsymbol{q}_j)]^* = -\omega(\boldsymbol{q}_j)$, the system is said to be dynamically unstable. It is customary that in such cases, if any, the purely imaginary frequencies are shown by negative values together with the purely real frequencies which are shown by positive values. According to this custom presentation, a negative (a positive) frequency is identical to an imaginary (a real) frequency. Following this custom presentation, we have plotted the phonon dispersion curves calculated for the polar NCS R3c phase in Fig. SM7(a) and for the non-polar CS R$\bar{3}$c in Fig. SM7(b) along the symmetrical paths of the 1BZ. Furthermore, we have also presented the partial atomic phonon DOSs calculated for both of the phases in Fig. SM8. In these calculations the longitudinal-optical (LO)-transverse-optical (TO), LO-TO, splitting, are not considered because the NCS and CS are both metals. The LO-TO splitting originates from the long-range Coulomb interactions in the system [103, 104]. Although TO mode may not generally depend on LO mode, the softest TO mode can be strongly coupled with the hardest LO mode, leading to a large LO-TO splitting [103]. In metals due to the screening effect, however, the range of Coulomb interactions is substantially reduced by an exponential Yukawa decay factor including magnitude of the Thomas-Fermi wavevector as the scaling constant [10]. Therefore, LO-TO may not be very strong for the metallic phases of $LiOsO_3$.

K. Parlinski, Z. Q. Li, and Y. Kawazoe [105] employing LDA using Vanderbilt ultrasoft pseudopotentials as implemented in the VASP [57, 58, 59, 60, 61] code taking LO-TO splitting into consideration in the ionic $LiNbO_3$ compound have shown that the soft mode A_{2u}, in the paraelectric phase, can be responsible for the paraelectric $\rightleftharpoons$ ferroelectric phase transition. Hyunsu Sim and Bog G. Kim [64], employing PBE-GGA [35] and the projector augmented-wave method [106], which is a generalization of the pseudopotential and linear augmented-plane-wave method, as implemented in the VASP [57, 58, 59, 60, 61] code together with the use of Phonopy package [87], have also attributed the NCS transition and the spontaneous symmetry breaking in $LiOsO_3$ to the soft A_{2u}, i.e. Γ_2, mode at the 1BZ center Γ, with ignoring LO-TO splitting in this metal. Feng Jin et al. [53] experimentally using polarized and Raman measurements and theoretically employing PBE-GGA [35] and the projector augmented-wave method [106] as implemented in the VASP [57, 58, 59, 60, 61] have reported mode frequencies for the high-temperature CS and low-temperature NCS phases of $LiOsO_3$.

The results show that all the branches lie in the positive frequencies for the polar NCS phase of $LiOsO_3$, see Fig. SM7(a). For the non-polar CS R$\bar{3}$c phase, however, the results show two negative frequency branches, i.e. imaginary frequency branches, see Fig. SM7(b). Our results due to the imaginary frequencies, as shown in Fig. SM7(b), in agreement with the results presented in Fig. 3(a) of Ref. [64] for different k-paths from our selected k-paths, also confirm that the non-polar CS R$\bar{3}$c phase is dynamically unstable. In contrast to the latter phase, however, the results show that the polar NCS phase of $LiOsO_3$ is dynamically stable. This is also consistent with the results reported in Ref. [64], compare Fig. SM7(a) with Fig. 3(c) of Ref. [64], though our k-paths selected in Fig. SM7(a) and (b) are not the same as those selected in Figs. 3(a) and (c) of Ref. [64].

To find the source(s) of the instability in the CS phase, let us shed light into the phonon DOSs. The results show that the instability of the CS phase mainly originates from Li atoms, see the high peak of the orange Li-DOS occurring at the negative frequencies around -3.76 THz in Fig. SM8(a). The value of this negative frequency of the CS phase is consistent with the value of -124.32 cm^{-1} extracted from Fig. 3(b) of Ref. [64], *viz.* -124.32 cm^{-1} $\times$ (0.03 THz/cm^{-1}) $= -3.73$ THz ≈ -3.76 THz. This value is also comparable with the value of -138 cm^{-1} reported in Table III of Ref. [53] for the $^1A_{2u}$ mode of the high-temperature CS phase, *viz.* -138 cm^{-1} $\times$ (0.03 THz/cm^{-1}) $= -4.14$ THz ≈ -3.76 THz. The heights of the peaks of the phonon DOSs appearing at negative frequencies for Os and O atoms are substantially smaller than that of Li atom. Thus, the instabilities imposed by Os, as shown in Fig. SM8(b), and O atoms, as shown in Fig. SM8(c), are almost ignorable compared to that imposed by Li atom. Therefore, Li atoms can be considered as the main source of the instability. Several peaks at positive frequencies are observed above the Fermi level for the partial Li DOS of the CS phase. The highest peak occurs at 15.99 THz, and right after that a short peak appears at 18.16 THz. The positive frequencies of 15.99 THz and 18.16 THz are also comparable with the experimental (theoretical) values of 491.7(515) cm^{-1} and 642.9(607) cm^{-1}, respectively, reported in Table III of Ref. [53] for the ^3E_g and ^4E_g modes of the high-temperature CS phase, *viz.* 491.7(515) cm^{-1} $\times$ (0.03 THz/cm^{-1}) $=$ 14.75(15.45) THz $\approx$ 15.99 THz and 642.9(607) cm^{-1} $\times$ (0.03 THz/cm^{-1}) $=$ 19.29(18.21) THz $\approx$ 18.16 THz. For the NCS phase, all the peaks of the Li phonon DOS occur at positive frequencies, see black curves in Fig. SM8(a). The highest, and shortest, as well as middle peaks of Li phonon DOS for the NCS phase, as shown in Fig. SM8(a), occur at positive frequencies of 14.29 THz, and 4.92 THz, as well as 7.53 THz, respectively. The frequency corresponding to the highest peak is in excellent agreement with the frequency of 476.59 cm^{-1} extracted from Fig. 3(d) of Ref. [64], *viz.* 476.59 cm^{-1} $\times$ (0.03 THz/cm^{-1}) $=$ 14.30 THz $\approx$ 14.29 THz. This value is also consistent with the experimental (theoretical) value of 488.2(471) cm^{-1} reported in Table III of Ref. [53] for the ^7E mode of the low-temperature NCS, *viz.* 488.2(471) cm^{-1} $\times$ (0.03 THz/cm^{-1}) $=$ 14.6(14.1) THz $\approx$ 14.29 THz. The frequency corresponding to the shortest peak is nearly close to the frequency of 153.19 cm^{-1} extracted from Fig. 3(d) of Ref. [64], *viz.* 153.19 cm^{-1} $\times$ (0.03 THz/cm^{-1}) $=$ 4.59 THz $\approx$ 4.92 THz. This value is also consistent with the experimental (theoretical) value of 174(176) cm^{-1} reported in Table III of Ref. [53] for the ^1E mode of the low-temperature NCS, *viz.* 174(176) cm^{-1} $\times$ (0.03 THz/cm^{-1}) $=$ 5.2(5.3) THz $\approx$ 4.92 THz. The frequency corresponding to the shortest peak is fairly in agreement with the frequency of 204.26 cm^{-1} extracted from Fig. 3(d) of Ref. [64], *viz.* 204.26 cm^{-1} $\times$ (0.03 THz/cm^{-1}) $=$ 6.13 THz $\approx$ 7.53 THz. This value is also approximately consistent with the experimental (theoretical) value of 208.6(202) cm^{-1} reported in Table III of Ref. [53] for the ^2E mode of the low-temperature NCS, *viz.* 208.6(202) cm^{-1} $\times$ (0.03 THz/cm^{-1}) $=$ 6.3(6.1) THz $\approx$ 7.53 THz. All these evidences verify that the phase transition from the dynamically unstable non-polar high-temperature CS phase with space group R$\bar{3}$c and point group D_{3d} can be feasible to the dynamically stable polar low-temperature NCS phase with space group R3c and point group C_{3v}, mostly due to the vibrations of Li atoms.

7 EMPIRICAL REMARKS

7.1 Derivation of empirical Eq. (11) from Eqs. (9) and (10) of the main text

Let us, first, represent Eq. (9) of the main text, taking the experimental error $\Delta\mathrm{T}_c$ into account, as follows:

$$\mathrm{T}_c + \Delta\mathrm{T}_c = (2.00 \pm 0.09) \times 10^4 \mathscr{Z}^2, \tag{53}$$

where $\mathscr{Z} := \Delta z$, $\Delta\mathrm{T}_c = 0.09 \times 10^4 \mathscr{Z}^2$. Regardless of the experimental error, the main part of Eq. (9) of the main text or Eq. (53) reads as:

$$\mathrm{T}_c = 2.00 \times 10^4 \mathscr{Z}^2. \tag{54}$$

Similarly, Eq. (9) of the main text, taking the experimental error $\Delta\mathscr{P} := \Delta(\Delta\mathrm{P})$ into account, can be represented as follows:

$$\mathscr{P} \pm \Delta\mathscr{P} = (258 \pm 9)\mathscr{Z}, \tag{55}$$

where $\mathscr{P} := \Delta\mathrm{P}$, $\Delta\mathscr{P} = 9\mathscr{Z}$. Regardless of the experimental error, the main part of Eq. (11) of the main text or Eq. (55) reads as:

$$\mathscr{P} = 258\mathscr{Z}, \tag{56}$$

By eliminating $\mathscr{Z}$ between Eqs. (54) and (56), we have:

$$\mathrm{T}_c = 2.00/258^2 \times 10^4 \mathscr{P}^2 \approx 0.300\mathscr{P}^2, \tag{57}$$

which is identical to the main part of Eq. (11) of the main text, i.e. compare $0.300(\Delta\mathrm{P})^2$ in Eq. (11) of the main text with Eq. (57). The remaining task is to reproduce the experimental error part of Eq. (11) of the main text, $0.02(\Delta\mathrm{P})^2 \equiv 0.02\mathscr{P}^2$.

To this end, let us differentiate from both sides of Eqs. (54) and (56):

$$\Delta T_c = 4.00 \times 10^4 \mathscr{Z} \Delta \mathscr{Z}, \quad (58)$$

$$\Delta \mathscr{P} = 258 \Delta \mathscr{Z}. \quad (59)$$

By substituting $\mathscr{Z}$ from Eq. (56) and $\Delta \mathscr{Z}$ from Eq. (59) into Eq. (58), we have:

$$\Delta T_c = 4.00/258^2 \times 10^4 (\Delta \mathscr{P}) \mathscr{P}. \quad (60)$$

By using $\Delta \mathscr{P} = 9\mathscr{Z}$, Eq. (60) can be represented as:

$$\Delta T_c = 9 \times 4.00/258^2 \times 10^4 \mathscr{Z} \mathscr{P}. \quad (61)$$

Now, by eliminating $\mathscr{Z}$ between Eqs. (56) and (61), we lead to:

$$\Delta T_c = 9 \times 4.00/258^3 \times 10^4 \mathscr{P}^2 \approx 0.02 \mathscr{P}^2, \quad (62)$$

which is identical to the error part of Eq. (11) of the main text, i.e. compare $0.02(\Delta P)^2$ in Eq. (11) of the main text with Eq. (62). By adding the error part, Eq. (62), to the main part, Eq. (57), we have:

$$T_c + \Delta T_c = 0.300 \mathscr{P}^2 + 0.02 \mathscr{P}^2, \quad (63)$$

which can be simplified to complete the derivation of Eq. (11) of the main text. To this end, in Eq. (63), we set back $\mathscr{P}$ to ΔP, and notice that T_c in Eq. (11) of the main text is equivalent to $T_c + \Delta T_c$ in Eq. (53) or (63). Hence:

$$T_c = (0.300 + 0.02)(\Delta P)^2. \quad (64)$$

7.2 Derivation of ΔP and its accuracy from empirical Eq. (12) of the main text

Let us, first, represent Eq. (12) of the main text, taking the experimental error ΔT_c into account, as follows:

$$T_c + \Delta T_c = (0.303 \pm 0.018) \mathscr{P}^2, \quad (65)$$

where $\Delta T_c = 0.018 \mathscr{P}^2$. Regardless of the experimental error, the main part of Eq. (12) of the main text or Eq. (65) can be written as:

$$T_c = 0.303 \mathscr{P}^2,$$
$$\mathscr{P} = \sqrt{\tfrac{T_c}{0.303}} = \sqrt{\tfrac{140}{0.303}} = 21.495\ \mu C/cm^2, \quad (66)$$

where the experimental transition temperature $T_c = 140$ K [54] is used to evaluate $\mathscr{P}$ which constitutes the main part of the empirical spontaneous polarization $\mathscr{P} = \Delta P = 21.495\ \mu C/cm^2 \approx 21.50\ \mu C/cm^2$, as reported in "Empirical verification: quadratic order" and tabulated in Table 3 of the main text. The remaining task is to obtain the experimental error part of the spontaneous polarization, $\Delta \mathscr{P}$. To this end, let us differentiate from both sides of Eq. (66):

$$\Delta \mathscr{P} = \frac{\Delta T_c}{2\sqrt{0.303 T_c}}. \quad (67)$$

By substituting $\sqrt{T_c}$ from Eq. (66) as $21.495\sqrt{0.303}$ and ΔT_c from Eq. (65) or Eq. 12 of the main text as $\Delta T_c = 0.018 \times 10^4 \mathscr{P}^2$ into Eq. (67) as well as setting $\mathscr{P}$ from Eq. (66) to $21.495\ \mu C/cm^2$, we find:

$$\Delta \mathscr{P} = \frac{0.018 \times 21.495}{2 \times 0.303} = 0.6385\ \mu C/cm^2, \quad (68)$$

which constitutes the error part of the empirical spontaneous polarization $\Delta \mathscr{P} = \Delta(\Delta P) = 0.6385\ \mu C/cm^2 \approx 0.64\ \mu C/cm^2$, as reported in "Empirical verification: quadratic order" and tabulated in Table 3 of the main text. By adding the error part, Eq. (68), to the main part, Eq. (66), we have:

$$\mathscr{P} + \Delta \mathscr{P} = (21.50 \pm 0.64)\ \mu C/cm^2, \quad (69)$$

which can be simply written as $\Delta P = (21.50 \pm 0.64)\ \mu C/cm^2$, by setting back $\mathscr{P}$ to ΔP, and notice that $\mathscr{P} + \Delta \mathscr{P}$ in Eq. (69) is nothing more than ΔP.

References

1. Resta, R. Theory of the electric polarization in crystals. *Ferroelectrics* **136**, 51–55, DOI: 10.1080/00150199208016065 (1992). https://doi.org/10.1080/00150199208016065.

2. King-Smith, R. D. & Vanderbilt, D. Theory of polarization of crystalline solids. *Phys. Rev. B* **47**, 1651–1654, DOI: 10.1103/PhysRevB.47.1651 (1993).

3. Resta, R. Macroscopic electric polarization as a geometric quantum phase. *Europhys. Lett. (EPL)* **22**, 133–138, DOI: 10.1209/0295-5075/22/2/010 (1993).

4. Resta, R., Posternak, M. & Baldereschi, A. Towards a quantum theory of polarization in ferroelectrics: The case of $knbo_3$. *Phys. Rev. Lett.* **70**, 1010–1013, DOI: 10.1103/PhysRevLett.70.1010 (1993).

5. Vanderbilt, D. & King-Smith, R. D. Electric polarization as a bulk quantity and its relation to surface charge. *Phys. Rev. B* **48**, 4442–4455, DOI: 10.1103/PhysRevB.48.4442 (1993).

6. Resta, R. Macroscopic polarization in crystalline dielectrics: the geometric phase approach. *Rev. Mod. Phys.* **66**, 899 (1994).

7. Resta, R. & Vanderbilt, D. *Theory of Polarization: A Modern Approach*, 31–68 (Springer Berlin Heidelberg, Berlin, Heidelberg, 2007).

8. Vanderbilt, D. *Berry Phases in Electronic Structure Theory: Electric Polarization, Orbital Magnetization and Topological Insulators* (Cambridge University Press, 2018).

9. Spaldin, N. A. A beginner's guide to the modern theory of polarization. *J. Solid State Chem.* **195**, 2–10 (2012).

10. Ashcroft, N. & Mermin, N. *Solid State Physics*. HRW international editions (Holt, Rinehart and Winston, 1976).

11. Mostofi, A. A. *et al.* wannier90: A tool for obtaining maximally-localised wannier functions. *Comput. Phys. Commun.* **178**, 685 – 699, DOI: https://doi.org/10.1016/j.cpc.2007.11.016 (2008).

12. Mostofi, A. A. *et al.* An updated version of wannier90: A tool for obtaining maximally-localised wannier functions. *Comput. Phys. Commun.* **185**, 2309 – 2310, DOI: https://doi.org/10.1016/j.cpc.2014.05.003 (2014).

13. Marzari, N. & Vanderbilt, D. Maximally localized generalized wannier functions for composite energy bands. *Phys. Rev. B* **56**, 12847–12865, DOI: 10.1103/PhysRevB.56.12847 (1997).

14. Souza, I., Marzari, N. & Vanderbilt, D. Maximally localized wannier functions for entangled energy bands. *Phys. Rev. B* **65**, 035109, DOI: 10.1103/PhysRevB.65.035109 (2001).

15. Pizzi, G. *et al.* Wannier90 as a community code: new features and applications. *J. Physics: Condens. Matter* **32**, 165902, DOI: 10.1088/1361-648x/ab51ff (2020).

16. Kuneš, J. *et al.* Wien2wannier: From linearized augmented plane waves to maximally localized wannier functions. *Comput. Phys. Commun.* **181**, 1888–1895 (2010).

17. Mollabashi, L. & Jalali-Asadabadi, S. Crystal fields of lithium rare-earth tetrafluorides and multiplet splitting of the +3 rare-earth ions. *Phys. Rev. B* **102**, 045120, DOI: 10.1103/PhysRevB.102.045120 (2020).

18. Shapere, A. & Wilczek, F. *Geometric phases in Physics*, vol. 5 (World Scientific, Singapore, 1989).

19. Resta, R. Manifestations of berry's phase in molecules and condensed matter. *J. Physics: Condens. Matter* **12**, R107–R143, DOI: 10.1088/0953-8984/12/9/201 (2000).

20. Vanderbilt, D. Berry-phase theory of proper piezoelectric response. *J. Phys. Chem. Solids* **61**, 147–151 (2000).

21. Ahmed, S. *et al.* Berrypi: A software for studying polarization of crystalline solids with wien2k density functional all-electron package. *Comput. Phys. Commun.* **184**, 647–651 (2013).

22. Marzari, N., Mostofi, A. A., Yates, J. R., Souza, I. & Vanderbilt, D. Maximally localized wannier functions: Theory and applications. *Rev. Mod. Phys.* **84**, 1419–1475, DOI: 10.1103/RevModPhys.84.1419 (2012).

23. Wannier, G. H. The structure of electronic excitation levels in insulating crystals. *Phys. Rev.* **52**, 191–197, DOI: 10.1103/PhysRev.52.191 (1937).

24. Mahan, G. D. *Many-Particle Physics*. Third Edition (Kluwer Academic/Plenum Publishers, New York, Boston, Dordrecht, London, Moscow, 2000).

25. Sakurai, J. J. *Modern Quantum Mechanics; Revised Edition* (Addison-Wesley, Reading, MA, 1994).

26. Blaha, P. *et al.* Wien2k. *WIEN2k, An Augment. Plane Wave + Local Orbitals Program for Calc. Cryst. Prop.* (2021).

27. Blaha, P. *et al.* Wien2k: An apw+lo program for calculating the properties of solids. *The J. Chem. Phys.* **152**, 074101, DOI: 10.1063/1.5143061 (2020). https://doi.org/10.1063/1.5143061.

28. Sjöstedt, E., Nordström, L. & Singh, D. An alternative way of linearizing the augmented plane-wave method. *Solid State Commun.* **114**, 15–20 (2000).

29. Madsen, G. K. H., Blaha, P., Schwarz, K., Sjöstedt, E. & Nordström, L. Efficient linearization of the augmented plane-wave method. *Phys. Rev. B* **64**, 195134, DOI: 10.1103/PhysRevB.64.195134 (2001).

30. Cottenier, S. *Density Functional Theory and the family of (L)APW-methods: a step-by-step introduction* (Instituut voor Kern-en Stralingsfysica, KU Leuven, Belgium, 2nd Edition).

31. Thygesen, K. S., Hansen, L. B. & Jacobsen, K. W. Partly occupied wannier functions. *Phys. Rev. Lett.* **94**, 026405, DOI: 10.1103/PhysRevLett.94.026405 (2005).

32. Thygesen, K. S., Hansen, L. B. & Jacobsen, K. W. Partly occupied wannier functions: Construction and applications. *Phys. Rev. B* **72**, 125119, DOI: 10.1103/PhysRevB.72.125119 (2005).

33. Thygesen, K. & Jacobsen, K. Molecular transport calculations with wannier functions. *Chem. Phys.* **319**, 111–125, DOI: https://doi.org/10.1016/j.chemphys.2005.05.032 (2005). Molecular Charge Transfer in Condensed Media - from Physics and Chemistry to Biology and Nanoengineering in honour of Alexander M. Kuznetsov on his 65th birthday.

34. Calzolari, A., Marzari, N., Souza, I. & Buongiorno Nardelli, M. Ab initio transport properties of nanostructures from maximally localized wannier functions. *Phys. Rev. B* **69**, 035108, DOI: 10.1103/PhysRevB.69.035108 (2004).

35. Perdew, J. P., Burke, K. & Ernzerhof, M. Generalized gradient approximation made simple. *Phys. Rev. Lett.* **77**, 3865 (1996).

36. Anisimov, V. I., Zaanen, J. & Andersen, O. K. Band theory and mott insulators: Hubbard u instead of stoner i. *Phys. Rev. B* **44**, 943–954, DOI: 10.1103/PhysRevB.44.943 (1991).

37. Anisimov, V. I., Solovyev, I. V., Korotin, M. A., Czyżyk, M. T. & Sawatzky, G. A. Density-functional theory and nio photoemission spectra. *Phys. Rev. B* **48**, 16929–16934, DOI: 10.1103/PhysRevB.48.16929 (1993).

38. Czyżyk, M. T. & Sawatzky, G. A. Local-density functional and on-site correlations: The electronic structure of la_2cuo_4 and $lacuo_3$. *Phys. Rev. B* **49**, 14211–14228, DOI: 10.1103/PhysRevB.49.14211 (1994).

39. Anisimov, V. I., Aryasetiawan, F. & Lichtenstein, A. I. First-principles calculations of the electronic structure and spectra of strongly correlated systems: thelda + u method. *J. Physics: Condens. Matter* **9**, 767–808, DOI: 10.1088/0953-8984/9/4/002 (1997).

40. Grindlay, J. *An Introduction to the Phenomenological Theory of Ferroelectricity: International Series of Monographs In: Natural Philosophy* (Pergamon Press: Oxford, 1970).

41. Kristoffel, N. & Konsin, P. Vibronic theory of structural phase transitions and displacive ferroelectrics. *physica status solidi (b)* **149**, 11–40 (1988).

42. Cohen, R. E. Theory of ferroelectrics: a vision for the next decade and beyond. *J. Phys. Chem. Solids* **61**, 139–146 (2000).

43. Zhang, W. & Xiong, R.-G. Ferroelectric metal–organic frameworks. *Chem. Rev.* **112**, 1163–1195, DOI: 10.1021/cr200174w (2012). PMID: 21939288, https://doi.org/10.1021/cr200174w.

44. Berry, M. V. Quantal phase factors accompanying adiabatic changes. *Proc. Royal Soc. London. A. Math. Phys. Sci.* **392**, 45–57, DOI: 10.1098/rspa.1984.0023 (1984). https://royalsocietypublishing.org/doi/pdf/10.1098/rspa.1984.0023.

45. Berry, M. Classical adiabatic angles and quantal adiabatic phase. *J. Phys. A: Math. Gen.* **18**, 15 (1985).

46. Kato, T. On the adiabatic theorem of quantum mechanics. *J. Phys. Soc. Jpn.* **5**, 435–439, DOI: 10.1143/JPSJ.5.435 (1950). https://doi.org/10.1143/JPSJ.5.435.

47. Simon, B. Holonomy, the quantum adiabatic theorem, and berry's phase. *Phys. Rev. Lett.* **51**, 2167–2170, DOI: 10.1103/PhysRevLett.51.2167 (1983).

48. Pizzi, G. *et al.* Wannier90 as a community code: new features and applications. *J. Physics: Condens. Matter* **32**, 165902, DOI: 10.1088/1361-648x/ab51ff (2020).

49. Filippetti, A., Fiorentini, V., Ricci, F., Delugas, P. & Íñiguez, J. Prediction of a native ferroelectric metal. *Nat. Commun.* **7**, 11211 (2016).

50. Anderson, P. W. & Blount, E. Symmetry considerations on martensitic transformations:" ferroelectric" metals? *Phys. Rev. Lett.* **14**, 217 (1965).

51. Hohenberg, P. & Kohn, W. Inhomogeneous electron gas. *Phys. Rev.* **136**, B864–B871, DOI: 10.1103/PhysRev.136.B864 (1964).

52. Kohn, W. & Sham, L. J. Self-consistent equations including exchange and correlation effects. *Phys. Rev.* **140**, A1133–A1138, DOI: 10.1103/PhysRev.140.A1133 (1965).

53. Jin, F. *et al.* Raman phonons in the ferroelectric-like metal lioso 3. *Phys. Rev. B* **93**, 064303 (2016).

54. Shi, Y. *et al.* A ferroelectric-like structural transition in a metal. *Nat. Mater.* **12**, 1024 (2013).

55. Kirschner, F. K. *et al.* Static and fluctuating magnetic moments in the ferroelectric metal lioso3. In *Proceedings of the 14th International Conference on Muon Spin Rotation, Relaxation and Resonance (μSR2017)*, 011013 (2018).

56. Liu, H. M. *et al.* Metallic ferroelectricity induced by anisotropic unscreened coulomb interaction in $lioso_3$. *Phys. Rev. B* **91**, 064104, DOI: 10.1103/PhysRevB.91.064104 (2015).

57. Kresse, G. & Hafner, J. Ab initio molecular dynamics for liquid metals. *Phys. Rev. B* **47**, 558–561, DOI: 10.1103/PhysRevB.47.558 (1993).

58. Kresse, G. & Hafner, J. Ab initio molecular-dynamics simulation of the liquid-metal–amorphous-semiconductor transition in germanium. *Phys. Rev. B* **49**, 14251–14269, DOI: 10.1103/PhysRevB.49.14251 (1994).

59. Kresse, G. & Furthmüller, J. Efficient iterative schemes for ab initio total-energy calculations using a plane-wave basis set. *Phys. Rev. B* **54**, 11169–11186, DOI: 10.1103/PhysRevB.54.11169 (1996).

60. Kresse, G. & Furthmüller, J. Efficiency of ab-initio total energy calculations for metals and semiconductors using a plane-wave basis set. *Comput. Mater. Sci.* **6**, 15 – 50, DOI: https://doi.org/10.1016/0927-0256(96)00008-0 (1996).

61. Kresse, G. & Joubert, D. From ultrasoft pseudopotentials to the projector augmented-wave method. *Phys. Rev. B* **59**, 1758–1775, DOI: 10.1103/PhysRevB.59.1758 (1999).

62. Zhang, Y. *et al.* Strain-induced slater transition in polar metal $lioso_3$. *Phys. Status Solidi (RRL)–Rapid Res. Lett.* **13**, 1900436 (2019).

63. Giovannetti, G. & Capone, M. Dual nature of the ferroelectric and metallic state in $lioso_3$. *Phys. Rev. B* **90**, 195113 (2014).

64. Sim, H. & Kim, B. G. First-principles study of octahedral tilting and ferroelectric-like transition in metallic $lioso_3$. *Phys. Rev. B* **89**, 201107(R), DOI: 10.1103/PhysRevB.89.201107 (2014).

65. Yao, Q., Wu, H., Deng, K. & Kan, E. Ferroelectric-like structural transition in metallic lioso3. *RSC Adv.* **4**, 26843–26846 (2014).

66. Puggioni, D., Giovannetti, G., Capone, M. & Rondinelli, J. M. Design of a mott multiferroic from a nonmagnetic polar metal. *Phys. Rev. Lett.* **115**, 087202 (2015).

67. He, C., Ma, Z., Sun, B.-Z., Li, Q. & Wu, K. Ab-initio study on the electronic, optical and ferroelectric properties of $lioso_3$. *Comput. Mater. Sci.* **105**, 11–17 (2015).

68. Lo Vecchio, I. *et al.* Electronic correlations in the ferroelectric metallic state of $lioso_3$. *Phys. Rev. B* **93**, 161113(R), DOI: 10.1103/PhysRevB.93.161113 (2016).

69. Li, P., Ren, X., Guo, G.-C. & He, L. The origin of hyperferroelectricity in libo 3 (b= v, nb, ta, os). *Sci. Reports* **6**, 34085 (2016).

70. Zhang, Y. *et al.* Possible origin of the absence of magnetic order in $lioso_3$: Spin–orbit coupling controlled ground state. *Phys. Status Solidi (RRL)–Rapid Res. Lett.* **12**, 1800396 (2018).

71. Lu, J. *et al.* Ferroelectricity with asymmetric hysteresis in metallic $lioso_3$ ultrathin films. *Phys. Rev. Lett.* **122**, 227601, DOI: 10.1103/PhysRevLett.122.227601 (2019).

72. Laurita, N. J. *et al.* Evidence for the weakly coupled electron mechanism in an anderson-blount polar metal. *Nat. Commun.* **10**, 3217, DOI: 10.1038/s41467-019-11172-2 (2019).

73. Keppens, V. Structural transitions:'ferroelectricity'in a metal. *Nat. Mater.* **12**, 952 (2013).

74. Puggioni, D. & Rondinelli, J. M. Designing a robustly metallic noncenstrosymmetric ruthenate oxide with large thermopower anisotropy. *Nat. Commun.* **5**, 3432, DOI: 10.1038/ncomms4432 (2014).

75. Xiang, H. J. Origin of polar distortion in $linbo_3$-type "ferroelectric" metals: Role of *a*-site instability and short-range interactions. *Phys. Reviw B* **90**, 094108 (2014).

76. Fujioka, J. *et al.* Ferroelectric-like metallic state in electron doped $BaTiO_3$. *Sci. Reports* **5**, 13207 (2015).

77. Kim, T. *et al.* Polar metals by geometric design. *Nature* **533**, 68 (2016).

78. Benedek, N. A. & Birol, T. 'ferroelectric'metals reexamined: fundamental mechanisms and design considerations for new materials. *J. Mater. Chem. C* **4**, 4000–4015 (2016).

79. Takahashi, K. *et al.* Polar metal phase stabilized in strained la-doped batio 3 films. *Sci. Reports* **7**, 4631 (2017).

80. Shi, P.-P. *et al.* Symmetry breaking in molecular ferroelectrics. *Chem. Soc. Rev.* **45**, 3811–3827, DOI: 10.1039/C5CS00308C (2016).

81. Zak, J. Berry's phase for energy bands in solids. *Phys. Rev. Lett.* **62**, 2747–2750, DOI: 10.1103/PhysRevLett.62.2747 (1989).

82. Andrinopoulos, L., Hine, N. D. & Mostofi, A. A. Calculating dispersion interactions using maximally localized wannier functions. *The J. Chem. Phys.* **135**, 154105 (2011).

83. Iannuzzi, M. & Parrinello, M. Wave-function localization in reciprocal space. *Phys. Rev. B* **66**, 155209, DOI: 10.1103/PhysRevB.66.155209 (2002).

84. Giovannetti, G. & Capone, M. Dual nature of the ferroelectric and metallic state in lioso 3. *Phys. Rev. B* **90**, 195113 (2014).

85. Perdew, J. P. & Zunger, A. Self-interaction correction to density-functional approximations for many-electron systems. *Phys. Rev. B* **23**, 5048–5079, DOI: 10.1103/PhysRevB.23.5048 (1981).

86. Schwarz, K., Blaha, P. & Madsen, G. K. Electronic structure calculations of solids using the wien2k package for material sciences. *Comput. Phys. Commun.* **147**, 71–76 (2002).

87. Togo, A., Oba, F. & Tanaka, I. First-principles calculations of the ferroelastic transition between rutile-type and cacl2-type sio2 at high pressures. *Phys. Rev. B* **78**, 134106 (2008).

88. Mehl, M. J. *et al.* The aflow library of crystallographic prototypes: Part 1. *Comput. Mater. Sci.* **136**, S1–S828, DOI: https://doi.org/10.1016/j.commatsci.2017.01.017 (2017).

89. Shi, Y. G. *et al.* Continuous metal-insulator transition of the antiferromagnetic perovskite $naoso_3$. *Phys. Rev. B* **80**, 161104(R), DOI: 10.1103/PhysRevB.80.161104 (2009).

90. Calder, S. *et al.* Spin-orbit-driven magnetic structure and excitation in the 5 d pyrochlore cd 2 os 2 o 7. *Nat. communications* **7**, 1–8 (2016).

91. Lebeugle, D., Colson, D., Forget, A. & Viret, M. Very large spontaneous electric polarization in $bifeo_3$ single crystals at room temperature and its evolution under cycling fields. *Appl. Phys. Lett.* **91**, 022907 (2007).

92. Zhang, Y., Sun, J., Perdew, J. P. & Wu, X. Comparative first-principles studies of prototypical ferroelectric materials by lda, gga, and scan meta-gga. *Phys. Rev. B* **96**, 035143 (2017).

93. Kermarrec, E. *et al.* Frustrated fcc antiferromagnet ba_2yoso_6: Structural characterization, magnetic properties, and neutron scattering studies. *Phys. Rev. B* **91**, 075133, DOI: 10.1103/PhysRevB.91.075133 (2015).

94. Puggioni, D., Giovannetti, G., Capone, M. & Rondinelli, J. M. Design of a mott multiferroic from a nonmagnetic polar metal. *Phys. Rev. Lett.* **115**, 087202 (2015).

95. Park, J. *et al.* Magnetoelectric feedback among magnetic order, polarization, and lattice in multiferroic bifeo3. *J. Phys. Soc. Jpn.* **80**, 114714 (2011).

96. Liu, P. *et al.* Comparative ab initio study of the structural, electronic, magnetic, and dynamical properties of $lioso_3$ and $naoso_3$. *Phys. Rev. Mater.* **4**, 045001, DOI: 10.1103/PhysRevMaterials.4.045001 (2020).

97. Brüesch, P. *Phonons: Theory and Experiments I: Lattice Dynamics and Modelsof Interatomic Forces* (Springer-Verlag Berlin Heidelberg New York, 1982), 1st ed. edn.

98. Callaway, J. *Quantum Theory of the Solid State* (Academic Press, New York, 1991), 2nd ed. edn.

99. Güttinger, P. Das verhalten von atomen im magnetischen drehfeld. *Zeitschrift für Physik* **73**, 169–184, DOI: 10.1007/BF01351211 (1932).

100. Clusius. Einführung in die quantenchemie. von h. hellmann. 350 s.,43 abb., 35 tab. franz deuticke, leipzig u. wien 1937. pr. geh. rm. 20,-. geb. rm. 22,-. *Angewandte Chemie* **54**, 156–156 (1941).

101. Feynman, R. P. Forces in molecules. *Phys. Rev.* **56**, 340–343, DOI: 10.1103/PhysRev.56.340 (1939).

102. Di Ventra, M. & Pantelides, S. T. Hellmann-feynman theorem and the definition of forces in quantum time-dependent and transport problems. *Phys. Rev. B* **61**, 16207 (2000).

103. Zhong, W., King-Smith, R. D. & Vanderbilt, D. Giant lo-to splittings in perovskite ferroelectrics. *Phys. Rev. Lett.* **72**, 3618–3621, DOI: 10.1103/PhysRevLett.72.3618 (1994).

104. Shafique, A. & Shin, Y.-H. The effect of non-analytical corrections on the phononic thermal transport in inx (x = s, se, te) monolayers. *Sci. Reports* **10**, 1093, DOI: 10.1038/s41598-020-57644-0 (2020).

105. Parlinski, K., Li, Z. Q. & Kawazoe, Y. Ab initio calculations of phonons in $linbo_3$. *Phys. Rev. B* **61**, 272–278, DOI: 10.1103/PhysRevB.61.272 (2000).

106. Blöchl, P. E. Projector augmented-wave method. *Phys. Rev. B* **50**, 17953–17979, DOI: 10.1103/PhysRevB.50.17953 (1994).
